# Organoplatinum(II) Type II Immunogenic Cell Death Inducers Target Protein Tyrosine Phosphatase 1B to Drive Immunogenicity

**DOI:** 10.1021/jacs.5c18904

**Published:** 2025-12-23

**Authors:** Jiao Xia Zou, Pavel A. Ivanov-Rostovtsev, Jemma Arakelyan, Maria V. Babak, Wee Han Ang

**Affiliations:** † Department of Chemistry, 37580 National University of Singapore, 4 Science Drive 2, Singapore 117543, Singapore; ‡ Drug Discovery Lab, Department of Chemistry, 53025 City University of Hong Kong, 83 Tat Chee Avenue, Hong Kong, SAR 999077, People’s Republic of China; ∥ NUS Graduate School - Integrative Sciences and Engineering Programme (ISEP), 37580 National University of Singapore, 21 Lower Kent Ridge Road, Singapore 119077, Singapore

## Abstract

Immunogenic cell death (ICD) inducers are valuable chemotherapeutic agents that elicit protective immune responses against tumors. Although many ICD inducers have emerged in recent years, specific molecular targets directly associated with ICD remain relatively unexplored. Here, two Type II ICD inducers, **Pt-NHC** and **PlatinER** (**Pt-ER**), were validated as *bona fide* ICD inducers with the ability to establish immunity against colorectal cancer *in vivo*. Based on **Pt-ER**, several ICD-inducing photoactivable probes were designed to capture their potential targets. By integrating quantitative proteomics analysis with biochemical assays, we identified PTP1B as a direct target of **Pt-ER** that engages in ICD. Both **Pt-ER** and **Pt-NHC** were shown to directly interact with PTP1B and inhibit its enzymatic activity. The suppression of PTP1B, by either genetic knockdown or pharmacological inhibition, enhanced the immunogenicity of tumor cells by increasing surface-exposed calreticulin and phagocytosis of cancer cells. Bioinformatic analysis also revealed that PTP1B plays a role in tumor progression and immune regulation in colorectal cancer. Therefore, our study first reveals a previously unrecognized role of PTP1B in modulating ICD and highlights its potential therapeutic values in cancer chemoimmunotherapy.

## Graphical abstract

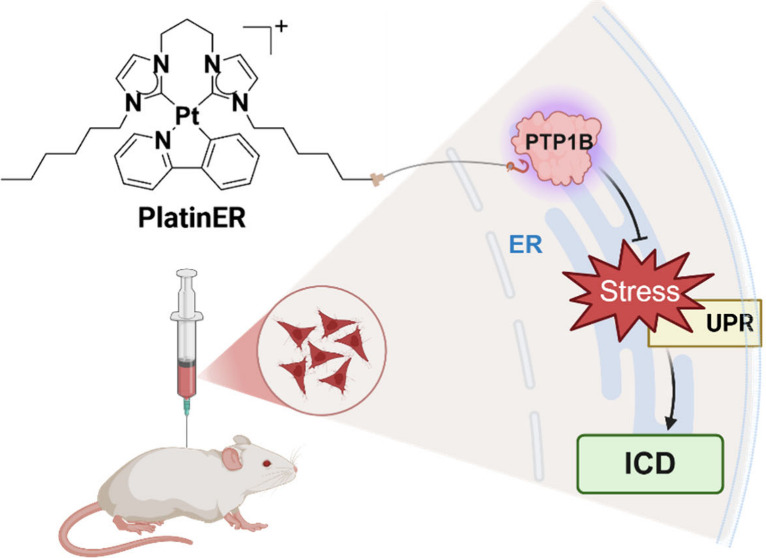

## Introduction

Immunogenic cell death (ICD) is a unique form of regulated cell death that stimulates an adaptive immune response in immunocompetent hosts.^1–5^ ICD inducers not only exert direct cytotoxic effects but also promote the establishment of protective antitumor immunity. Owing to this dual functionality, ICD-based therapies hold great potential for improving clinical therapeutic outcomes of current cancer chemotherapies.^6,7^ Since its discovery by Kroemer and co-workers in 2005,^8^ a wide range of ICD-inducing strategies have been identified, including certain chemotherapeutic agents,^9^ photodynamic therapy,^10,11^ various physical therapies^12,13^ and many more.^14^ ICD inducers can be broadly classified into Type I and Type II, based on whether or not they act directly on the endoplasmic reticulum (ER).^4^ Type II inducers directly target ER, where ICD is initiated through the translocation of soluble calreticulin (CRT) to the cell surface early in the process. In contrast, Type I ICD inducers act on other cellular components and induce ICD as a collateral effect.

Metal complexes represent an important class of ICD inducers owing to their ability to disrupt redox balance and cause cellular stress, processes commonly associated with ICD induction.^15–18^ A wide variety of metal-based ICD inducers have been discovered, including those based on Pt,^19–22^ Ir,^23–26^ Ru,^27–30^ Au,^31–35^ Re,^36^ Cu,^37,38^ Mn^39^ and Zn.^40^ Earlier, we identified the Pt-carbene complex **Pt-NHC** to be an effective Type II ICD inducer, the first such metal complex reported in the literature, from a panel of Pt-containing clinical drugs and active anticancer agents.^22^ A follow-on structure–activity relationship study revealed **PlatinER** (**Pt-ER**) to be an optimized Type II ICD inducer with superior ability to elicit *in vitro* ICD via ROS-driven ER stress.^21^ Both **Pt-NHC** and **Pt-ER** were further evaluated for combination therapy and demonstrated to enhance antitumor efficacy and the ability to reprogram the immunosuppressive microenvironment *in vivo*.^41,42^ Yet, the molecular targets underlying their immunogenic mechanism remain elusive.

To date, several secondary targets outside ER have been linked to Type I ICD induction (Figure 1).^43–46^ For example, transmembrane receptor tyrosine kinase EPHB4 ^45^ and NUAK family SNF1-like kinase 1 (NUAK1)^44^ were identified as secondary targets of ICD induction by RNA interference or CRISPR-Cas9 screening, and their inhibition provokes ICD accompanied by characteristic hallmarks such as surface exposure of CRT, extracellular release of high mobility group box 1 (HMGB1) and secretion of adenosine triphosphate (ATP). More recently, lysosomal Cathepsin D was identified as a molecular target of an alkaloid-modified Ir­(III) complex via thermal proteome profiling. Subsequent inhibition of Cathepsin D with pepstatin A and myricitrin resulted in CRT surface exposure.^47^ In contrast, primary targets of Type II ICD inducers, including **Pt-NHC** and **Pt-ER**, within the ER are largely unexplored. Thus far, the only ER protein which has been found to be a direct target engaged in Type II ICD induction (by Zou et al.) is binding immunoglobulin protein (BiP, also known as GRP78/HSPA5), a molecular chaperone modulating ER stress.^26,48^

**Figure 1:**
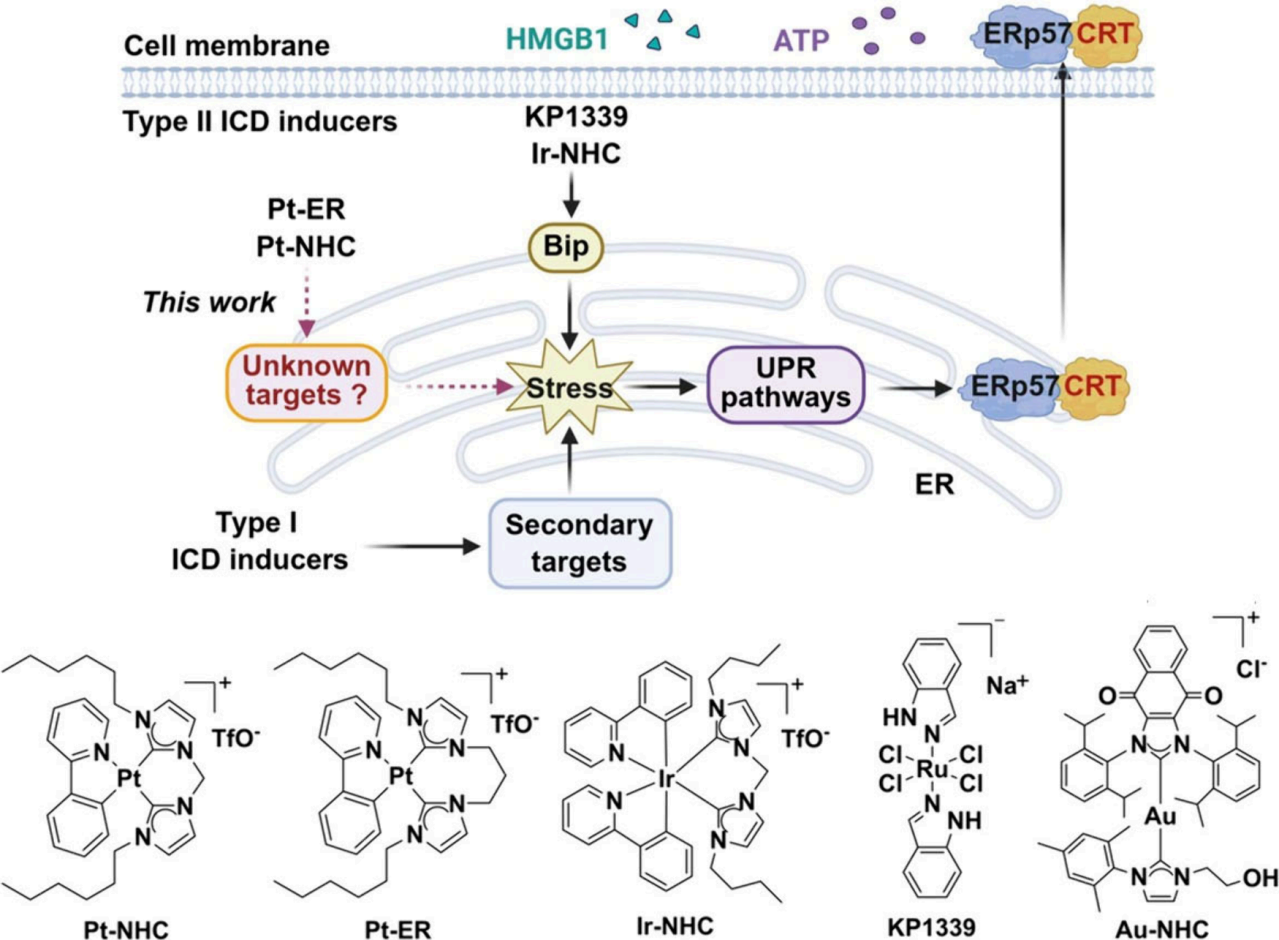
**Molecular targets and mechanism of action of ICD induced by representative metal-based complexes.** ERp57: protein disulfide-isomerase [ERp57 forms discrete complexes with calreticulin and co-translocated onto the cell surface upon ICD]; HMGB1: High Mobility Group Box 1; ATP: adenosine triphosphate; Bip: binding immunoglobulin protein; UPR: unfolded protein response. Pt-NHC, Pt-ER, Ir-NHC, KP1339 and Au-NHC are ICD inducers. Created with the assistance of BioRender.

In this work, we identified ER-resident protein tyrosine phosphatase 1B (PTP1B) as a direct mechanistic target of organoplatinum Type II ICD inducers **Pt-NHC** and **Pt-ER**. Through chemoproteomic and bioinformatic analyses, gene knockdown and various biochemical assays, we demonstrate that these Pt complexes engage PTP1B within the ER to prime the immunogenicity of tumor cell death. Furthermore, we provide experimental validation that **Pt-NHC** and **Pt-ER** serve as cancer vaccines *in vivo*, effectively preventing tumor occurrence and growth in mice. These findings establish PTP1B as a key regulator of ICD and a promising target for cancer immunotherapy. Moreover, these results provide the rationale for the future development of efficient Type II ICD inducers.

## Results

### *In Vivo* ICD Activity of **Pt-NHC** and **Pt-ER**

Our previous work demonstrated that **Pt-NHC** and **Pt-ER** induced robust ICD in CT26 colon cancer cells *in vitro*, characterized by enhanced phagocytosis and the induction of key damage-associated molecular patterns (DAMPs), including ecto-CRT translocation, HMGB1 release and ATP secretion.^21,22^ However, their *in vivo* ICD-inducing capacity remains unverified. To confirm this, we employed the gold-standard vaccination model, which involves injecting immunogenic dying cells into immunocompetent mice and monitoring subsequent tumor growth (Figure 2A).^49^ We evaluated the ICD-inducing activity of **Pt-NHC** and **Pt-ER** in comparison with PBS (negative control) and the clinically approved ICD inducer doxorubicin (positive control). Mice vaccinated with **Pt-ER** and doxorubicin exhibited robust antitumor immunity, significantly outperforming the control group (Figure 2B). At day 35 post-challenge, 60% of **Pt-ER**- and doxorubicin-vaccinated mice remained tumor-free (3 out of 5 mice), in sharp contrast to 0% in PBS controls (0 out of 5 mice). **Pt-NHC** also exhibited significant ICD-inducing capacity (40% tumor-free survival, 2 out of 5 mice), though less potent than **Pt-ER**, consistent with their established *in vitro* ICD properties. Additionally, both organoplatinum compounds significantly delayed tumor onset (**Pt-NHC**: Days 13–23; **Pt-ER**: Days 23–39 vs PBS controls: 8–12 days). At the end point for the control group, the mean tumor volume was 1.4- and 5-fold lower in **Pt-NHC**- and **Pt-ER**-vaccinated mice (ca. 1205 mm^3^ and ca. 327 mm^3^, respectively) vs PBS controls (ca. 1696 mm^3^) (Figure 2C). Collectively, these results validate both **Pt-NHC** and **Pt-ER** as *bona fide* ICD inducers.

**Figure 2:**
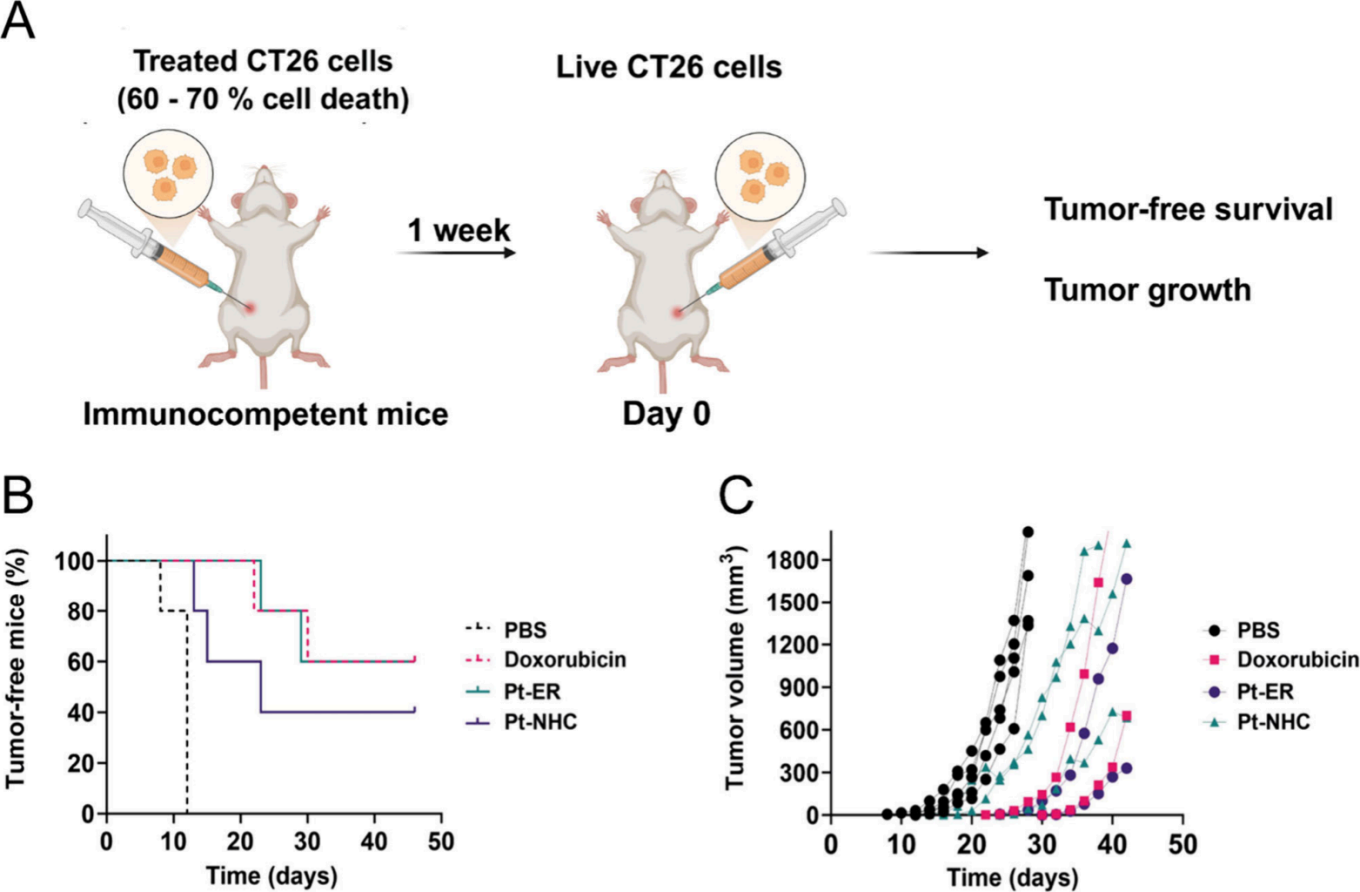
***In vivo* vaccination model.** (A) Schematic diagram illustrating the *in vivo* experiment. Mice (*n* = 5) were vaccinated in the right flank with 3 × 10^5^ CT26 cells pretreated with **Pt-NHC**, **Pt-ER**, doxorubicin or PBS (60%–70% cell death). One week later, mice were rechallenged in the left flank with 3 × 10^5^ live CT26 cells. Day 0 indicates rechallenge. Created with the assistance of BioRender. (B) Tumor-free survival following vaccination. (C) Tumor growth kinetics in non-responsive mice. Volume measurements reflect tumor progression in vaccinated mice that developed palpable tumors post-rechallenge.

### Development of ICD-Inducing Photoaffinity-Based Probes for Target Identification

Given the superior *in vivo* ICD-inducing efficacy of **Pt-ER**, we developed photoaffinity-based probes (AfBPs) to identify its direct protein targets.^50^ Using **Pt-ER** as the core scaffold, 4 AfBPs were designed with either photoreactive diazirine or benzophenone as labels and a bioorthogonal alkyne handle for affinity pull-down via click reaction with biotin (Figure 3A). **P-1**, **P-2a** and **P-2b** were featured with these functional moieties at their two pendant alkyl arms, while **P-3** bore the functional fragment at the C3 linker between the carbene ligands. These AfBPs were prepared using a post-functionalization strategy by installing photoreactive fragments and a bioorthogonal tag through an amide coupling or substitution reaction on **Pt-ER** analogues bearing −OH and −COOH groups (Supplementary Schemes 1–3). All synthesized probes were isolated with good purity and fully characterized using ^1^H NMR, ^13^C NMR, ESI-MS and RP-HPLC (Supplementary Figures 1–27). All probes demonstrated good aqueous stability over a 24-h period (Supplementary Figure 28) and exhibited cytotoxicity comparable to that of **Pt-ER** in human colon cancer HCT116 cells (Figure 3B and Supplementary Table 1).

**Figure 3:**
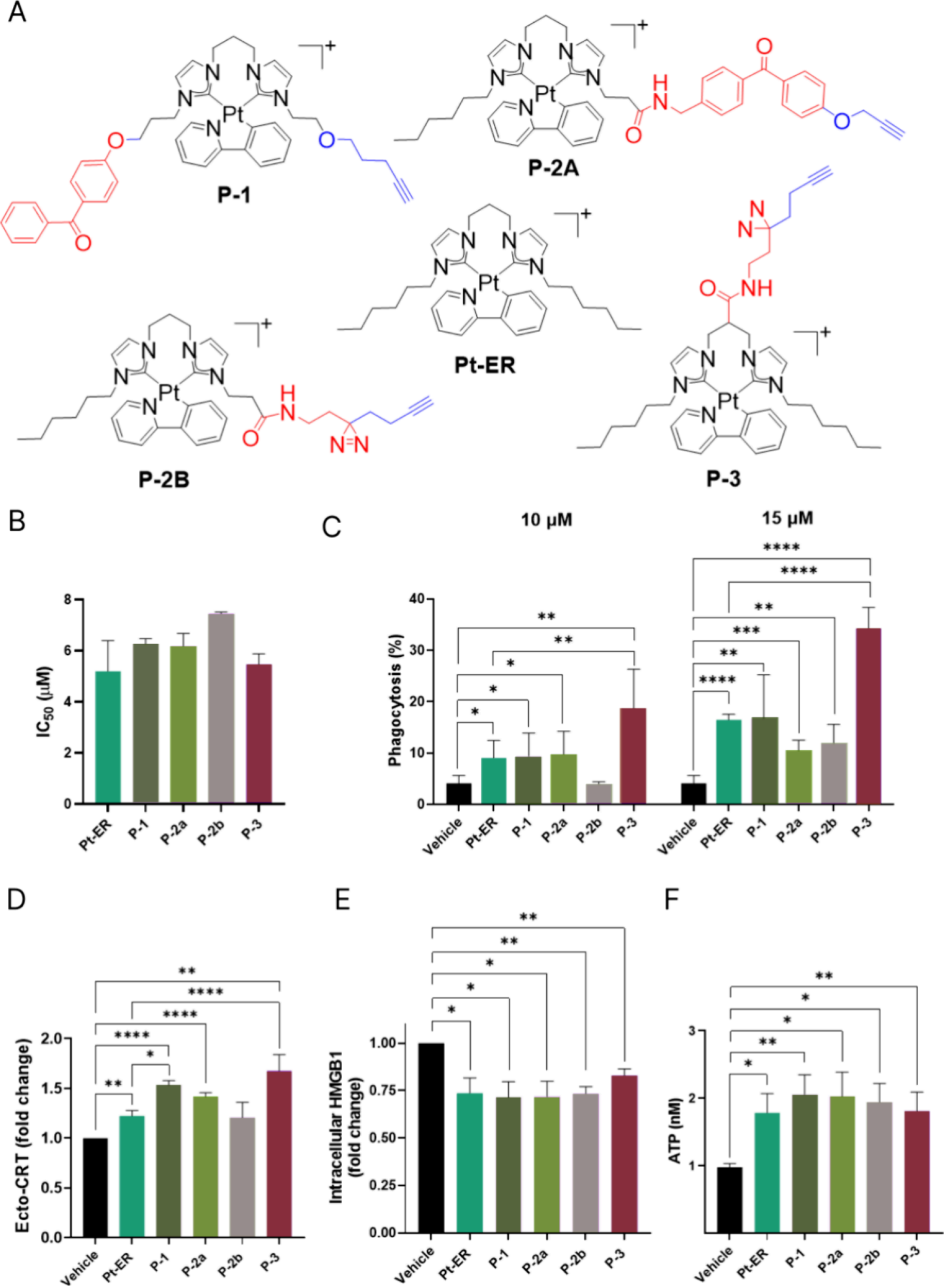
***In vitro* induction of ICD by the designed photoaffinity-based probes (AfBPs).** (A) Chemical structures of **Pt-ER** and novel AfBPs. (B) Cytotoxicity of the studied probes. (C) Flow cytometry analysis of phagocytosis of HCT116 cells by U937 monocytes-derived macrophages. U937 macrophages stained with non-toxic cell tracker green dye CMFDA were co-incubated with cell tracker red dye CMPTX-stained HCT116 cells treated with indicated compounds for 4 h. (D) Flow cytometry analysis of ecto-CRT levels on the surface of live HCT116 cells upon treatment with **Pt-ER** and AfBPs at 5 μM for 4 h. Data is shown as a fold change of mean fluorescence intensity normalized to vehicle (DMSO) control. (E) Intracellular HMGB1 levels upon treatment with 2.5 or 5 μM compounds for 24 h, determined by flow cytometry. The level of HMGB1 is shown as fold change (FC) of mean fluorescence intensity (MFI) to vehicle (DMSO) control. (F) Upon treatment with indicated compounds for 14 h, culture medium was collected and the subjected to ATP bioluminescence assay. Error bars: S.D., *n* = 3 independent repeats. Two-tailed, unpaired Student’s *t* test between vehicle and treatment, *p < 0.05, **p < 0.01, ***p < 0.001, ****p < 0.0001.

To evaluate whether the biorthogonal handle modifications compromise the ability of **Pt-ER** to induce ICD, we assessed the ability of AfBP probes to stimulate phagocytosis of HCT116 cells by U937 monocyte-derived macrophages in comparison with unmodified **Pt-ER** using flow cytometry. The engulfment of immunogenic dying cancer cells by macrophages initiates antitumor immune responses, linking phagocytosis to protective immunity.^22,51^ After 4 h treatment, all AfBPs promoted phagocytosis in a concentration-dependent manner (10–15 μM), matching **Pt-ER** efficacy (Figure 3C). **P-3** at 15 μM showed maximal activity, surpassing that of **Pt-ER** by 2.3-fold. Subsequently, we evaluated the ability of **P-1**, **P-2a**, **P-2b** and **P-3** to elicit three key DAMP signals in HCT116 cells against unmodified **Pt-ER** as a positive control. **P-1**, **P-2a** and **P-3** (5 μM, 4 h) induced surface CRT translocation to levels comparable to that observed with **Pt-ER**, significantly exceeding the DMSO control (Figure 3D). All AfBPs induced substantial extracellular HMGB1 release, leading to the depletion of intracellular HMGB1 levels after 24 h (Figure 3E) and significantly enhanced ATP secretion into the culture medium following 14 h of exposure (Figure 3F).

### Target Identification of **Pt-ER** via TMT Quantitative Proteomics Analysis

To probe each scaffold modification and minimize binding interference, we selected **P-1**, **P-2b** and **P-3** for photoaffinity-based target profiling. HCT116 cells were incubated with each probe (15 μM, 1 h), followed by UV irradiation (365 nm, 20 min) and click reaction with biotin-azide (100 μM, 1.5 h), followed by biotin–neutravidin-based enrichment (Figure 4A).^52^ After extensive washing to remove non-specific binding, the bound proteins were digested and covalently labeled using tandem mass tag (TMT) reagents. TMT-based quantitative proteomics analysis identified over 1,800 unique proteins in three biological replicates (Figure 4B). Among these, several hundred proteins (640 proteins by **P-1**; 545 by **P-2b**; 850 by **P-3**) were substantially enriched by more than 5-fold (*p* < 10^–7^). Assuming that these probes interacted with the same protein targets to elicit ICD, we prioritized overlapping protein targets enriched across all three probes (515 proteins, Supplementary Table 2). Subsequently, Gene Ontology (GO) enrichment analysis of these 515 common proteins identified the main “enriched” biological processes as translation, cytoplasmic translation, proton transmembrane transport, mRNA processing, mRNA splicing, RNA splicing and protein transport (Supplementary Figure 29, Supplementary Table 3). Most of the enriched molecular functions were related to protein and RNA binding. GO analysis of cellular components revealed that the overlapping proteins were distributed in various compartments such as the ER, membrane, nucleus and cytoplasm.

**Figure 4:**
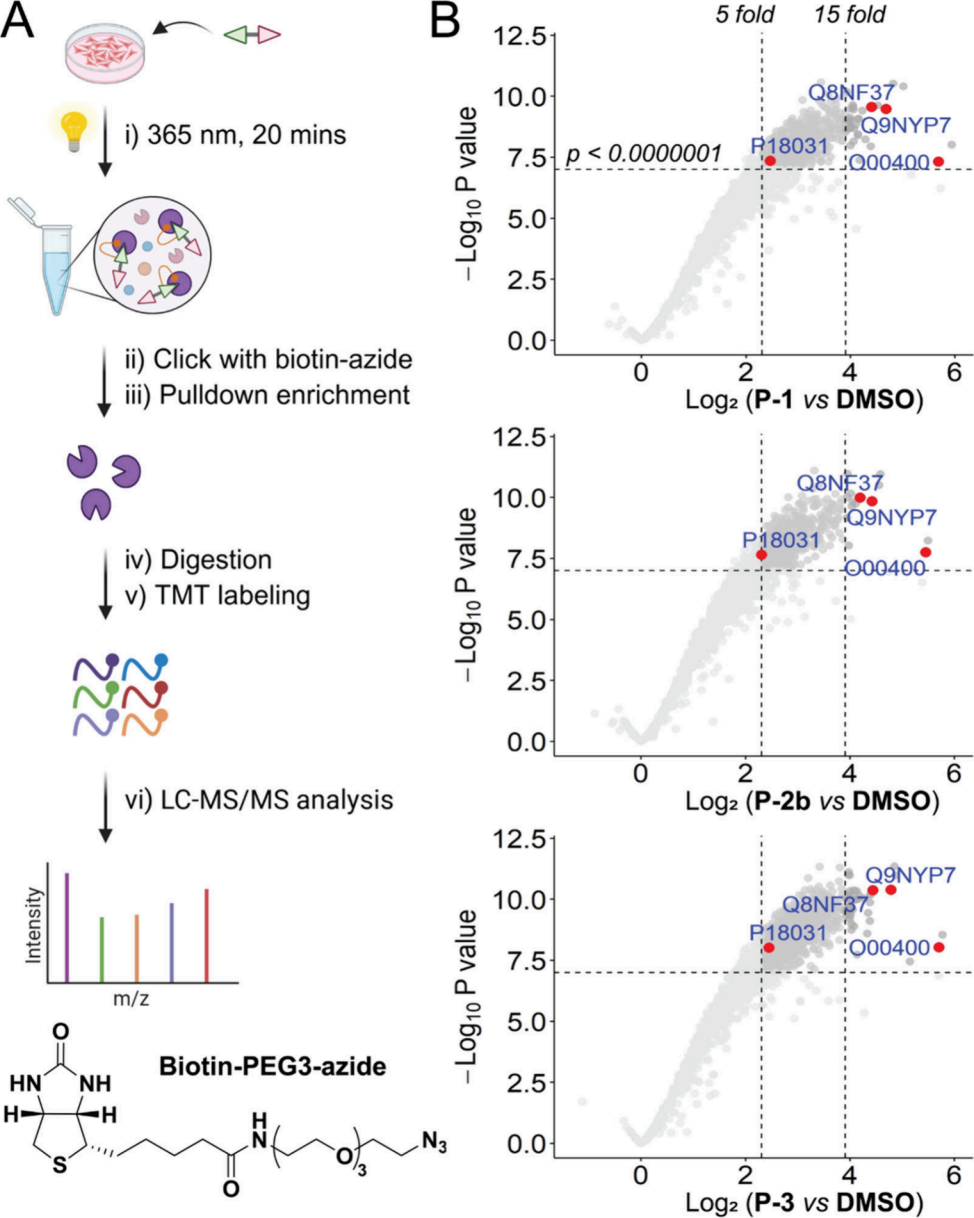
**Target identification by photo-cross-linked labeling coupled with TMT quantitative proteomics.** (A) Schematic diagram illustrating target profiling processes, including photoaffinity labeling, click reaction, enrichment, TMT quantitative proteomic analysis and target prioritization. HCT116 cells were treated with 15 μM of **P-1**, **P-2b** and **P-3** or DMSO for 1 h, followed by lysis, click reaction with biotin azide, acetone precipitation and biotin–neutravidin-based enrichment assay. Created with the assistance of BioRender. (B) Volcano plots of proteins enriched in pull-down. Significantly enriched proteins: fold change >5, *p* < 0.0000001. O00400: SLC33A1; P18031: PTP1B; Q8NF37: LPCAT1; Q9NYP7: ELOVL5.

### PTP1B Is a Direct Target of Type II ICD Inducers, **Pt-NHC** and **Pt-ER**

To prioritize potential ER-resident targets, we selected four proteins, namely SLC33A1 (O00400), LPCAT1 (Q8NF37), ELOVL5 (Q9NYP7) and PTP1B (P18031), for further validation, as highlighted in Figure 4B. In an immunoblotting-based pull-down assay, both SLC33A1 and PTP1B were robustly enriched by all three AfBPs compared to the DMSO control (Supplementary Figure 30), implicating potential interactions between the probes and these two proteins. LPCAT1 and ELOVL5, despite being among the top hits in the proteomics analysis, were not significantly enriched in the pull-down assays.

To validate the direct binding of **Pt-ER** and **Pt-NHC** with SLC33A1 and PTP1B, we performed cellular thermal shift assays (CETSAs) in intact cells and cell lysates following established protocols.^53,54^ CETSA is a label-free technique widely used to assess drug–target engagement in biological systems.^53,55,56^ Both **Pt-ER** and **Pt-NHC** (20 μM) destabilized PTP1B in cell lysates, as evidenced by left-shifted CETSA melting curves and reduced thermal stability with melting temperature shifts (Δ*T*_m_) of −4.7 and −5.5 °C, respectively (Figures 5A and 5B). This destabilization exceeded that of a known PTP1B inhibitor, PTP112 (Δ*T*_m_ = −2.4 °C).^57^ Furthermore, the destabilization of PTP1B by **Pt-NHC** and **Pt-ER** (100 μM) was concentration-dependent, with both compounds nearly eliminating soluble PTP1B in cell lysates at different temperatures (40 °C, Figure 5C; 49 °C, Supplementary Figure 32). In contrast, neither **Pt-ER** nor **Pt-NHC** induced noticeable changes in the thermal stability of SLC33A1 across the 40–58 °C temperature range compared to the DMSO control (Supplementary Figure 31A). The reduction of soluble SLC33A1 in HCT116 cell lysates was observed only when the concentration of Pt compounds was increased to 500 μM (Supplementary Figure 31 B).

**Figure 5:**
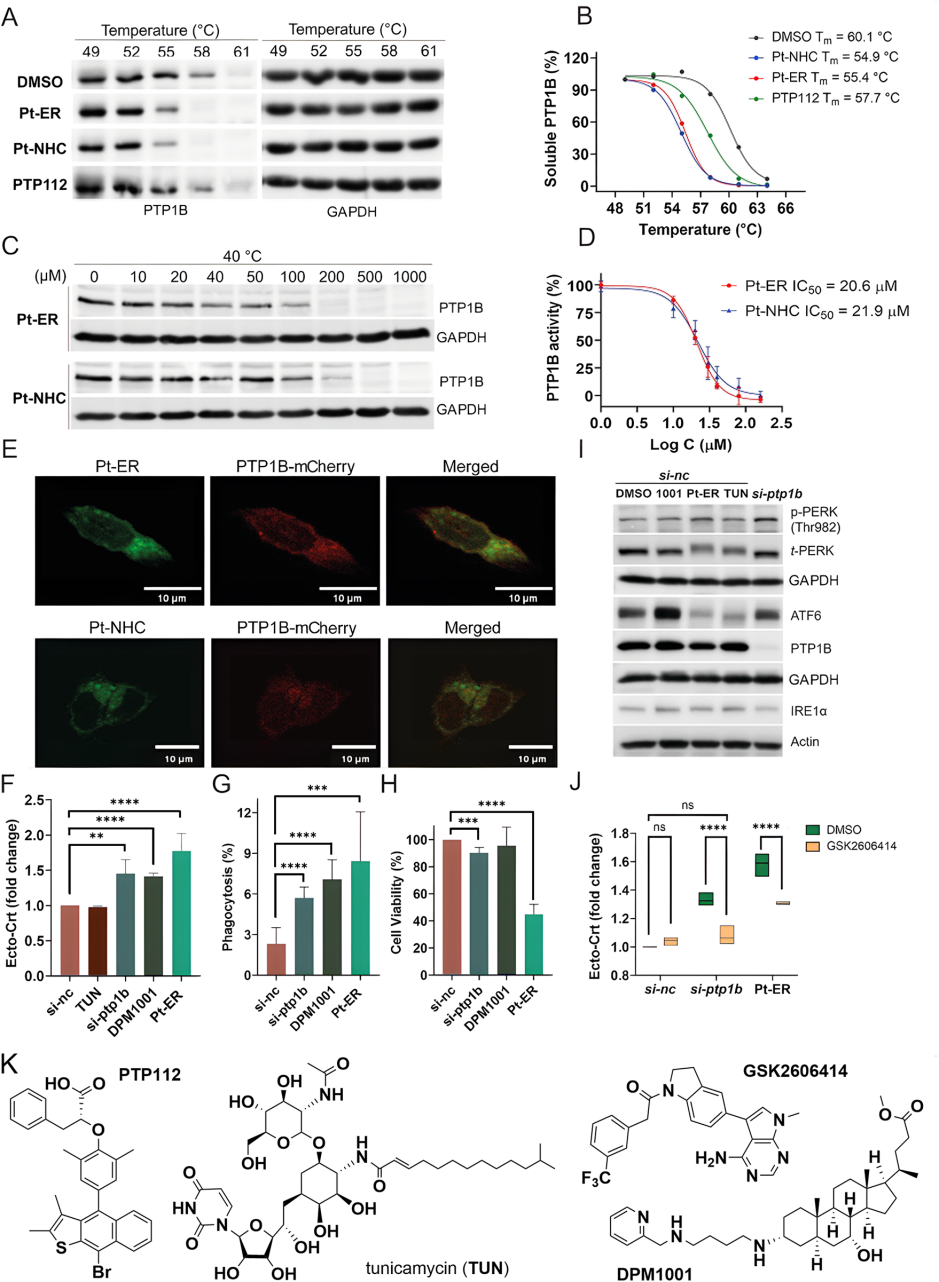
**Validation on the interaction between Pt-ER/Pt-NHC and PTP1B.** (A) Western blot CETSAs for PTP1B in response to **Pt-ER** (20 μM), **Pt-NHC** (20 μM) and PTP112 (5 μM). (B) CETSA melting curves corresponding to Figure 4A. Each data point represents the relative band intensity to 49 °C treatment after normalization. Data are shown as the mean of three repeats. (C) Isothermal dose–response CETSAs for PTP1B at 40 °C in response to increasing concentrations of **Pt-ER** and **Pt-NHC**. Equal amounts of HCT116 cell lysates were incubated with compounds at the indicated concentrations. (D) Dose–response curves of **Pt-NHC** and **Pt-ER** for the inhibition of PTP1B activity determined by *p*-nitrophenyl phosphate assay. Data are shown as a mean of three repeats. (E) Representative confocal microscopy images of the colocalization of PTP1B and organoplatinum compounds. PTP1B-mCherry-overexpressing HCT116 cells were treated with **Pt-ER** (10 μM) or **Pt-NHC** (7.5 μM) for 1 h. Images were taken under 100× objectives. (F) Flow cytometry analysis of ecto-CRT levels on the surface of live HCT116 cells upon treatment with non-specific control siRNA (si-nc) or PTP1B-targeting siRNA (si-ptp1b) (48 h) and indicated compounds (4 h)—TUN: tunicamycin, 10 μg/mL; DPM1001, 20 μM; Pt-ER, 10 μM. Data is shown as a fold change of mean fluorescence intensity normalized to control (si-nc + DMSO). (G) Flow cytometry analysis of phagocytosis of HCT116 cells by U937 monocytes-derived macrophages. U937 macrophages stained with non-toxic cell tracker green dye CMFDA were co-incubated with cell tracker red dye CMPTX-stained HCT116 cells treated with indicated siRNAs for 48 h or indicated compounds for 4 h. (H) Cell viability of HCT116 cells upon treatment with indicated siRNAs (48 h) or indicated compounds (4 h), determined by WST-8 assay. (I) Western blot analysis of UPR pathways in HCT116 cells treated with indicated siRNAs (68 h), followed by DMSO or indicated compounds (4 h). (J) Flow cytometry analysis of ecto-CRT levels on the surface of live siRNA-transfected HCT116 cells pretreated with PERK inhibitor GSK2606414 (1 μM) for 1 h, followed by Pt-ER (10 μM, 4 h) or DMSO. (K) Molecular structures of inhibitors used in the experiments. Mean ± standard deviations were calculated based on at least three independent experiments. Statistical analysis was performed using the unpaired two-tailed Student’s *t* test (F–H) and two-way ANOVA (J); ns: not significant, *p < 0.05, **p < 0.01, ***p < 0.001, ****p < 0.0001.

Given that PTP1B is a protein tyrosine phosphatase, we assessed the impact of both Pt complexes on its enzymatic function by measuring the phosphatase activity using recombinant PTP1B protein in the presence of **Pt-ER** and **Pt-NHC**.^57,58^ The assays demonstrated that both compounds effectively inhibited PTP1B activity *in vitro*, with IC_50_ values of 20.6 ± 1.8 μM for **Pt-ER** and 21.9 ± 2.6 μM for **Pt-NHC** (Figure 5D). For comparison, the reported IC_50_ values for the active site PTP1B inhibitor PTP112 range from 1.6 to 29 μM under varying experimental conditions,^59^ while the allosteric inhibitor DPM1001 exhibited an IC_50_ of 100 nM.^60^ Subsequently, to assess whether Pt complexes and PTP1B localize within the same cellular compartment, we transfected HCT116 cells with mCherry-tagged PTP1B and performed colocalization analysis by fluorescence microscopy, which revealed a marked colocalization between both Pt complexes and PTP1B (Pearson’s correlation coefficients (*r*) of 0.94 for **Pt-ER** and 0.87 for **Pt-NHC**) (Figure 5E). Collectively, these results strongly support direct interactions of the Type II ICD inducers **Pt-ER** and **Pt-NHC** with ER-localized protein PTP1B.

### PTP1B Modulates the Immunogenicity of Tumor Cell Death

To determine the role of PTP1B in CRT translocation during the initiation of ICD, we quantified ecto-CRT levels by flow cytometry in HCT116 cells following PTP1B knockdown or pharmacological inhibition. As expected, transfection of HCT116 cells with non-targeting control siRNA (si-nc) showed unchanged ecto-CRT levels, while PTP1B-specific siRNA transfection (si-ptp1b, knockdown efficiency is shown in Supplementary Figure 33) significantly increased ecto-CRT levels by 1.45-fold (Figure 5F). A comparable 1.42-fold increase was observed following 4-h treatment with the PTP1B inhibitor DPM1001 (20 μM). Notably, Pt-ER (10 μM) induced a stronger 1.77-fold elevation in ecto-CRT levels. Conversely, tunicamycin, an ER stressor that does not inhibit PTP1B, showed no increase in ecto-CRT exposure, consistent with previous reports that tunicamycin alone did not trigger ICD.^61^ Additionally, we knocked down PTP1B in triple negative breast cancer MDA-MB-231 cells (Supplementary Figure 33). We again observed the increase of ecto-CRT exposure, albeit less pronounced than in HCT116 cells, correlating with the lower knockdown efficiency (Supplementary Figure 34A). Similar to HCT116 cells, both DPM1001 and PTP1B knockdown enhanced ecto-CRT levels in MDA-MB-231 cells, though the effects were more modest (∼1.2-fold increase) (Figure 5F and Supplementary Figure 34B).

To further validate the role of PTP1B in ICD, we assessed whether PTP1B suppression enhanced the immunogenicity of dying tumor cells by assessing macrophage-mediated phagocytosis. PTP1B suppression via si-ptp1b, DPM1001 or **Pt-ER** resulted in the increased phagocytosis of HCT116 cells (Figure 5G). Similar to ecto-CRT exposure, the level of phagocytosis in MDA-MB-231 induced by DPM1001 was less pronounced (Supplementary Figure 34C). Comparison of the viability of HCT116 cells following PTP1B knockdown or a 4-h treatment with DPM1001 (20 μM) or **Pt-ER** (10 μM) revealed pronounced differences. While PTP1B silencing and DPM1001 caused only minor reductions in live cells (89.9% and 95.5% live cells for PTP1B knockdown and DPM1001, respectively), **Pt-ER** induced pronounced cell death, leaving only 44.7% live cells (Figure 5H).

Given that PERK is a key ER stress sensor involved in the UPR,^62,63^ we examined the status of the three main ER stress sensors—PERK, IRE1α and ATF6—following PTP1B suppression. In PTP1B-silenced HCT116 cells, phosphorylation of PERK markedly increased, consistent with the reduced expression levels of PTP1B, while total PERK (*t*-PERK), IRE1α and ATF6 remained largely unchanged compared to control siRNA-treated cells (Figure 5I). Treatment of HCT116 cells with DPM1001 (20 μM) induced robust accumulation of ATF6 without markedly altering PERK or IRE1α. Conversely, **Pt-ER** (10 μM) triggered PERK hyperactivation, as indicated by increased p-PERK concomitant with *t*-PERK reduction, and significantly suppressed ATF6. This contrasted with tunicamycin (negative control), which reduced ATF6 expression without affecting PERK.

To further determine whether PTP1B suppression stimulated ICD through PERK activation, we then examined whether PERK inhibition by the known PERK inhibitor GSK2606414 (Figure 5K) would prevent ICD initiation.^64^ Ecto-CRT levels were measured in HCT116 cells treated with control siRNA, PTP1B-targeting siRNA or **Pt-ER**, in the presence or absence of the PERK inhibitor (Figure 5J). While GSK2606414 alone did not alter ecto-CRT levels, it significantly reduced ecto-CRT in both PTP1B-knockdown and **Pt-ER**-treated cells, indicating that PTP1B suppression promoted CRT translocation and ICD initiation via PERK activation.

### PTP1B Plays a Role in Tumor Progression and Immune Regulation

To evaluate the role of PTP1B (*PTPN1*) in cancer, we conducted a bioinformatic analysis of 19,131 samples from 34 cancer types in the The Cancer Genome Atlas (TCGA), Therapeutically Applicable Research to Generate Effective Treatments (TARGET) and Genotype-Tissue Expression Project (GTEx) cohorts. Tumor-versus-normal comparisons were possible for 23 cancer types (Supplementary Figure 36), revealing statistically significant differential expression (*p* < 0.05) in 16 of them (Figure 6A). The expression of *PTPN1* was significantly upregulated in tumor tissues compared to normal tissues in 6 cancer types (Supplementary Figure 36). Conversely, it was significantly downregulated in 10 other cancer types. Our analysis identified a significant increase in *PTPN1* expression in colon adenocarcinoma (COAD) (log2FC = 0.76, p = 6.8 × 10^–10^), consistent with previously published analysis of TCGA or Clinical Proteomic Tumor Analysis Consortium (CPTAC) databases.^65^ Next, we conducted a pan-cancer survival analysis to evaluate the prognostic value of *PTPN1* gene expression across 32 different cancer types (Figure 6B and Supplementary Figure S37). In total, we analyzed 10,496 tumor samples across 32 cancer types. A significant association between *PTPN1* expression and overall survival (*p* < 0.05) was identified in 22 of these cancers. The most significant correlations were observed in kidney renal papillary cell carcinoma (KIRP), sarcoma (SARC), brain lower grade glioma (LGG) and uveal melanoma (UVM). Notably, the prognostic impact of *PTPN1* was cancer-type-specific: high expression was associated with improved survival in SARC and COAD, but with a poorer prognosis in KIRP, LGG, and UVM. Subsequently, we characterized *PTPN1* expression within the tumor immune microenvironment through bioinformatic analysis of a large single-cell RNA-sequencing cohort of colorectal cancer (GSE132465; 63,689 cells from 23 tumors and 10 normal mucosa samples) (Figure 6C and Supplementary Figure 38).^66^ This analysis revealed distinct, cell type-specific expression patterns. Among immune lineages, *PTPN1* was most prevalent in myeloid (37.2%) and B cells (29.9%), with lower frequency in T cells (13.5%). Expression was also detected in non-immune compartments, including epithelial (28.6%), stromal (21.5%) and mast cells (12.3%). Statistical comparison between tumor and normal compartments showed no significant differences in *PTPN1* expression across major immune cell populations. A critical finding was the specific and significant upregulation of *PTPN1* in tumor-infiltrating B cells ( *p* = 0.030), a change not observed in T or myeloid cells, highlighting a potential role for B cell-specific PTP1B signaling in the colorectal cancer microenvironment. To examine whether PTP1B was linked to ER stress signaling, we analyzed expression correlations between *PTPN1* and two central ER stress sensors in primary colon adenocarcinoma samples from TCGA. As shown in Figure 6D, *PTPN1* expression positively correlated with *EIF2AK3* (*ρ* = 0.43, p = 1.3 × 10^–14^) and *ERN1* (*ρ* = 0.47, p = 2.4 × 10^–17^), encoding PERK and IRE1α, respectively. Tumors with higher *PTPN1* expression tended to show elevated levels of both ER stress regulators, pointing to a possible transcriptional link between PTP1B and the unfolded protein response pathway in colorectal cancer.

**Figure 6:**
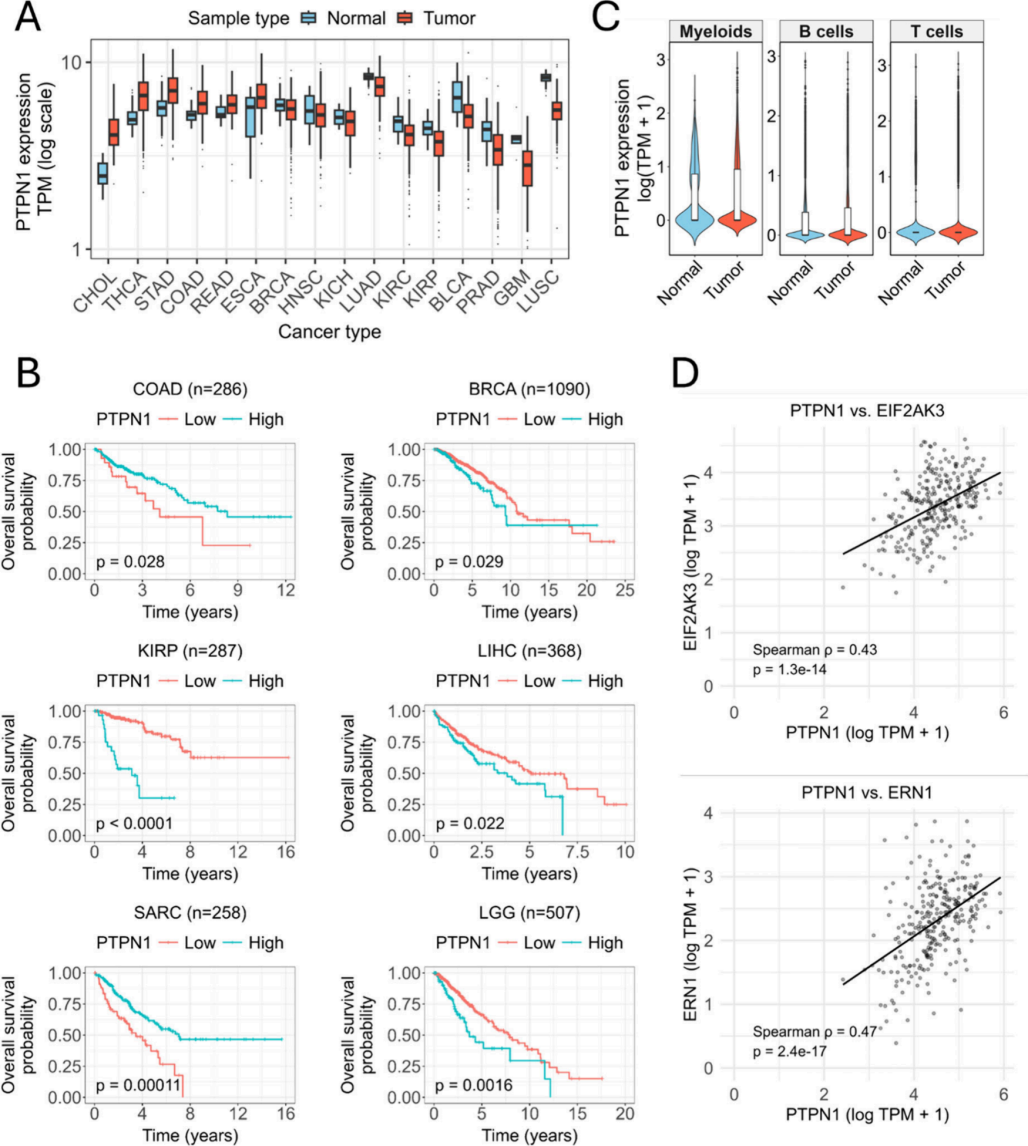
***PTPN1* expression, patient survival and gene correlation analyses across cancer data sets.** (A) Boxplots showing *PTPN1* transcript abundance across tumor and matched normal tissues from TCGA, GTEx and TARGET cohorts. Expression values are represented as log-transformed TPM. Statistical differences between tumor and normal groups were evaluated using two-sided *t* tests. COAD: colon adenocarcinoma; BRCA: breast invasive carcinoma; KIRP: kidney renal papillary cell carcinoma; LIHC: liver hepatocellular carcinoma; SARC: sarcoma; LGG: brain Lower grade glioma. (B) Kaplan–Meier survival curves illustrating overall survival differences between patients with high and low *PTPN1* expression in TCGA cohorts (COAD, BRCA, KIRP, LIHC, SARC, LGG). Patient groups were defined using optimal cutpoints derived from the maximally selected rank statistic. *P*-values were obtained using the log-rank test. (C) Violin plots displaying *PTPN1* expression levels in myeloid, B cell and T cell populations derived from normal and tumor samples of the GSE132465 single-cell RNA-seq colorectal cancer data set. Expression is shown as log­(TPM + 1). (D) Scatter plots show pairwise correlations between *PTPN1* and ER stress-related genes, *EIF2AK3* (upper) and *ERN1* (lower), in primary colon adenocarcinoma samples from the TCGA-COAD cohort. Each point represents one tumor sample. Expression values are log-transformed TPM obtained from the TCGA TARGET GTEx harmonized data set. Solid black lines indicate linear regression fits. Correlation strength was evaluated using Spearman’s rank test, with corresponding ρ and p-values displayed on each plot.

## Discussion

While numerous secondary targets outside the ER have been implicated in Type I ICD, ER-localized molecular targets driving Type II ICD remain largely unidentified. Until now, the ER chaperone BiP (GRP78/HSPA5) has remained the sole ER-resident protein directly engaged by Type II ICD inducers, including Ir­(III)-based complex Ir-NHC and Ru­(III) complex KP1019. Our goal was to identify protein targets mediating the ICD mechanism of two organoplatinum­(II) complexes, **Pt-NHC** and **Pt-ER**, which we established as efficient Type II ICD inducers *in vitro* and subsequently validated *in vivo*. For target deconvolution, we selected the photoaffinity labeling strategy, given its capacity to irreversibly capture transient or low-affinity protein interactions through *in situ* photo-cross-linking, thereby enabling robust identification of physiological binding partners.^67^ To avoid steric interference, where the photoreactive tag could obstruct binding pockets or alter drug conformation, potentially masking region-dependent targets, we designed four different photoaffinity labeled probes by derivatizing **Pt-ER** at distinct structural sites. Probes functionalized at pendant alkyl arms (**P-1**, **P-2a**, **P-2b**) retained **Pt-ER**’s capacity to induce DAMP release, including CRT translocation, HMGB1 release and ATP secretion, confirming effective *in vitro* ICD induction. Notably, the probe modified at the C3 linker bridging the NHC ligands (**P-3**) demonstrated significantly enhanced ICD activity. Functionally validated AfBPs were subjected to TMT-based quantitative proteomics, with three probes representing unique modification scaffolds.

Assuming that high-confidence direct binders of **Pt-ER** would be statistically enriched among proteins shared by all probes, we prioritized 515 significantly enriched proteins. Based on the established ER-centric mechanism of Type II ICD inducers, we further refined this list to 140 ER-resident proteins. Among these candidates, we prioritized three proteins with >15-fold enrichment (SLC33A1, LPCAT1 and ELOVL5) for validation. LPCAT1 and ELOVL5 showed no detectable binding, likely due to the differences in cross-linking efficiencies or labeling reactivities during the target profiling process. Although immunoblotting confirmed SLC33A1 enrichment in **Pt-ER** pull-down, CETSA revealed no significant thermal stability shifts upon Pt treatment, excluding direct binding.

Given the absence of direct engagement with top-enriched ER proteins, we then prioritized lower-enriched (ca. 5-fold) PTP1B protein for validation based on its established role in the ER stress. Specifically, PTP1B was shown to directly interact with PERK, a critical mediator of CRT translocation during ICD,^68,69^ and catalyze the dephosphorylation of tyrosine-phosphorylated PERK.^70^ We validated PTP1B enrichment in pull-down assays through immunoblotting and confirmed direct **Pt-ER**–PTP1B binding through CETSA. Importantly, **Pt-NHC** engaged PTP1B in a similar manner, suggesting conserved PTP1B targeting across these organoplatinum­(II) ICD inducers.

Although we unambiguously identified PTP1B as a direct **Pt-ER** target, its functional contribution to ICD required further validation. Since ecto-CRT serves as a critical “eat-me” signal and dictates the immunogenicity of dying tumor cells,^62,71^ we investigated whether PTP1B played a role in CRT translocation during the initiation of ICD. Genetic depletion of PTP1B in HCT116 cells, as well as pharmacological inhibition using the established PTP1B inhibitor DPM1001, resulted in a significant increase of ecto-CRT surface exposure levels. In parallel, substantial enhancement of phagocytosis was observed upon PTP1B inhibition. Importantly, these functional consequences were not restricted to the HCT116 cell line but were reproduced in triple negative breast cancer MDA-MB-231 cells, indicating that PTP1B-mediated potentiation of immunogenicity likely represents a general mechanism across multiple cancer types.

We observed that DPM1001 triggered concentration-independent immunogenicity with modest viability loss, while the ICD induction by **Pt-ER** correlated with the increase of cytotoxicity. We speculated that DPM1001’s nanomolar irreversible inhibition of PTP1B (reported IC_50_ ≈ 100 nM) might fully saturate the target at low doses, inducing sub-lethal ER stress that triggers concentration-independent ICD without compromising viability. In contrast, **Pt-ER** likely engages other pathways beyond PTP1B inhibition, inducing unresolvable ER stress that drives pronounced cell death. As expected, the subsequent investigation of UPR pathways revealed drastically different effects of PTP1B inhibitors on pro-survival and pro-death UPR arms: DPM1001 selectively amplified the adaptive ATF6 response, while **Pt-ER** activated the PERK axis (mimicking genetic PTP1B inhibition), while additionally suppressing pro-survival ATF6, similar to tunicamycin.

Given that both PTP1B knockdown and **Pt-ER** induced PERK activation, we determined whether this pathway was necessary for ICD induction. GSK2606414-mediated PERK inhibition significantly suppressed ecto-CRT expression in both scenarios, confirming the critical role of PERK for PTP1B inhibitor-induced ICD. However, while PERK blockade fully suppressed ICD in PTP1B-knockdown cells, it only partially reduced ecto-CRT in **Pt-ER**-treated cells, further suggesting that **Pt-ER** engages additional PERK-independent immunogenic pathways beyond PTP1B inhibition.

The ER-resident protein tyrosine phosphatase PTP1B, encoded by *PTPN1*, is an abundant enzyme recognized as a key negative regulator of insulin and leptin signaling.^72^ Beyond metabolic control, PTP1B governs a wider network of oncogenic pathways by dephosphorylating critical receptor tyrosine kinases and protein substrates.^70,73,74^ This positions PTP1B as a significant “accelerator” of tumor progression, promoting proliferation, differentiation and metastasis.^75–77^ Its frequent upregulation in diverse malignancies has motivated the development of small-molecule inhibitors for cancer therapy, with several candidates currently in clinical evaluation (e.g., NCT04777994).^78–80^ In agreement with the literature,^65^ our bioinformatic analysis confirmed significant PTP1B upregulation in colon cancer and other tumor types. Moreover, we demonstrated in various tumor types that the elevated expression was directly correlated with poorer patient survival, thereby providing strong clinical validation for PTP1B as a compelling therapeutic target.

Beyond its established role as an oncoprotein, PTP1B is emerging as a novel intracellular immune checkpoint.^81^ In T cells, its upregulation acts to limit antitumor function, and its inhibition has been shown to potentiate immunity by enhancing the activity of both T cells and dendritic cells.^78,80,81^ Our bioinformatic analysis of publicly available single-cell RNA sequencing data from colorectal cancer patients^66^ revealed a distinct PTP1B expression profile in colorectal tumors: while unaltered in T cells, it was significantly upregulated in tumor-infiltrating B cells. Therefore, targeting PTP1B could enhance B cell-mediated antitumor immunity, potentially leading to more robust and durable immunological memory.

Our analysis of patient samples revealed a direct correlation between *PTPN1* expression and the ER stress sensors PERK and IRE1α. This aligns with the established role of PTP1B in regulating the adaptive UPR by dephosphorylating and inactivating PERK.^70,82^ Consequently, PTP1B inhibition sustains PERK activity during ER stress, leading to enhanced eIF2α phosphorylation.^70,82^ This is critically relevant to ICD, as a productive ICD response is driven by the specific ER stress signature dominated by PERK-eIF2α axis activation.^84^

## Conclusion

Through an integrated chemoproteomics strategy combined with biochemical and cellular assays, we identified and validated PTP1B as a direct intracellular target of the *bona fide* Type II ICD inducer, **Pt-ER**. We demonstrated that **Pt-ER** binds to and inhibits PTP1B at the ER, leading to enhanced surface exposure of CRT and increased phagocytosis of tumor cells which are important hallmarks of ICD. These findings reveal a previously unrecognized role for PTP1B in regulating the immunogenicity of tumor cell death and suggest that targeting PTP1B may offer a promising strategy to enhance the efficacy of cancer chemoimmunotherapy.

## Supplementary Material







## Data Availability

All relevant data supporting the findings of this study are available within the article and Supporting Information. Proteomics data were deposited in ProteomeXchange via the PRIDE database with project accession: PXD065824.

## Notes

Materials and methods; instrumentation; general procedure for synthesis of AfBPs; RP-HPLC analysis; stability spectra in water/ACN 9:1; cell lines and culture conditions; cell viability assay; flow cytometry-based ecto-CRT, intracellular HMGB1 detection and phagocytosis assay; confocal microscopy experiments; Western Blotting experiments; extracellular ATP quantification assay; in situ photoaffinity-based labeling assay; TMT quantitative proteomics analysis; protein knockdown; cellular thermal shift assay; PTP1B enzymatic assay; animal experiments; tumor analysis; statistical analysis; bioinformatic analysis; ^1^H and ^13^C NMR spectra; ESI-MS spectra (PDF)
Supplementary Table 2: protein list identified by TMT quantitative proteomics analysis (XLS)
Supplementary Table 3: list of enriched GO biological processes for overlapping protein targets (XLS)
