# Supplementary material for: Organoplatinum(II) Type II Immunogenic Cell Death Inducers Target Protein Tyrosine Phosphatase 1B to Drive Immunogenicity
Source: J Am Chem Soc. 2025 Dec 23;148(2):2660–72. doi: 10.1021/jacs.5c18904 (PMC12833861; doi:10.1021/jacs.5c18904)
Supplement: Supplementary file 1 [file ja5c18904_si_001.pdf]

Organoplatinum(II) Type II immunogenic cell death inducers target protein tyrosine phosphatase 1B to drive immunogenicity

Jiao Xia Zou,[a] *Pavel A. Ivanov-Rostovtsev*[b,1] *Jemma Arakelyan,*[b,1] *Maria V. Babak,*[b*] *Wee Han Ang*[a,c,*]

[a] Department of Chemistry, National University of Singapore, 4 Science Drive 2, Singapore 117543, Singapore

[b] Drug Discovery Lab, Department of Chemistry, City University of Hong Kong, 83 Tat Chee Avenue, Hong Kong SAR, 999077, People's Republic of China

[c] NUS Graduate School - Integrative Science and Engineering Programme (ISEP), National University of Singapore, 21 Lower Kent Ridge Road. Singapore 119077, Singapore

[1] = equally contributing authors

[*]Corresponding emails: ang.weehan@nus.edu.sg (Wee Han Ang), mbabak@cityu.edu.hk (Maria V. Babak)

Table of contents

Table of contents 2

- Materials and methods 3
  - Reagents, Antibodies and Cell Culture 3
  - Purity of AfBPs on reverse phase-HPLC 4
  - Aqueous stability of probes by UV-vis spectrometer 4
  - Cell viability assay WST-8 (48 h) 4
  - Western blot 4
  - Ecto-CRT detection by flow cytometry 5
  - Intracellular HMGB1 measurement by flow cytometry 5
  - ATP detection assay 5
  - Phagocytosis assay 5
  - *In situ* photoaffinity labelling and pull-down assay 6
  - Quantitative TMT Proteomics Analysis 6
  - Immunoblotting of pull-down samples 7
  - Cellular Thermal Shift Assay (CETSA) 7
  - Enzymatic assay of PTP1B 7
  - Colocalization analysis in confocal laser scanning microscopy 8
  - SiRNA knockdown 8
  - *In vivo* experiments 8
  - Statistical Analysis 9
  - Computational and transcriptomic data analysis 9
  - Synthesis and characterization of Pt-ER and AfBPs 10
- Supplementary Tables 16
- Supplementary figures 17
- References 52

Materials and methods

Reagents, Antibodies and Cell Culture

All chemicals and solvents were used directly without additional purification. All NMR spectra were recorded on a 400 or 500 MHz Bruker UltraShield™ spectrometer at 25 ºC. The residual solvent peaks were used as a reference for NMR spectra ($CDCl_3$: δ 7.26 ppm for ^{1}H-NMR and δ 77.16 ppm for ^{13}C-NMR). The following abbreviations were used: s = singlet, d = doublet, t = triplet, q = quartet, m = multiplet, bs = broad singlet, dd = doublet of doublets. Mass spectrometry performed at the National University of Singapore, the Chemical, Molecular and Materials Analysis Centre (CMMAC).

All synthesized metal complexes were dissolved in DMSO and calibrated by Inductively Coupled Plasma Optical Emission spectroscopy (ICP-OES analysis) before use. Human colon cancer cells HCT116 and triple-negative breast cancer cells MDA-MB-231 were cultured in complete Dulbecco's modified Eagle's medium (DMEM). U937 monocytes were cultured in complete RPMI1640 (supplemented with 10 % FBS and Antibiotic-Antimycotic) at 37 °C in a humidified incubator with 5% CO_2. Cells were authenticated and tested to be mycoplasma free. For all cell-based assays, DMSO concentration was maintained at less than 0.1 %.

Antibodies used in the study: Phospho-eIF2α (Ser51) (D9G8) XP® Rabbit mAb (Cell Signaling Technology, #3398S), eIF2α (D7D3) XP® Rabbit mAb (Cell Signaling Technology, #5324), anti-GAPDH Rabbit antibody (Abcam, # ab9485), Calreticulin (D3E6) XP® Rabbit mAb (Alexa Fluor® 488 Conjugate) (Cell Signaling Technology, #62304S), LPCAT1 (E4V4B) Rabbit mAb (Cell Signaling Technology, #57411S), PTP1B Rabbit mAb (Cell Signaling Technology, #5311S), Alexa Fluor® 488 anti-HMGB1 Antibody (Biolegend, #651410), rabbit anti-SLC33A1 (Thermo Fisher Scientific, PA5-42441), ELOVL5 (B-3) mAb (Santa Cruz, #sc-374138), PHOSPHO-PERK (THR982, Thermo Fisher, # PA540294), IRE1α (14C10) Rabbit mAb (Cell Signaling Technology, #3294T), ATF-6 (D4Z8V) Rabbit mAb (Cell Signaling Technology, #65880), PERK (C33E10) Rabbit mAb (Cell Signaling Technology, #3192).

Inhibitors, reagents, siRNAs, protein and plasmid used were the following: Ertiprotafib (PTP112, MedChemExpress, #HY-19383), Tunicamycin (MedChemExpress, #HY-A0098), DPM1001 (MedChemExpress, # HY-121515), 4-Nitrophenyl phosphate (MedChemExpress, # HY-116022), G-418 (MedChemExpress, # HY-17561), GSK2656157 (MedChemExpress, HY-13820), GSK2606414 (MedChemExpress, HY-18072); Lipofectamine™ RNAiMAX Transfection Reagent (invitrogen, 13778030), HiPerFect Transfection reagent (QIAGEN, #301704); control siRNA (Santa Cruz, #sc-37007), PTP1B siRNA (Santa Cruz, #sc-36328); recombinant human PTP1B (Sino Biological, #10304-H07E); PTP1B-mCherry plasmid (pPTP1BD181A-mCherry, Addgene, #40270).

Purity of AfBPs on reverse phase-HPLC

Probes from DMSO stock solutions were dissolved in 10 % acetonitrile (ACN) in deionized (DI) water and injected into a Shimadzu HPLC (Agilent C18 eclipse reversed-phase analytical column (5 µm, 4.6 x 150 mm); mobile phase: H_2O + 0.1 % formic acid (solvent A) and ACN + 0.1 % formic acid (solvent B) at a flow rate of 1 mL/min; percentage of solvent B changed from 40 % - 90 % (v/v) at rate of 1 %/min; detector λ = 254 nm/ 280 nm). Peak areas at both 254 nm and 280 nm were calculated and are indicative of purity (peaks prior to 3 min of the chromatogram were not included as DMSO appears).

Aqueous stability of probes by UV-vis spectrometer

Probes from DMSO stock solutions were dissolved in DI water (containing 10 % ACN, v/v) to a final concentration at 20 µM. UV-vis absorbance at 200 nm – 800 nm was detected every 1 h for 24 h and plotted in GraphPad Prism 10.0.

Cell viability assay WST-8 (48 h)

In a 96 well plate, $1x10^4$ HCT116 cells were seeded and stabilized for 24 h, followed by treatment with respective compound solutions at a series of serial diluted concentrations in complete DMEM for 48 h. Cell culture medium were then replaced with WST-8 working reagents comprised of WST-8 (0.3 mg/mL, 0.5 mM) and 1-methoxy PMS (0.02 mM) in complete DMEM medium, followed by 1 h incubation at 37 °C, 5 % CO_2. Absorbances were measured at 460 nm by BioTek Synergy H1 multimode microplate reader. IC_{50} and LC_{50} were calculated using Graphpad Prism 10.0.

Western blot

HCT116 cells were treated with respective compound solutions at indicated concentrations for indicated duration, and then harvested and lysed by RIPA lysis buffer (50 mM Tris-HCl pH 8.0, 1% IGEPAL CA-630, 0.1 % sodium deoxycholate, 0.1 % sodium dodecyl sulfate, 150 mM sodium chloride, 1 x Protease inhibitor cocktail). Protein concentration in cell lysates were quantified using Pierce™ Rapid Gold BCA Protein Assay Kit. Equal quantities of protein (20-30 µg) were heated at 95 °C for 10 min and then resolved on SDS-PAGE and transferred to 0.22 µm PVDF membrane. The membrane was blocked with 5 % BSA in TBS-T (50 mM Tris pH 7.4, 150 mM NaCl, 0.05 % Tween 20), probed with corresponding primary antibody and secondary antibody, and finally visualized using Amersham ECL Prime Western Blotting Detection Reagent.

Ecto-CRT detection by flow cytometry

ICD biomarkers detection was adapted from previously described methods.[1-2] 5 × 10^5 HCT116 cells were seeded onto a 6 well plate and stabilized for 24 h, followed by treatment with respective compound solutions at indicated concentration. After 4 h incubation, cells are harvested and blocked with 5 % BSA in HBSS on ice for 30 mins, followed by incubation with rabbit IgG anti-CRT Alexafluor 488 mAb (1:100) in 1 % BSA in HBSS solution on ice for 2 h. Cells were stained with PI to gate out dead cells and subjected to flow cytometry analysis. At least 10 K cells were analyzed for each sample.

Intracellular HMGB1 measurement by flow cytometry

5 × 10^5 HCT116 cells were seeded onto a 6 well plate and stabilized for 24 h, followed by treatment with respective compound solutions at indicated concentration. After 24 h incubation, cells were fixed using 4 % paraformaldehyde (PFA) solution, permeabilized with 0.1 % triton-X100 PBS solution for 10 mins and blocked with 5 % BSA in PBS for 10 min on ice before incubation with anti-HMGB1 Alexafluor 488 antibody (1:100) in 1 % BSA solution at 4 ºC overnight. At least 10 K cells were analyzed by flow cytometry.

ATP detection assay

5 × 10^5 HCT116 cells were seeded onto a 6 well plate and stabilized for 24 h, followed by treatment with respective compound solutions at indicated concentration for indicated duration. Supernatants were then collected, and their ATP concentrations were measured by following the protocol of the ATP detection kit (Molecular Probes™ ATP Determination Kit, A22066 from Thermo Fisher).

Phagocytosis assay

Phagocytosis was determined following previous reports.[3-4] 4 x 10^5 U937 monocytes were seeded into 6 well plate and grown in the presence of by 500 ng/mL PMA (phorbol 12-myristate 13-acetate) in complete RPMI 1640 media for 36 h allowing for differentiation. Differentiated macrophages were attached to the bottom of culture plate with around 1 x 10^5 cells per well. Macrophages were then stained with 1 μM cell tracker green dye CMFDA for 25 min. Meanwhile, 5 × 10^5 HCT116 cells were seeded onto a 6 well plate and stabilized for 24 h, followed by staining with 1 μM cell tracker red CMTPX dye for 20 min and treatment with respective compound solutions at indicated concentration for 4 h. After treatment, 1 x 10^5 cancer cells were then cocultured with macrophages for 4 h at 37 ºC, 5 % CO_2. Subsequently, cells were harvested and subjected to flow cytometry analysis. At least 10 K macrophages were analyzed.

In situ photoaffinity labelling and pull-down assay

Photoaffinity-based target profiling was adapted from previously described methods.[5-8] 5 × 10^5 HCT116 cells were seeded onto a 6 well plate and stabilized for 24 h, followed by treatment with 15 µM respective probe solutions for 1 h. After irradiation at 365 nm for 20 min, cells were harvested and lysed in lysis buffer (1 % NP-40, 0.2 % SDS in 20 mM N-(2-hydroxyethyl)piperazine-N′-ethane sulfonic acid (HEPES) pH 7.5, 1x Protease inhibitor cocktail) on ice for 60 min. Cell lysates at a concentration of 2 mg/mL were subjected to Click reaction by adding a click reaction mixture (100 µM biotin-PEG-azide, 1 mM $CuSO_4$, 100 µM Tris(3-hydroxypropyltriazolylmethyl)amine TPHA; 1 mM Tris(2-carboxyethyl)phosphine hydrochloride TCEP) at room temperature for 1.5 h. Proteins in cell lysates were precipitated out using cold acetone after overnight incubation at - 20 °C. Precipitated protein pellets were then collected at 13,000 rpm, 30 min, washed using cold methanol twice and dried in air. Dry pellets were solubilized in 1× PBS with 4 % SDS, 20 mM EDTA, and 10 % glycerol by vortex and heating. After being diluted to give a final concentration of SDS as 0.5 %, the protein solution was incubated with High capacity neutravidin agarose beads (Thermo Fisher Scientific, 29202) while rotating for 1.5 h at room temperature. The beads were washed stepwise with 1 × PBS with 0.2 % SDS, 6 M urea in PBS twice, 1x PBS three times. After that, beads were transferred to a new clean microtube and washed three times again with 1 × PBS. Dry beads were then submitted to the functional proteomics laboratory (under SingMass unit) at Agency for Science, Technology and Research (A*STAR), in Singapore.

Quantitative TMT Proteomics Analysis

In the functional proteomics laboratory, samples were analysed using Easy nLC (Thermo Fisher Scientific) equipped with PepMapTM RSLC C18 column (Thermo Fisher Scientific, 75 µm x 50 cm), coupled to an Orbitrap Fusion Lumos mass spectrometer (Thermo Fisher Scientific) using data-dependent mode, fitted with a nano spray ion source. Peptides were separated using a 2-30 % (v/v) acetonitrile gradient over 70 min, increasing to 80 % over the next 10 min, and finally to 95 % over 10 min, running at a constant flow rate of 300 nL/min.

The following parameters were set for MS data acquisition in positive ion mode with 3 s cycle time: automatic gain control (AGC) set as custom normalized AGC% at 200 with orbitrap resolution at 60,000 ranging from m/z 350 to 1550. Dynamic exclusion for precursors selected for fragmentation was set to 45 s. The maximum injection time is set at 50 ms. For fragmentation, MS2 isolation window was set to 1.0 m/z of the selected precursor masses and higher-energy collision dissociation (HCD) was activated at 38 % normalized collision energy. Fragment signals (MS2) were analysed by orbitrap analyser set to resolution of 50,000 with AGC target customed at 200 % and maximum injection time (IT) at 80 ms.

Database search was performed with Proteome Discoverer™ (version 3.1, Thermo Fisher Scientific) against human primary protein sequences retrieved from uniprot with 42289 sequences

and 24370425 residues. Reporter ion integration was carried out using summed abundances with 20 ppm tolerance and most confident centroid was set as integration method with FTMS at MS2 order. The precursor mass tolerance was set at 10 ppm and its fragment mass tolerance was at 0.06 Da (MS2 mode) with three missed cleavages. Fixed modifications of carbamidomethyl and TMTpro (peptide N-terminus, K residues) were selected. For peptide scoring, percolator was employed with an identification threshold of false discovery rate (FDR) of 1 %. Only proteins that were identified in 3 sample replicates, with more than one unique peptide were subjected to subsequent statistical analysis. Non-normalized abundance values of proteins from Proteome Discoverer were log2 transformed before further analysis. The statistical significance of differences between groups was assessed using Limma package with moderated t-statistics.

Immunoblotting of pull-down samples

After pull-down, beads samples were heated in 2x Laemmli buffer with 10 % β-mercaptoethanol at 60 ºC for 30 min to release the captured biotinylated proteins. Eluted proteins were resolved on SDS-PAGE and then transferred to 0.22 μm PVDF membrane, followed by blocking, incubation with corresponding primary and secondary antibodies and visualization by Amersham ECL Prime Western Blotting Detection Reagent.

Cellular Thermal Shift Assay (CETSA)

The assay was conducted based on previously reported methods.[9-11] Equal amount of HCT116 cells (2 x 10^6 cells) or cell lysates (2 mg/mL) were incubated with vehicle (DMSO) or respective compounds at indicated concentrations for 1 h. Next, samples were subjected to heat treatment at indicated temperatures for 3 min using Applied Biosystems ProFlex PCR System with 6 different zones, followed by 3-minute cooling down at room temperature. Soluble fractions were obtained by centrifugation at 17,000 x g for 40 min at 4 ºC and then subjected to SDS-PAGE and immunoblotting following the general protocol as previously stated.

Enzymatic assay of PTP1B

The determination of PTP1B activity was adapted from a previously described method.[12] The assay was performed in 96 well plate with a total volume of 100 μL per well. Each well contains 2 mM substrate pNPP, 40 ng recombinant human PTP1B (Sino Biological, 10304-H07E) in buffer solution (25 mM Tris-HCl pH 7.0, 1 mM EDTA), and DMSO or respective complexes at indicated concentrations. DMSO was constant and maintained at 2 %. After incubation at room temperature for 60 min, the absorbance of hydrolysis product NPP was measured at 405 nm using a plate reader (BioTek). Dose-response curves were plotted and IC_{50} were calculated using Graphpad Prism 10.0.

Colocalization analysis in confocal laser scanning microscopy

6 x 10^5 HCT116 cells on a T25 cell culture flask were transfected with 5 μg plasmid (pPTP1BD181A-mCherry, Addgene, 40270) using Lipofectamine™ RNAiMAX Transfection Reagent (invitrogen, 13778030) following the manufacturer's manual. After 12 h incubation allowing for protein expression, 500 μg/mL G418 was added for selection. 1 x 10^5 cells were then seeded onto coverslips and grown in the presence of G418 for 48 hrs, followed by treatment with platinum complexes at indicated concentrations for 60 min. Treated cells were washed, fixed with 4 % paraformaldehyde, and mounted to microscopy slides by applying mountant (ProLong™ Glass Antifade Mountant, Thermo Fisher Scientific, P36980) overnight. Cells were analyzed with confocal laser scanning microscopy Olympus FV3000 using 100 x oil objective lens. Pt-NHC complexes were detected under Ex/Em 340/510 nm,[13] and PTP1B was detected under Ex/Em 587/610 nm. Images were processed using ImageJ software.

SiRNA knockdown

HCT116 cells were transfected with PTP1B-targeting siRNA using transfection reagent following the manufacturers' manuals. After 48-72 h, cells were treated with vehicle (DMSO) or respective compounds at indicated concentration. Afterwards, cells were subjected to microscopy imaging, UPR detection, ecto-CRT, phagocytosis or cell viability determination following the protocols previously described.

***In vivo* experiments**

All *in vivo* procedures were performed in accordance with the institutional and local ethics committee guidelines of the HKSAR Department of Health. Female BALB/c mice, aged 6-8 weeks, were housed under specific pathogen-free conditions. A total of twenty mice were randomly divided into four experimental groups, each containing five mice. The groups were designated as follows: Group 1 received a subcutaneous injection of 100 μL phosphate-buffered saline (PBS) into the shaved left flank. Group 2 was vaccinated subcutaneously with a suspension of 3×105 CT26 cells that had been pre-treated with Doxorubicin, a known ICD inducer. Groups 3 and 4 were vaccinated subcutaneously with 3×105 CT26 cells that had been pre-treated with the experimental compounds Pt-ER and Pt-NHC, respectively. Prior to injection, all mice were anesthetized using 2.5% isoflurane.

One week following the prophylactic vaccination, all mice were challenged with a subcutaneous injection of 3×10^5 living, untreated CT26 cells into the shaved right flank. Subsequent to the tumor challenge, mice were monitored every two days for general health and for the appearance of palpable tumors. Body weights were recorded every second day throughout the entire experiment to monitor for systemic toxicity. The primary endpoint for the study was tumor-free survival,

which was plotted on a Kaplan-Meier curve. All mice remained bright, alert, and responsive for the duration of the observation period.

Statistical Analysis

Quantitative data were analyzed using Graph Prism 10.0 (GraphPad Software, La Jolla, CA) and presented as mean ± SD (standard deviation). Statistical significance of differences was determined using unpaired two-sided Student's t-test or two-way ANNOVA. A *p*-value less than 0.05 was considered statistically significant (*$p < 0.05$, **$p < 0.01$ and ***$p < 0.001$).

Computational and transcriptomic data analysis

Transcriptomic datasets were obtained from publicly available repositories. Bulk RNA-seq data (TPM) and associated metadata were retrieved from the UCSC Toil harmonized compendium integrating TCGA, GTEx, and TARGET cohorts. Single-cell RNA-seq data for colorectal cancer were obtained from GEO accession GSE132465. When applicable, only "Primary Tumor" and "Solid Tissue Normal" samples were included, and cancer type abbreviations were standardized according to TCGA nomenclature. Expression data were handled as transcripts per million (TPM) and analyzed after log transformation. Comparative analyses of *PTPN1* expression between tumor and normal tissues, as well as survival-related evaluations, were performed using harmonized TCGA datasets. Patient groups were defined according to expression-based thresholds.

Single-cell data were analyzed using the provided annotations, with immune cell populations (myeloid, B, and T cells) examined separately. Expression relationships, including those between *PTPN1* and *HSPA5*, were summarized using correlation-based approaches. Associations between PTPN1 and ER stress–related genes (*EIF2AK3*, *ERN1*) were assessed in TCGA colon adenocarcinoma (COAD) primary tumor samples. Analyses followed standard workflows for transcriptomic data processing and visualization.

Synthesis and characterization of Pt-ER and AfBPs

A general procedure for synthesizing Pt-carbene(NHC) complexes

The scaffolds of Pt-NHC complexes were synthesized based on the previous reported methods (Scheme 1).[1, 13]

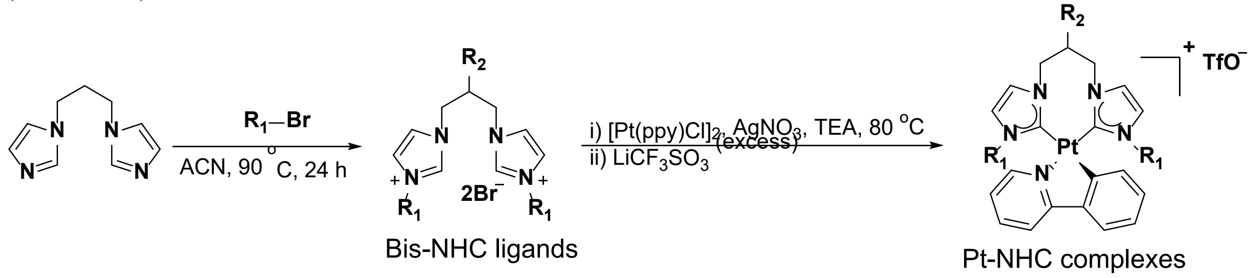

Scheme S1. General synthesis of Pt-NHC complexes.

Synthesis of Pt-ER

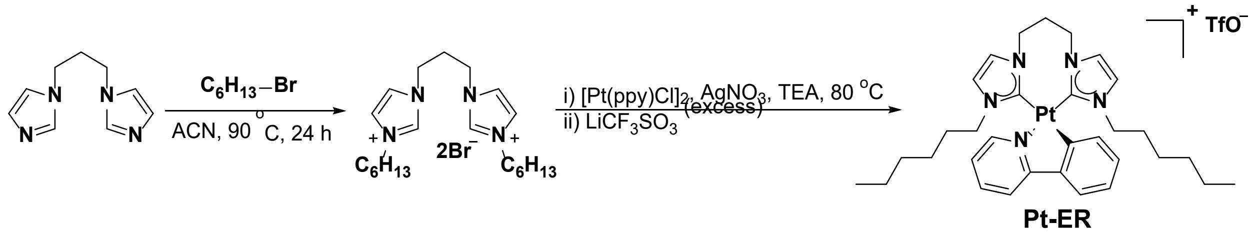

Scheme S2. Synthesis of Pt-ER.

Pt-ER was synthesized as previously reported (Scheme S2). First, bis-NHC ligand was prepared by reacting 1,3-di(1H-imidazol-1-yl)propane (1 mmol equiv.) with 1-bromohexane (2.2 mmol equiv.) in acetonitrile (ACN) at 90 °C for 24 h. The ligand product was purified by column chromatography on silica gel using dichloromethane (DCM) and methanol (MeOH) 10:1 to 5:1 as eluent. Yield: 52 %. ^{1}H NMR (400 MHz, $CDCl_3$) δ 9.95 (s, 2H), 8.10 (t, J = 1.8 Hz, 2H), 7.31 (t, J = 1.8 Hz, 2H), 4.56 (t, J = 7.4 Hz, 4H), 4.14 (t, J = 7.5 Hz, 4H), 2.70 (m, 2H), 1.82 – 1.71 (m, 4H), 1.24 – 1.08 (m, 12H), 0.77 – 0.64 (m, 6H). ^{13}C NMR (126 MHz, $CDCl_3$) δ 136.16, 123.46, 121.66, 49.99, 46.46, 30.84, 30.79, 29.80, 25.67, 22.11, 13.69.

Next, the obtained ligand (1 mmol equiv.) was then used to react with $[Pt(ppy)Cl]_2$ (1 mmol equiv.) in the presence of $AgNO_3$ (3 mmol equiv.), excess triethylamine (TEA, 46 mmol equiv.) were dissolved in ACN and refluxed for 24 h at 80 ºC. The mixture was then cooled to room temperature, filtered and excess $LiCF_3SO_3$ (10 mmol equiv.) was added to replace the counterion. After being stirred for 1 h at room temperature, solvents were removed by evaporation under reduced pressure. The resultant residue was dissolved in water and extracted with DCM. The product was then

purified by column chromatography on silica gel using 20:1 to 10:1 DCM: MeOH as eluent. Yield: 34.1 %. **Pt-ER**: ^{1}H NMR (500 MHz, $CDCl_3$) δ 7.95 (m, 2H), 7.86 (dd, J = 8.8, 1.6 Hz, 1H), 7.65 (d, J = 6.6 Hz, 1H), 7.24 (d, J = 2.1 Hz, 1H), 7.17 – 7.11 (m, 3H), 7.11 – 7.00 (m, 3H), 6.65 (dd, J = 7.3, 1.4 Hz, 1H), 4.82 (m, 2H), 4.43 – 4.31 (m, 3H), 4.24 (dd, J = 14.4, 6.2 Hz, 1H), 4.01 (m, 2H), 2.44 – 2.33 (m, 1H), 2.05 (dt, J = 16.3, 10.8 Hz, 1H), 1.84 – 1.65 (m, 4H), 1.30 – 1.17 (m, 12H), 0.80 (m, 6H). ^{13}C NMR (101 MHz, $CDCl_3$) δ 178.46, 167.95, 159.33, 155.65, 151.44, 146.96, 139.73, 137.15, 130.84, 124.65, 124.10, 123.68, 123.49, 122.70, 120.57, 119.79, 119.68, 52.39, 52.33, 50.89, 50.43, 32.43, 31.28, 30.52, 29.74, 26.54, 26.52, 22.40, 13.93. ESI-MS: m/z 693.64 $[M\text{-}OTf\text{-}2H]^+$.

Synthesis of P-1

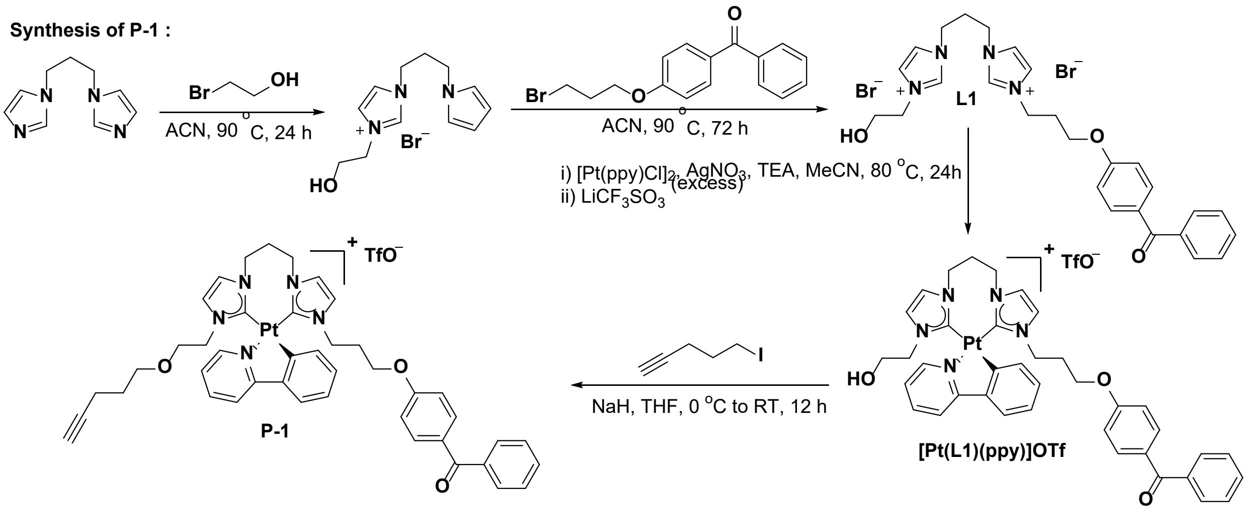

Scheme S3. Synthesis of AfBP P-1.

P-1 was synthesized following the synthetic route shown in Scheme S3. Briefly, the bis-NHC ligand, **L1**, was prepared by installing two functionalized arm chains step by step following the general procedure described above. Following the general synthetic procedure of Pt-NHC complexes described above, **[Pt(L1)(ppy)]OTf** was obtained after column chromatography on silica gel. ^{1}H NMR (500 MHz, $CDCl_3$) δ 8.15 – 7.91 (m, 1H), 7.88 – 7.76 (m, 2H), 7.71 – 7.65 (m, 4H), 7.63 – 7.58 (m, 1H), 7.56 – 7.50 (m, 1H), 7.47 – 7.41 (m, 2H), 7.37 (dd, J = 18.6, 2.0 Hz, 1H), 7.15 – 6.95 (m, 6H), 6.82 – 6.73 (m, 2H), 6.64 (m, 1H), 4.92 – 4.75 (m, 2H), 4.72 – 4.61 (m, 1H), 4.49 – 4.33 (m, 2H), 4.32 – 4.14 (m, 3H), 4.10 – 4.02 (m, 2H), 3.87 (m, 2H), 2.36 – 2.22 (m, 3H), 2.04 – 1.95 (m, 1H). ESI-MS: m/z 807.56 $[M\text{-}OTf\text{-}2H]^+$.

Next, to a solution of [Pt(L1)(ppy)]OTf (1 mmol equiv.) in dry THF at 0 °C, sodium hydride (3 mmol equiv.) was added slowly at inert atmosphere. After 30 min, 5-iodopent-1-yne (3 mmol equiv.) was dropwise added, and the reaction mixture was stirred for overnight. The reaction was quenched by water and solvents were removed by evaporation under reduced pressure. The resultant residue was dissolved in water and extracted with DCM. The product was then purified

by column chromatography on silica gel using 8:1 to 4:1 DCM: acetone as eluent. Yield: 42.2 %. **P-1**: ^{1}H NMR (500 MHz, CDCl3) δ 8.10 – 7.96 (m, 1H), 7.94 – 7.80 (m, 2H), 7.74 – 7.70 (m, 3H), 7.67 – 7.61 (m, 1H), 7.56 (m, 1H), 7.51 – 7.43 (m, 2H), 7.38 – 6.95 (m, 8H), 6.78 (m, 2H), 6.71 – 6.57 (m, 1H), 4.95 – 4.77 (m, 2H), 4.70 (m, 2H), 4.44 – 4.20 (m, 4H), 4.17 – 4.03 (m, 2H), 3.83 – 3.61 (m, 2H), 3.57 – 3.43 (m, 2H), 2.44 – 2.26 (m, 3H), 2.24 – 2.03 (m, 3H), 1.93 (m, 1H), 1.72 (m, 2H). ^{13}C NMR (126 MHz, CDCl3) δ 195.57, 167.92, 162.06, 151.91, 147.03, 139.64, 138.21, 137.07, 132.62, 132.13, 130.99, 130.43, 129.81, 128.35, 124.76, 124.19, 123.68, 123.20, 122.61, 121.24, 120.62, 119.61, 114.05, 83.61, 69.73, 69.11, 68.84, 65.03, 52.32, 50.38, 47.92, 41.07, 32.47, 29.39, 28.28, 15.19. ESI-MS: m/z 873.48 $[M\text{-}OTf\text{-}2H]^{+}$.

Synthesis of P-2a and P-2b

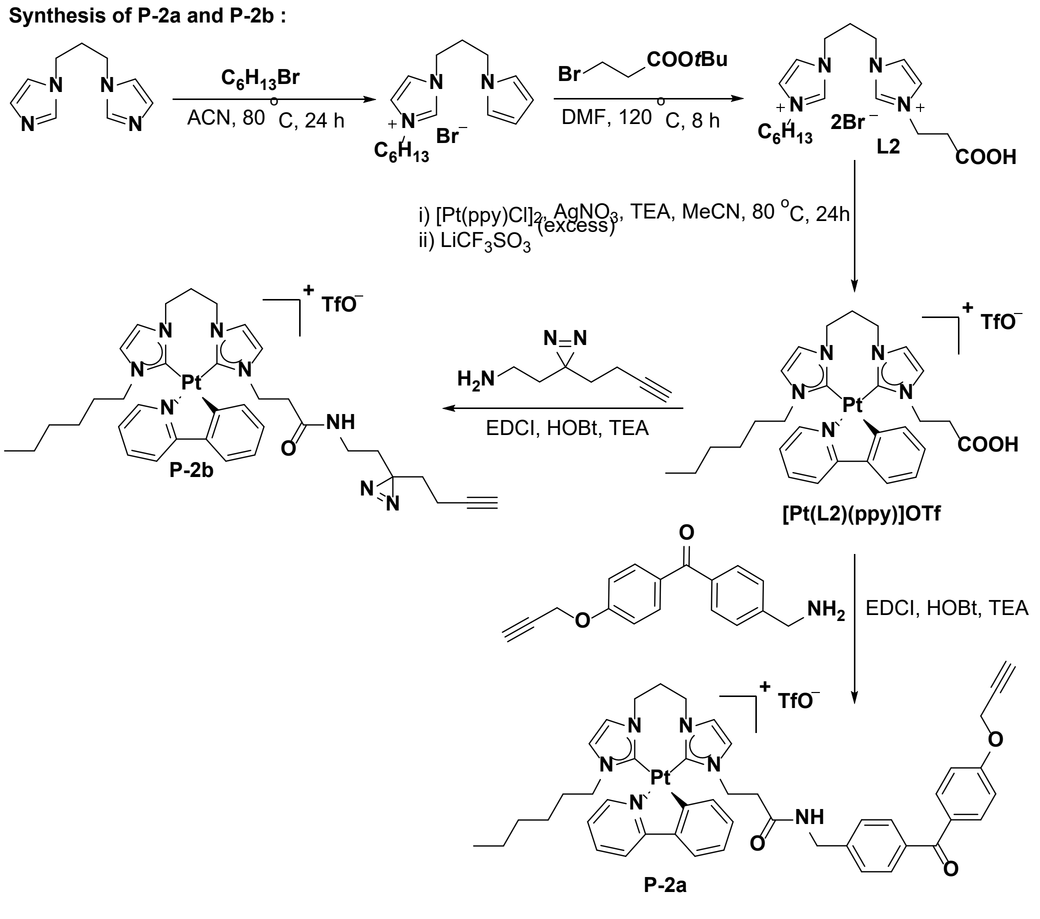

Scheme S4. Synthesis of AfBPs P-2a and P-2b.

P-2a and 2b were synthesized following the synthetic route shown in Scheme S4. Briefly, the bis-NHC ligand, L2, was prepared by installing two functionalized arm chains step by step following the general procedure described above. Following the general synthetic procedure of Pt-NHC complexes described above, [Pt(L2)(ppy)]OTf was obtained after column chromatography on silica gel. ^{1}H NMR (500 MHz, CDCl3) δ 8.03 (d, J = 4.0 Hz, 1H), 7.89 (m, 1H), 7.81 (d, J = 8.1

Hz, 1H), 7.64 (d, J = 7.7 Hz, 1H), 7.29 (s, 1H), 7.23 – 7.10 (m, 3H), 7.09 – 6.99 (m, 3H), 6.66 (dd, J = 7.3, 1.3 Hz, 1H), 5.90 (s, 1H), 4.83 (m, 2H), 4.67 (m, 1H), 4.51 – 4.40 (m, 2H), 4.23 (m, 2H), 4.03 – 3.92 (m, 1H), 2.77 (m, 2H), 2.34 (m, 1H), 2.02 (m, 1H), 1.74 (m, 2H), 1.28 – 1.22 (m, 2H), 1.17 (m, 4H), 0.80 – 0.73 (m, 3H). ^{13}C NMR (126 MHz, $CDCl_3$) δ 178.78, 173.87, 167.68, 159.24, 156.04, 152.00, 147.16, 139.72, 137.27, 130.72, 124.69, 124.24, 124.08, 123.02, 122.20, 121.51, 120.08, 119.57, 52.37, 52.21, 50.97, 46.38, 35.92, 32.45, 31.34, 29.73, 26.41, 22.40, 14.01. ESI-MS: m/z 681.59 $[M-OTf-2H]^+$.

Next, to a solution of [Pt(L2)(ppy)]OTf (1 mmol equiv.) in dry DCM, 1-hydroxybenzotriazole hydrate (HOBt, 2 mmol equiv.), 1-ethyl-3-(3-dimethylaminopropyl)carbodiimide (3 mmol equiv.) and triethylamine (TEA, 5 mmol equiv.) was added. After being stirred for overnight, the reaction mixture was dissolved in water and extracted with DCM. The product was then purified by column chromatography on silica gel using 8:1 to 4:1 DCM: acetone as eluent. **P-2a**: yield: 37 %. ^{1}H NMR (400 MHz, $CDCl_3$) δ 8.11 (t, J = 6.1 Hz, 1H), 8.02 (dd, J = 5.5, 1.6 Hz, 1H), 7.93 (m, 1H), 7.86 (d, J = 8.2 Hz, 1H), 7.81 – 7.76 (m, 2H), 7.67 – 7.62 (m, 3H), 7.39 (m, 2H), 7.24 (m, 2H), 7.15 (m, 1H), 7.11 – 7.00 (m, 5H), 6.90 (d, J = 2.1 Hz, 1H), 6.60 (dd, J = 7.2, 1.4 Hz, 1H), 4.87 (m, 2H), 4.78 – 4.70 (m, 3H), 4.60 (m, 1H), 4.45 (m, 3H), 4.31 – 4.19 (m, 2H), 4.14 (m, 1H), 2.75 (t, J = 6.7 Hz, 2H), 2.56 (t, J = 2.4 Hz, 1H), 2.32 (m, 1H), 2.04 (m, 1H), 1.87 – 1.74 (m, 2H), 1.27 – 1.23 (m, 2H), 1.16 (m, 4H), 0.80 – 0.72 (m, 3H). ^{13}C NMR (126 MHz, $CDCl_3$) δ 195.50, 178.46, 170.10, 168.35, 161.03, 159.28, 155.82, 151.51, 146.81, 143.51, 139.64, 136.90, 136.72, 132.53, 131.39, 131.21, 130.06, 127.80, 124.86, 124.29, 123.20, 122.44, 122.19, 121.72, 121.26, 119.78, 114.51, 77.95, 76.30, 56.01, 52.30, 50.50, 46.78, 43.23, 36.12, 32.41, 31.40, 30.77, 26.41, 22.42, 14.04. ESI-MS: m/z 928.54 $[M-OTf-2H]^+$.

P-2b: yield: 39 %. ^{1}H NMR (500 MHz, $CDCl_3$) δ 8.10 (dd, J = 5.6, 1.5 Hz, 1H), 7.93 (m, 1H), 7.84 (d, J = 8.1 Hz, 1H), 7.76 (t, J = 5.7 Hz, 1H), 7.65 (dd, J = 7.9, 1.2 Hz, 1H), 7.36 (d, J = 2.0 Hz, 1H), 7.31 – 7.28 (m, 1H), 7.16 (m, 1H), 7.08 – 7.03 (m, 2H), 6.97 (dd, J = 7.9, 2.1 Hz, 2H), 6.66 (dd, J = 7.3, 1.3 Hz, 1H), 4.99 – 4.87 (m, 2H), 4.74 (m, 1H), 4.57 – 4.49 (m, 2H), 4.18 (m, 2H), 4.05 (m, 1H), 3.11 – 3.05 (m, 2H), 2.80 (m, 1H), 2.71 (m, 1H), 2.32 (m, 1H), 2.06 – 1.95 (m, 4H), 1.76 (m, 2H), 1.64 (t, J = 7.4 Hz, 4H), 1.26 (s, 2H), 1.19 (m, 4H), 0.81 – 0.77 (m, 3H). ^{13}C NMR (126 MHz, $CDCl_3$) δ 179.04, 169.93, 167.67, 159.08, 156.41, 152.13, 147.19, 139.68, 137.23, 130.70, 124.76, 124.40, 124.07, 122.68, 121.60, 120.54, 119.55, 83.01, 69.22, 52.26, 52.14, 51.08, 46.50, 36.40, 34.57, 32.46, 32.31, 32.14, 31.36, 29.78, 27.04, 26.41, 22.44, 14.02, 13.40. ESI-MS: m/z 800.49 $[M-OTf-2H]^+$.

Synthesis of P-3

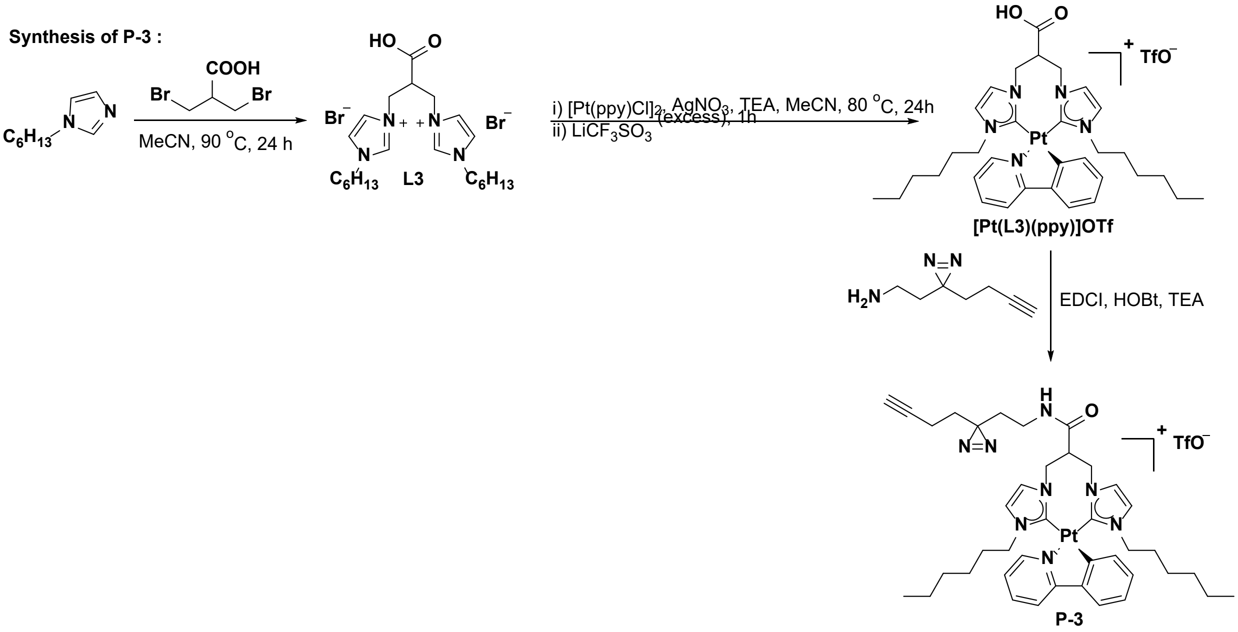

Scheme S5. Synthesis of P-3.

P-3 was synthesized following the synthetic route shown in Scheme S5. Briefly, the bis-NHC ligand, L3, was prepared by installing two functionalized arm chains step by step following the general procedure described above. Following the general synthetic procedure of Pt-NHC complexes described above, [Pt(L3)(ppy)]OTf was obtained after column chromatography on silica gel. ^{1}H NMR (500 MHz, $CDCl_3$) δ 8.04 (dd, J = 5.7, 1.6 Hz, 1H), 7.94 (m, 1H), 7.86 (d, J = 8.1 Hz, 1H), 7.66 (d, J = 7.7 Hz, 1H), 7.57 (d, J = 19.9 Hz, 2H), 7.18 – 7.12 (m, 2H), 7.12 – 7.07 (m, 1H), 6.98 (d, J = 1.7 Hz, 1H), 6.86 (d, J = 1.9 Hz, 1H), 6.79 – 6.70 (m, 1H), 4.90 (m, 2H), 4.66 (m, 2H), 4.43 – 4.30 (m, 2H), 4.01 – 3.89 (m, 2H), 3.12 (s, 1H), 1.73 (m, 4H), 1.28 – 1.18 (m, 12H), 0.85 – 0.77 (m, 6H). ^{13}C NMR (126 MHz, $CDCl_3$) δ 178.34, 167.91, 159.69, 155.62, 151.82, 147.03, 139.62, 137.35, 130.81, 124.56, 124.04, 123.83, 123.70, 123.17, 120.61, 119.78, 119.56, 55.08, 50.85, 50.40, 46.59, 31.39, 31.36, 30.58, 29.82, 26.61, 22.50, 14.02, 8.82. ESI-MS: m/z 737.63 $[M\text{-}OTf\text{-}2H]^{+}$.

Next, to a solution of [Pt(L3)(ppy)]OTf (1 mmol equiv.) in dry DCM, 1-hydroxybenzotriazole hydrate (HOBt, 2 mmol equiv.), 1-ethyl-3-(3-dimethylaminopropyl)carbodiimide (3 mmol equiv.) and triethylamine (TEA, 5 mmol equiv.) was added. After being stirred for overnight, the reaction mixture was dissolved in water and extracted with DCM. The product was then purified by column chromatography on silica gel using 20:1 to 10:1 DCM: MeOH as eluent. Yield: 24 %. ^{1}H NMR (400 MHz, $CDCl_3$) δ 8.40 (d, J = 42.2 Hz, 1H), 8.03 – 7.85 (m, 3H), 7.67 (m, 1H), 7.55 – 7.28 (m, 2H), 7.17 (m, 1H), 7.12 – 7.04 (m, 2H), 6.95 (dd, J = 15.1, 2.0 Hz, 1H), 6.87 (dd, J = 6.4, 2.0 Hz, 1H), 6.73 – 6.64 (m, 1H), 5.20 – 5.14 (m, 1H), 4.97 (m, 1H), 4.65 – 4.26 (m, 4H), 4.02 – 3.90 (m, 2H), 3.34 (m, 1H), 3.12 (m, 2H), 1.99 (m, 3H), 1.85 – 1.67 (m, 8H), 1.29 – 1.21 (m, 12H), 0.83 (m, 6H). ^{13}C NMR (126 MHz, $CDCl_3$) δ 179.10, 170.80, 168.26, 159.19, 155.91, 151.35, 146.78,

139.60, 137.24, 131.00, 124.69, 124.40, 124.14, 123.75, 123.27, 120.23, 119.76, 119.49, 83.01, 69.19, 54.86, 54.78, 50.84, 50.42, 48.31, 44.74, 34.60, 32.17, 31.84, 31.35, 31.31, 30.56, 30.47, 29.78, 29.72, 26.56, 22.44, 13.96, 13.34. ESI-MS: m/z 856.30 $[M-OTf-2H]^+$.

Supplementary Tables

Table S1. Cytotoxicity of synthesized probes determined by WST-8 cell viability assay.

IC50	**Pt-NHC**	**Pt-ER**	**P-1**	**P-2a**	**P-2b**	**P-3**
A2780	1.37 ± 0.30	0.71 ± 0.31	1.87 ± 0.43	2.67 ± 0.35	2.3 ± 0.38	1.95 ± 0.16
CT26	5.49 ± 0.88	2.72 ± 0.13	12.69 ± 0.40	23.9 ± 4.53	22.38 ± 4.88	6.66 ± 0.24
HCT116	4.99 ± 1.67	5.20 ± 2.08	6.26 ± 0.36	6.18 ± 0.86	7.45 ± 0.12	5.47 ± 0.71

Notes: Data were acquired from WST-8 assay after 48 h incubation and shown as Mean ± S.D. (n = 3 independently biological replicates).

Supplementary figures

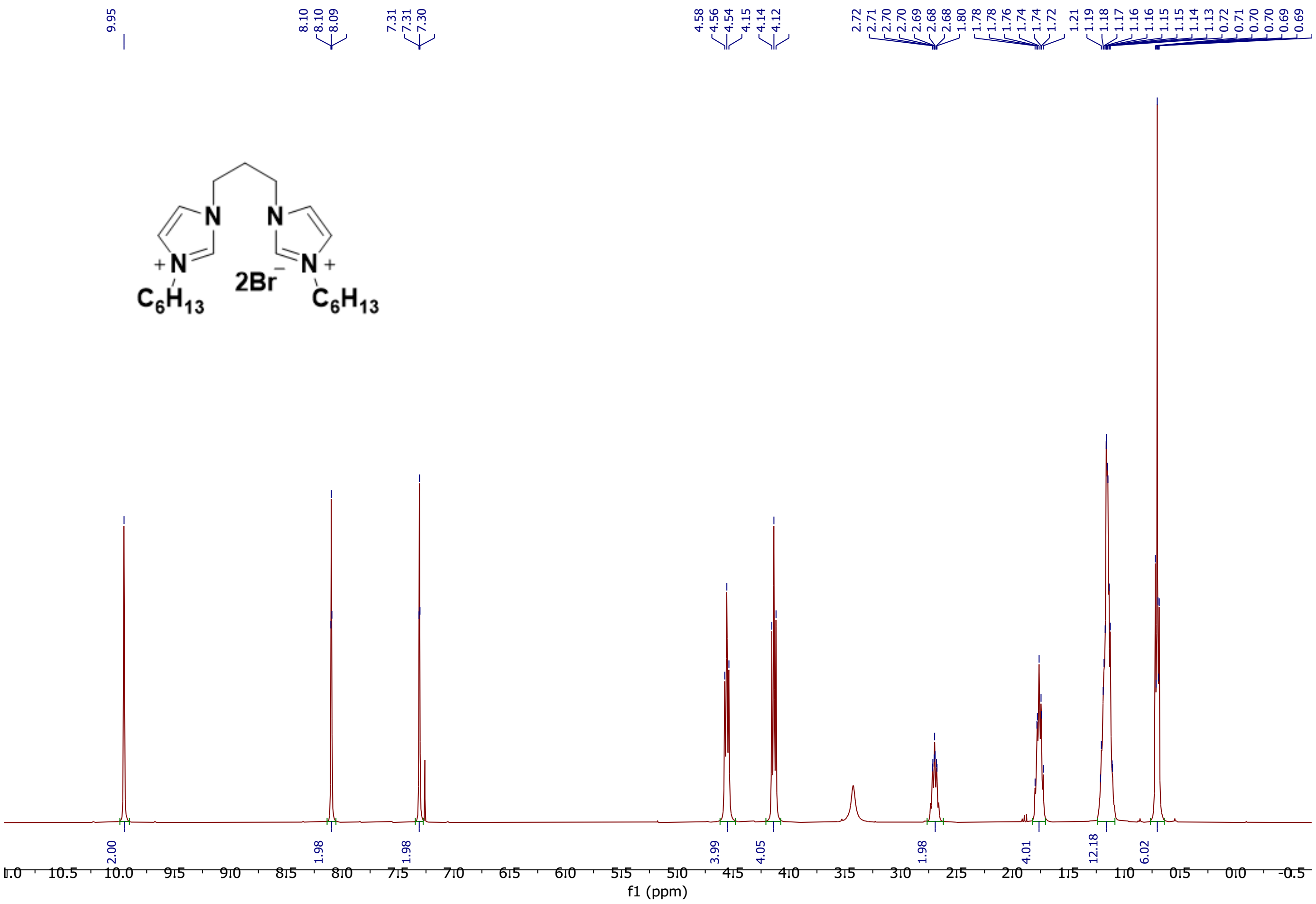

Figure S1. ^{1}H-NMR spectrum of ligand of Pt-ER

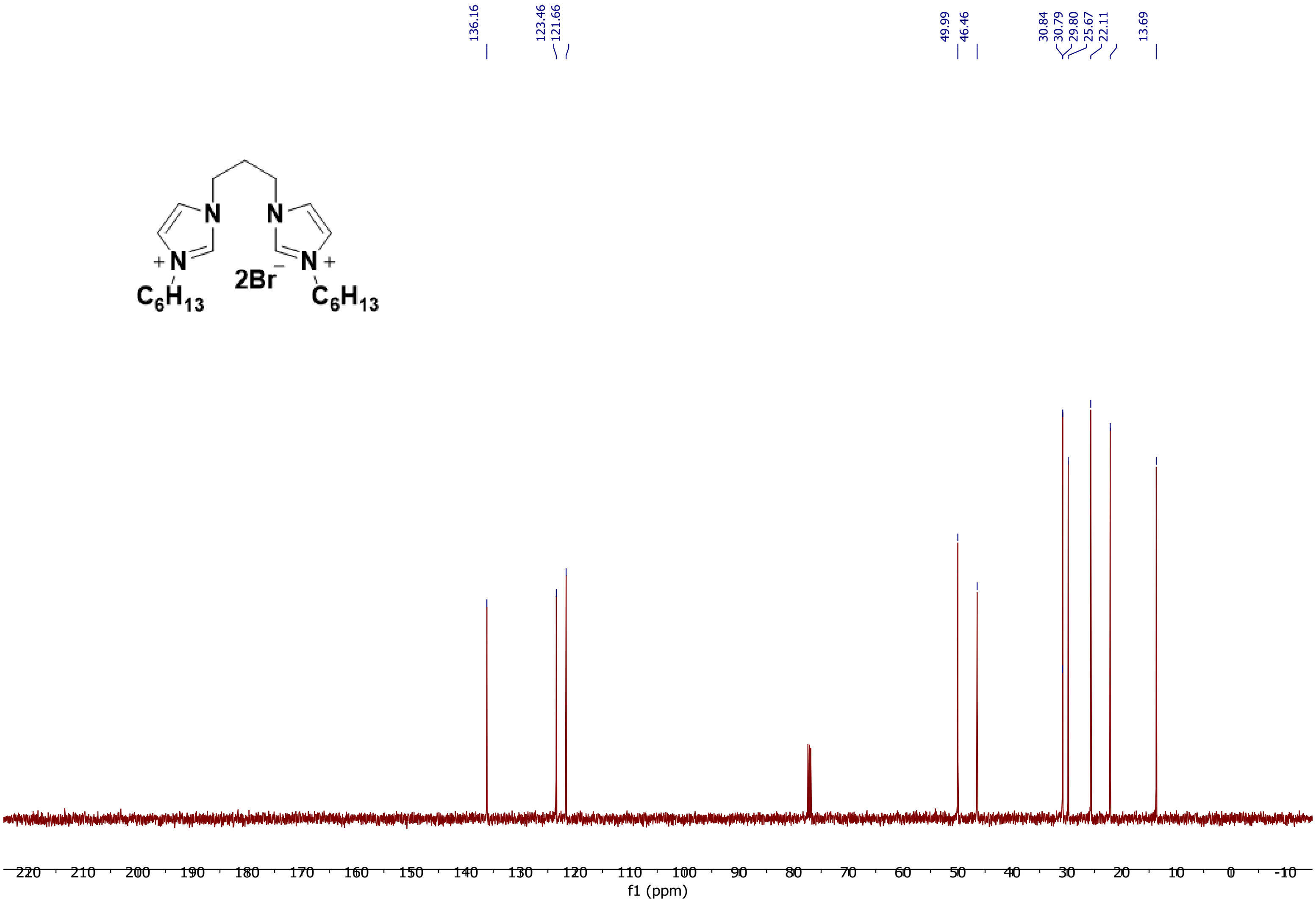

Figure S2. ^{13}C-NMR spectrum of ligand of Pt-ER

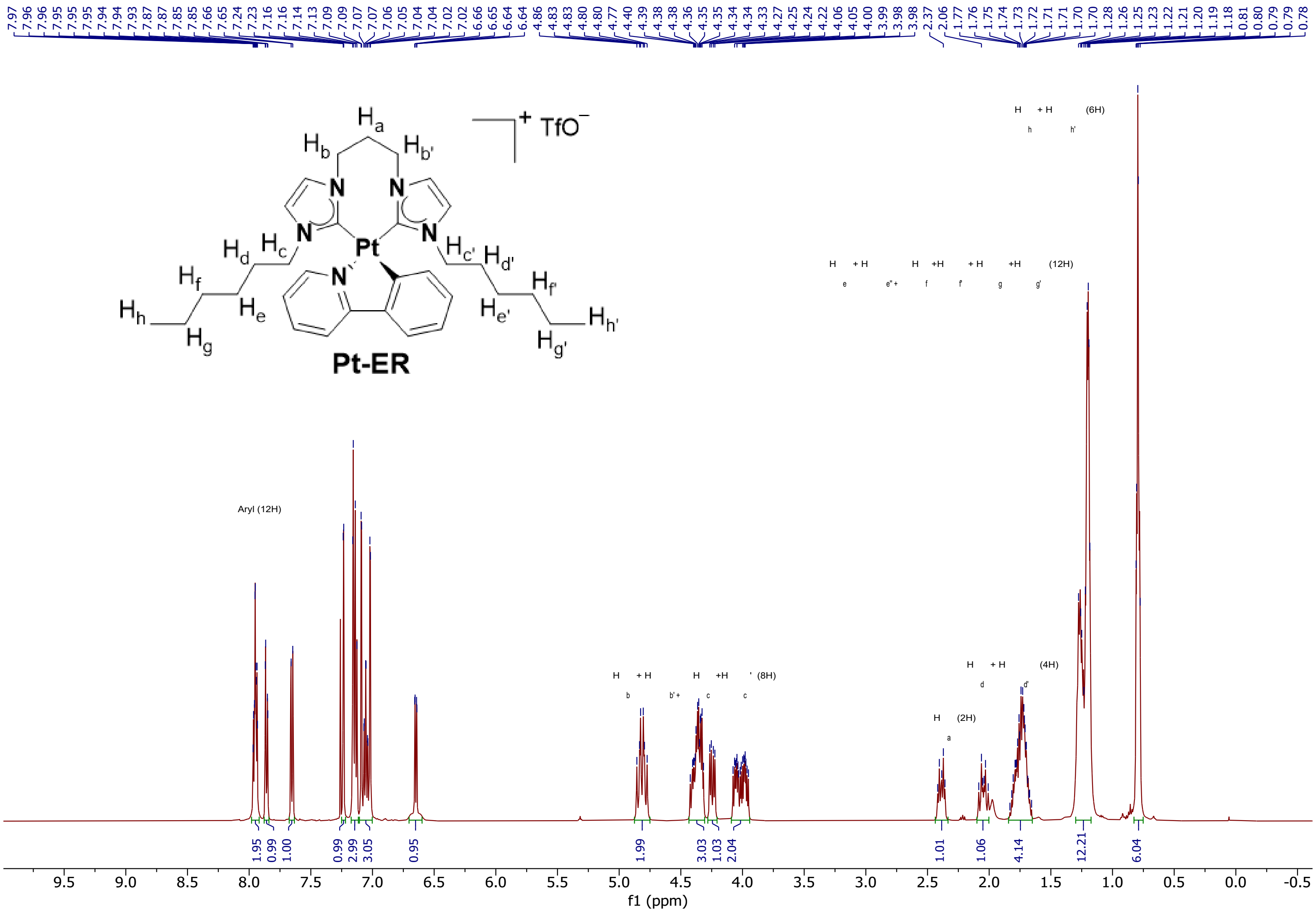

Figure S3. ^{1}H-NMR spectrum of Pt-ER

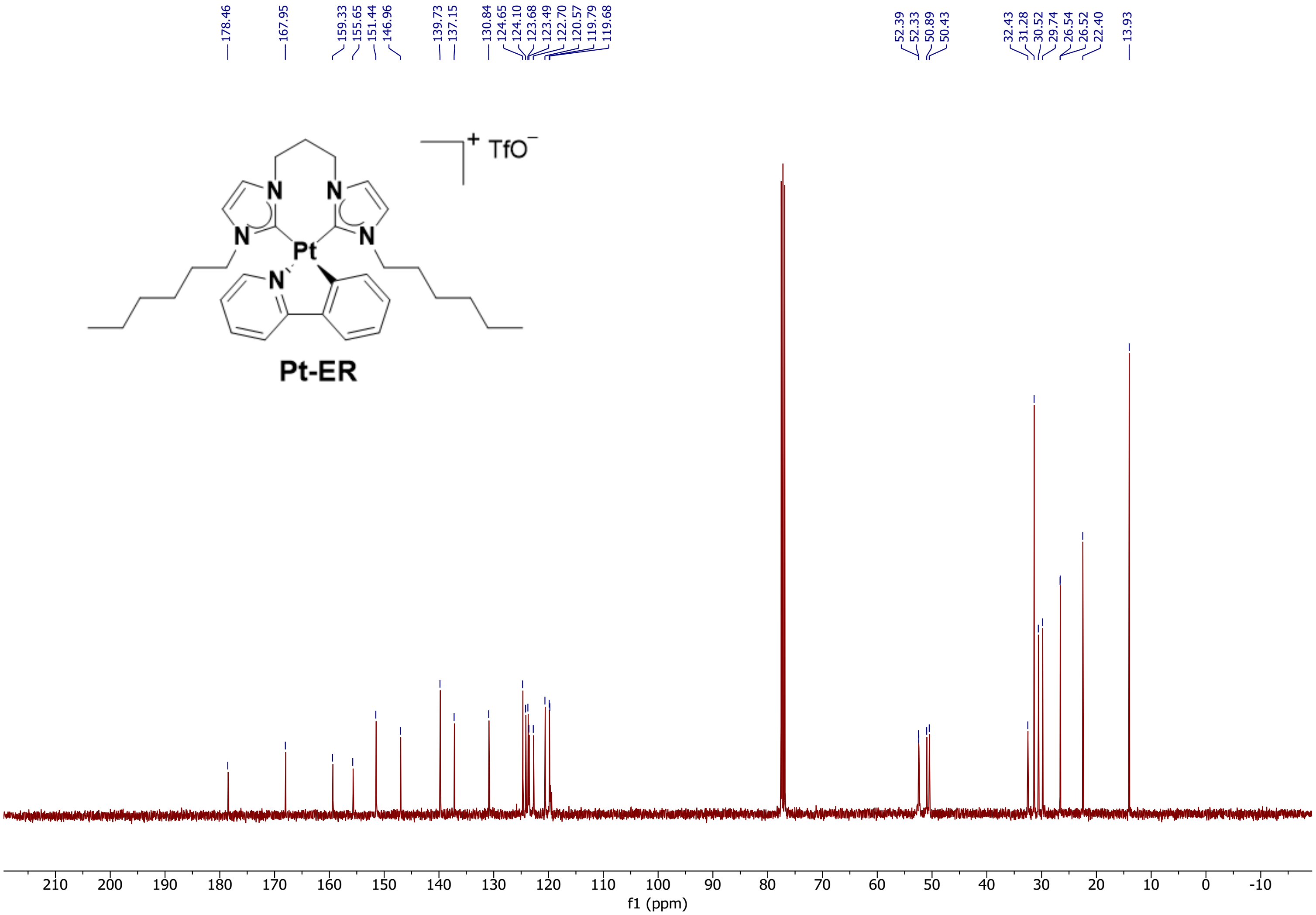

Figure S4. ^{13}C-NMR spectrum of Pt-ER

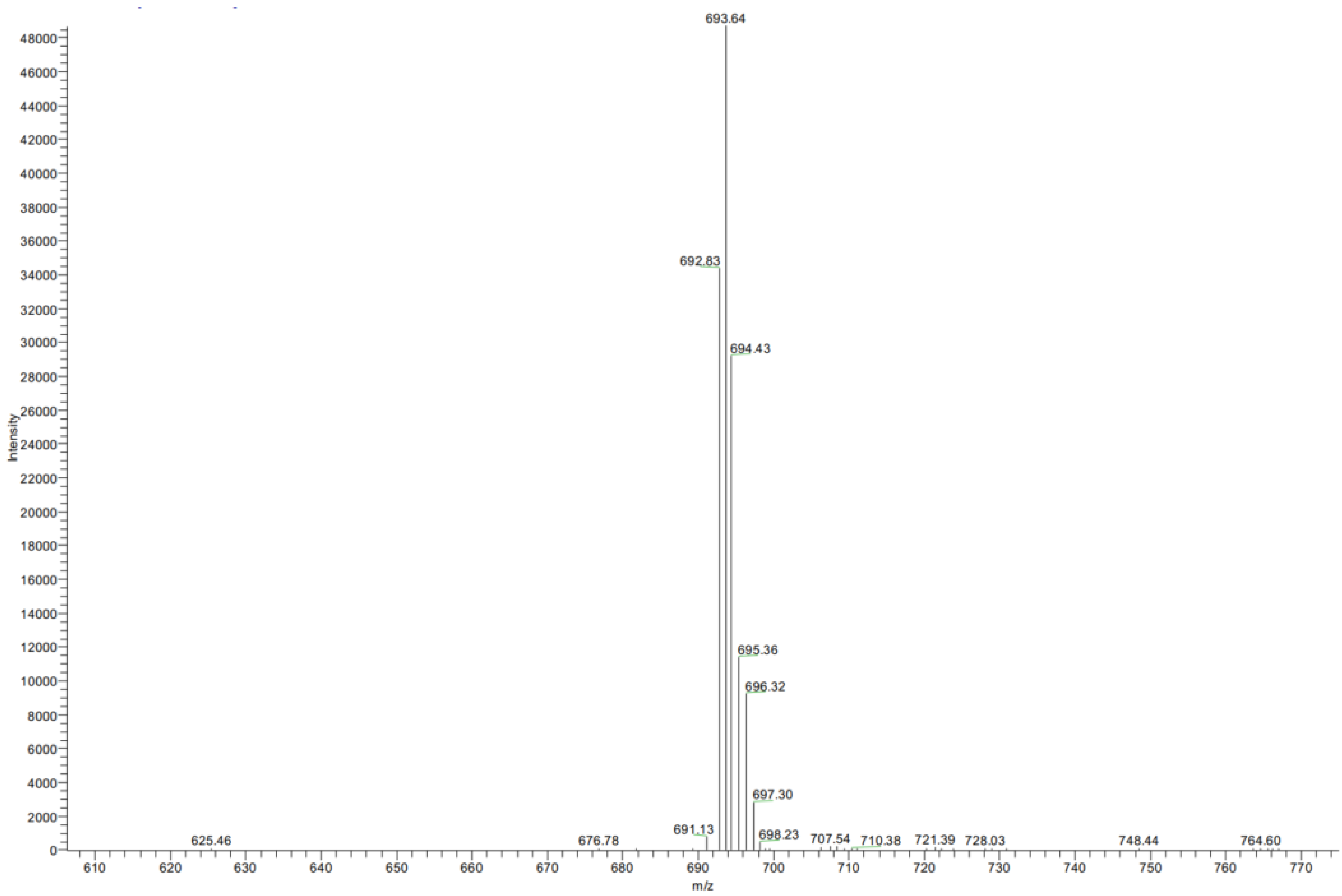

Figure S5. ESI-MS spectrum of Pt-ER

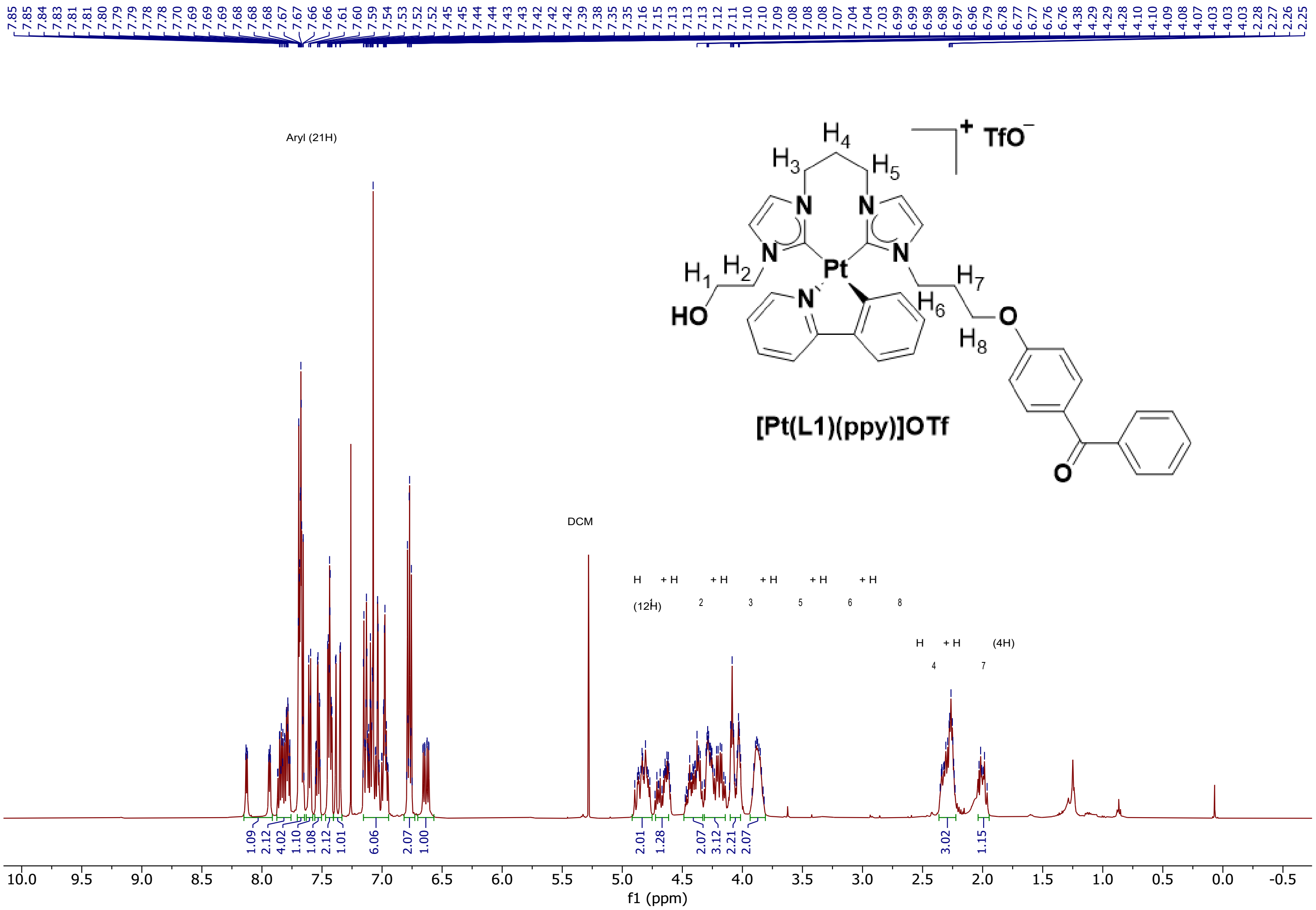

Figure S6. ^{1}H-NMR spectrum of P-1 precursor [Pt(L1)(ppy)]OTf

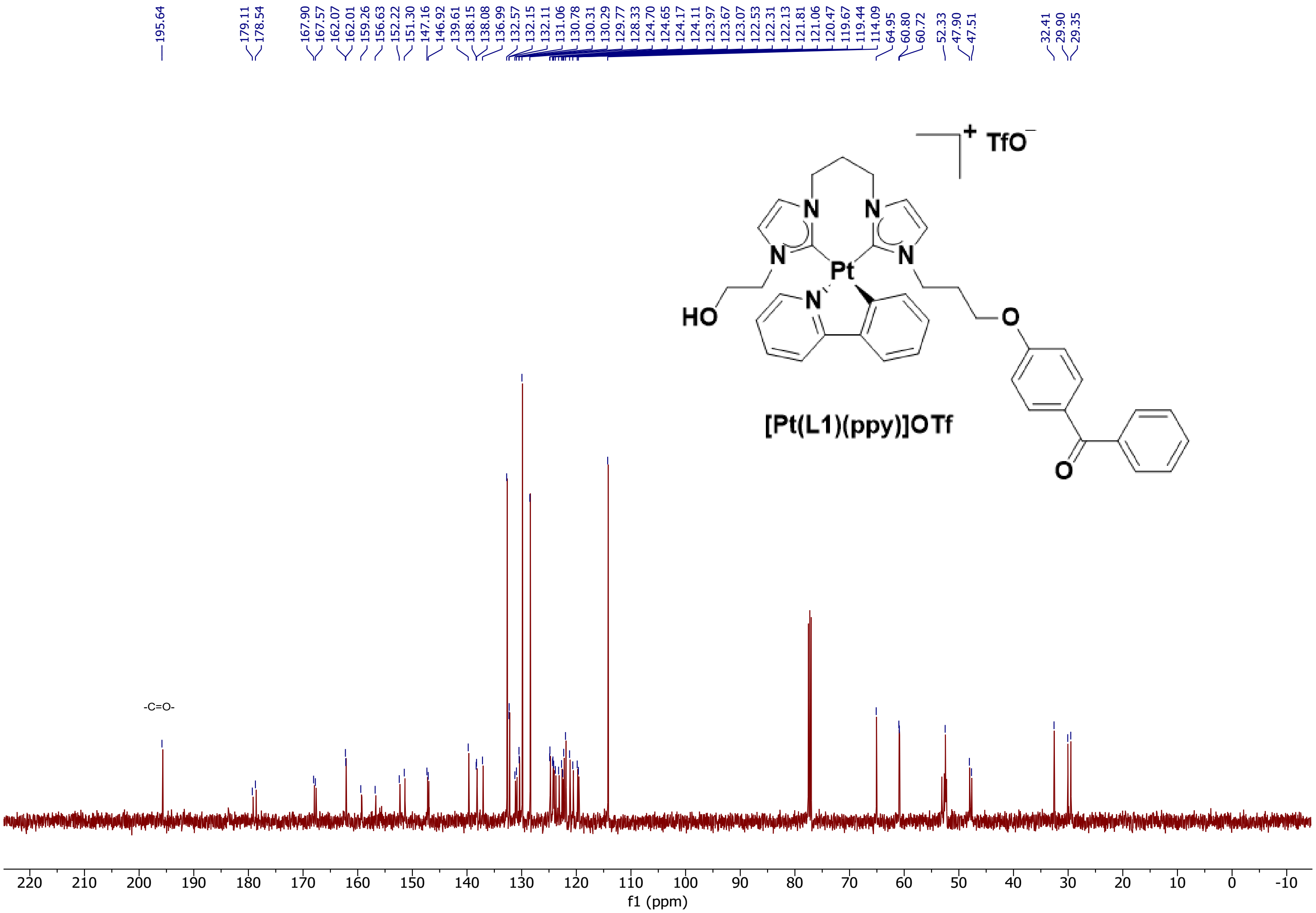

Figure S7. ^{13}C-NMR spectrum of P-1 precursor [Pt(L1)(ppy)]OTf

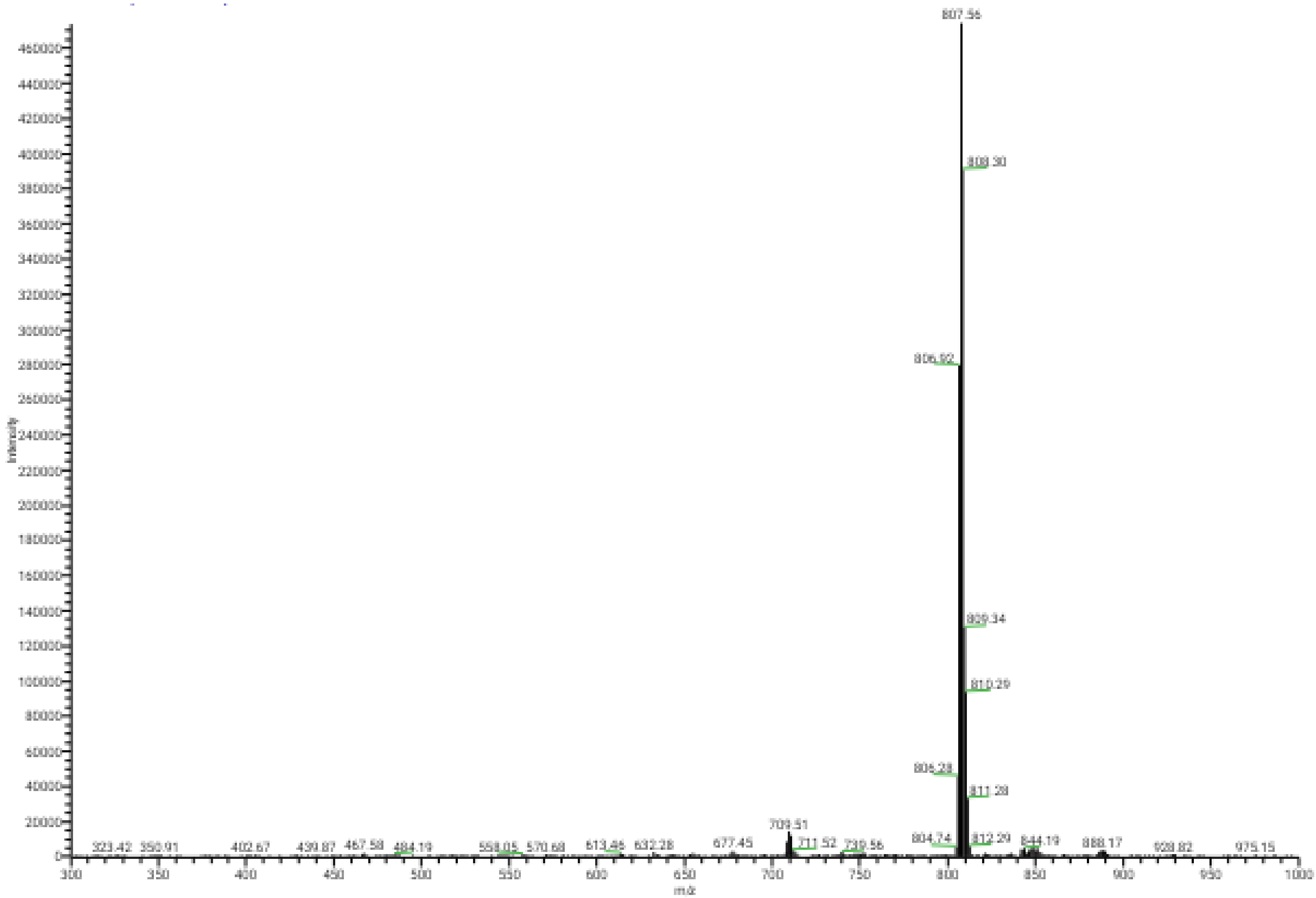

Figure S8. ESI-MS spectrum of P-1 precursor [Pt(L1)(ppy)]OTf

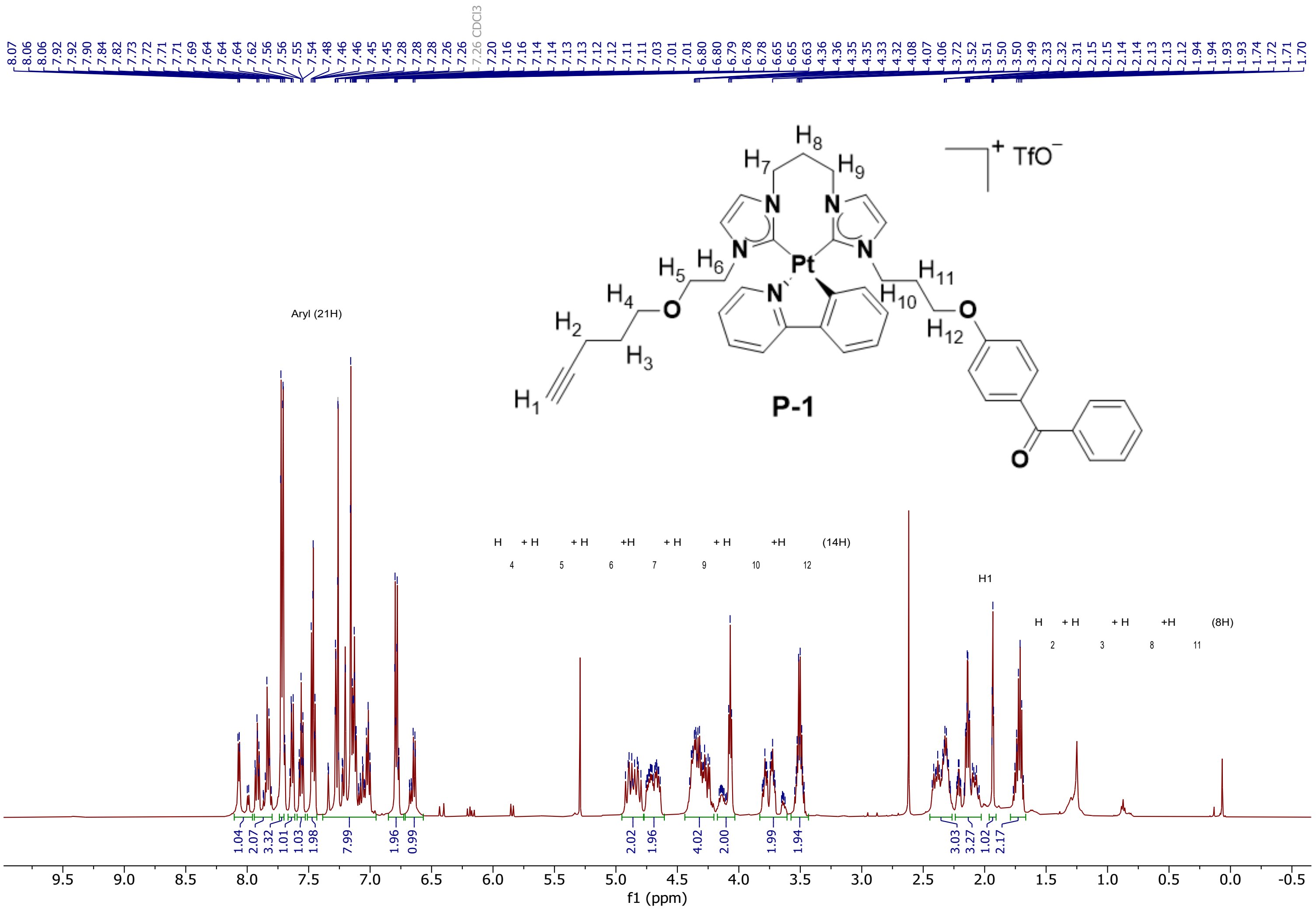

Figure S9. ^{1}H-NMR spectrum of P-1

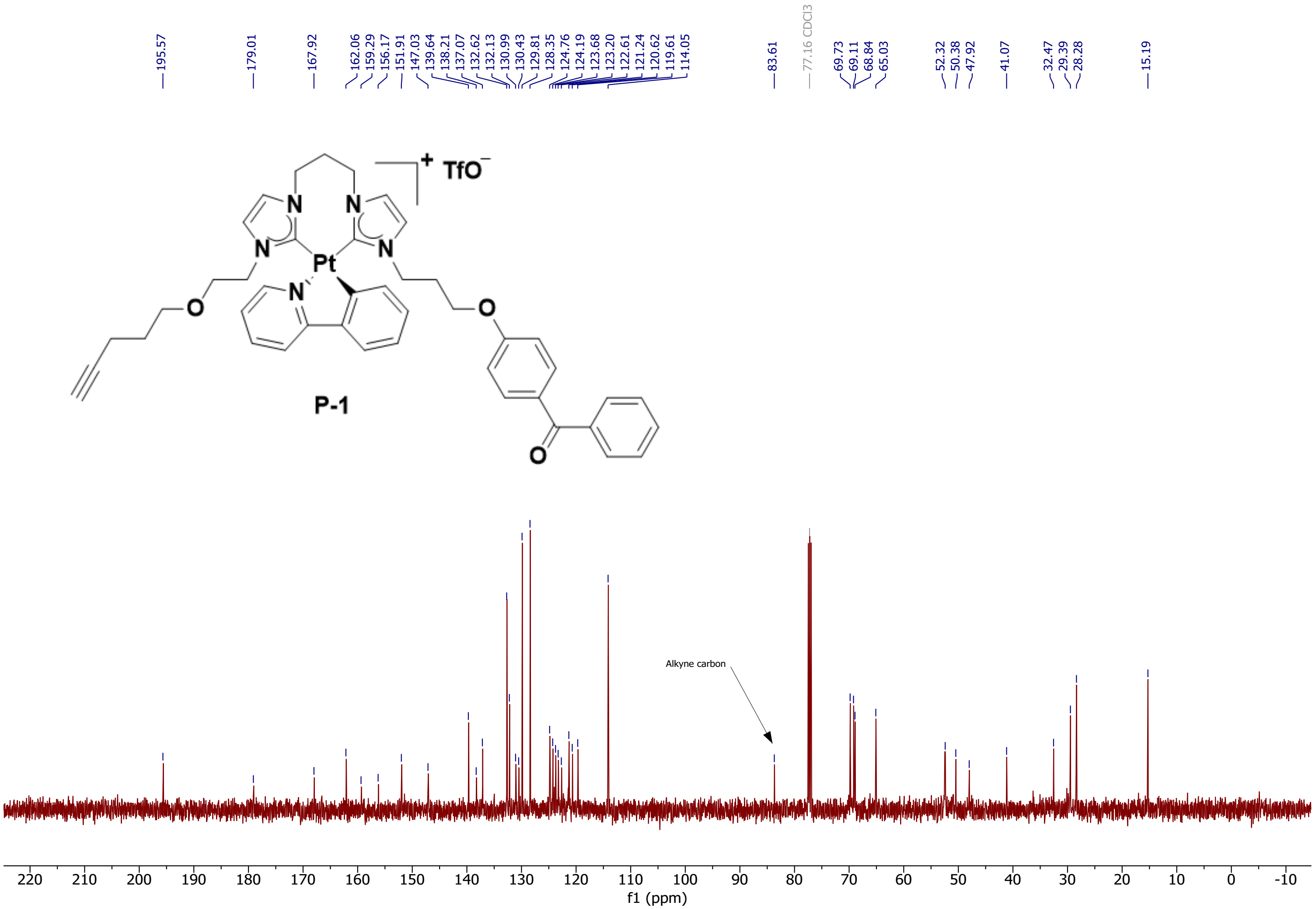

Figure S10. ^{13}C-NMR spectrum of P-1

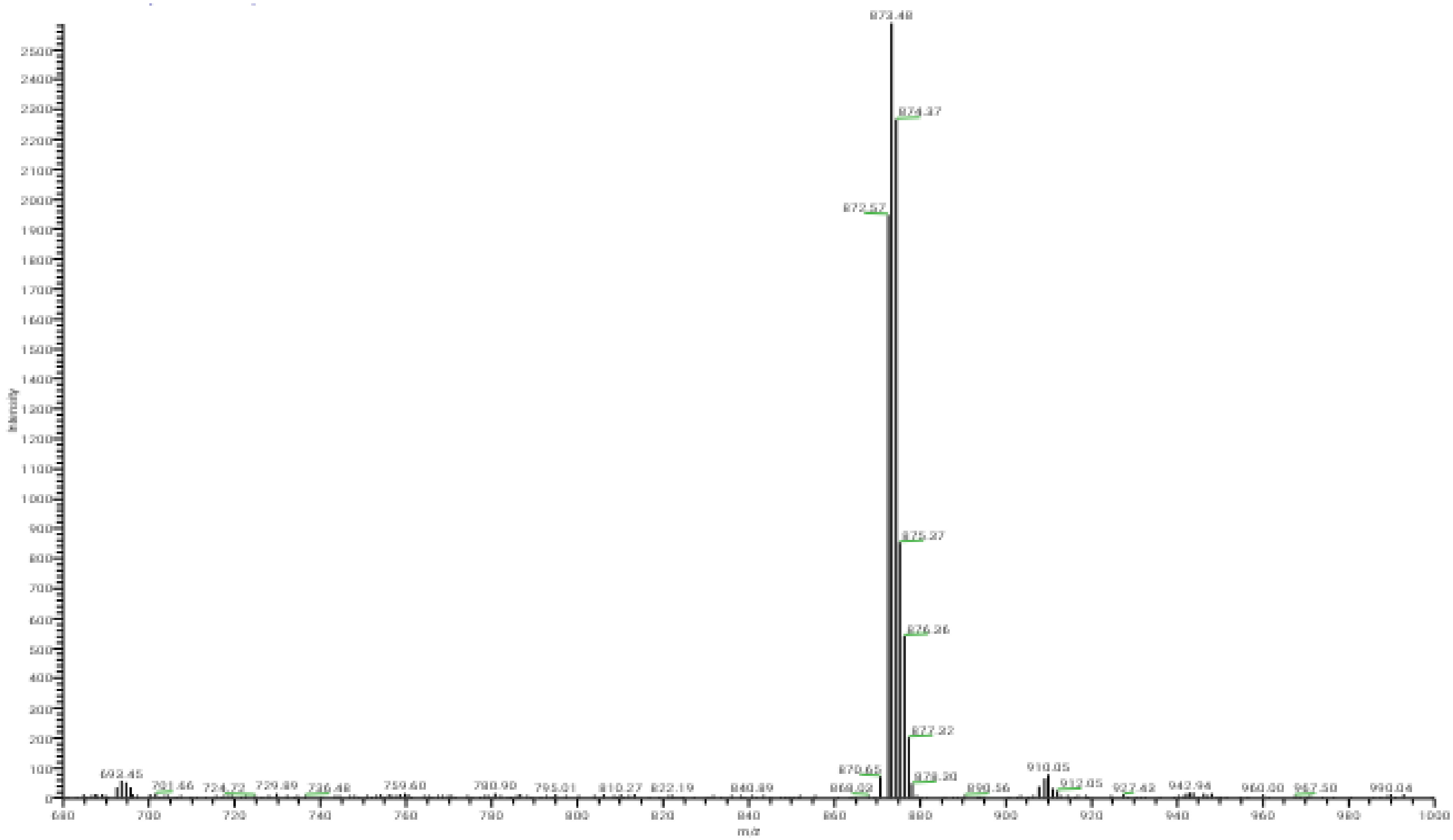

Figure S11. ESI-MS spectrum of P-1

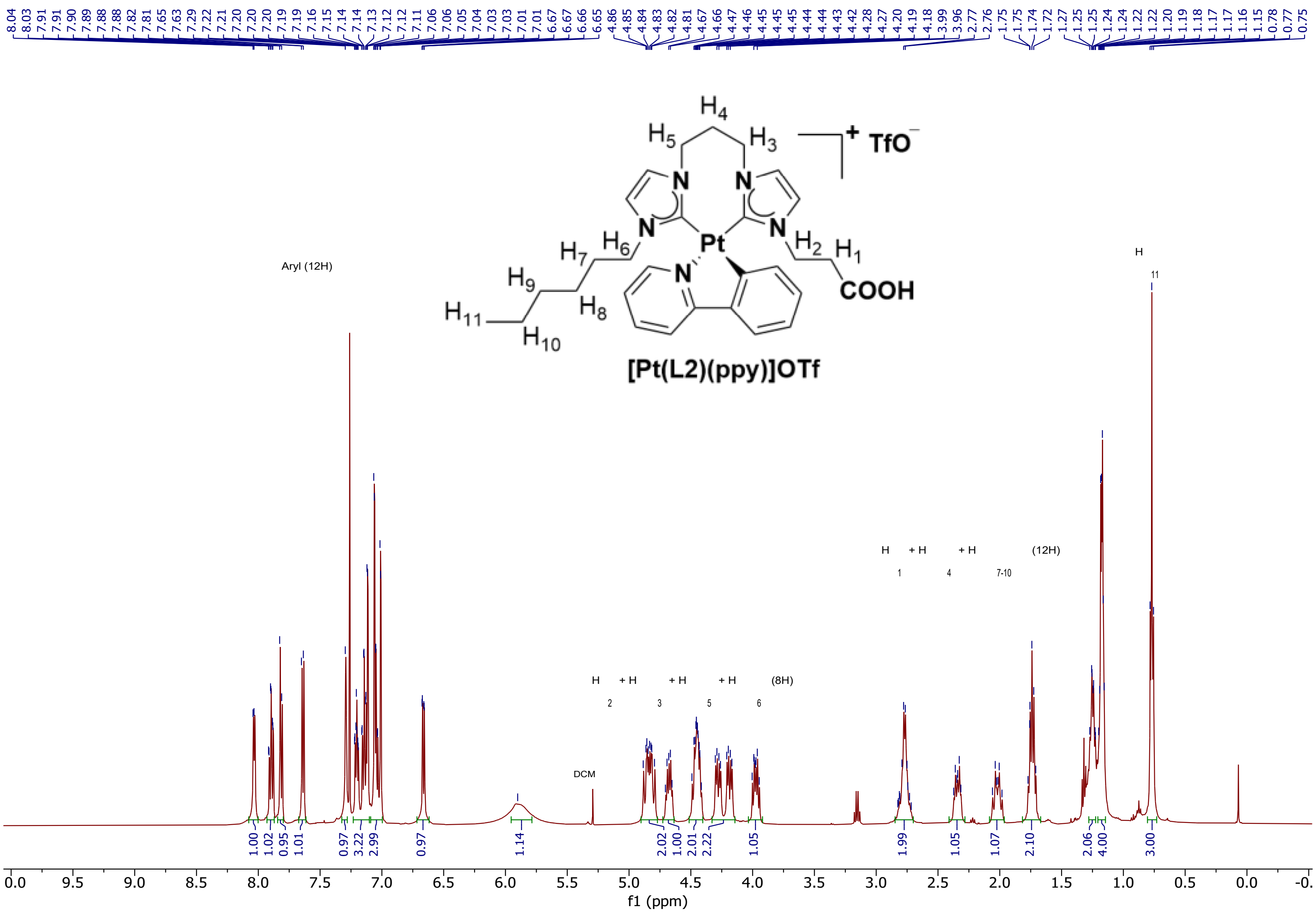

Figure S12. ESI-MS spectrum of P-1

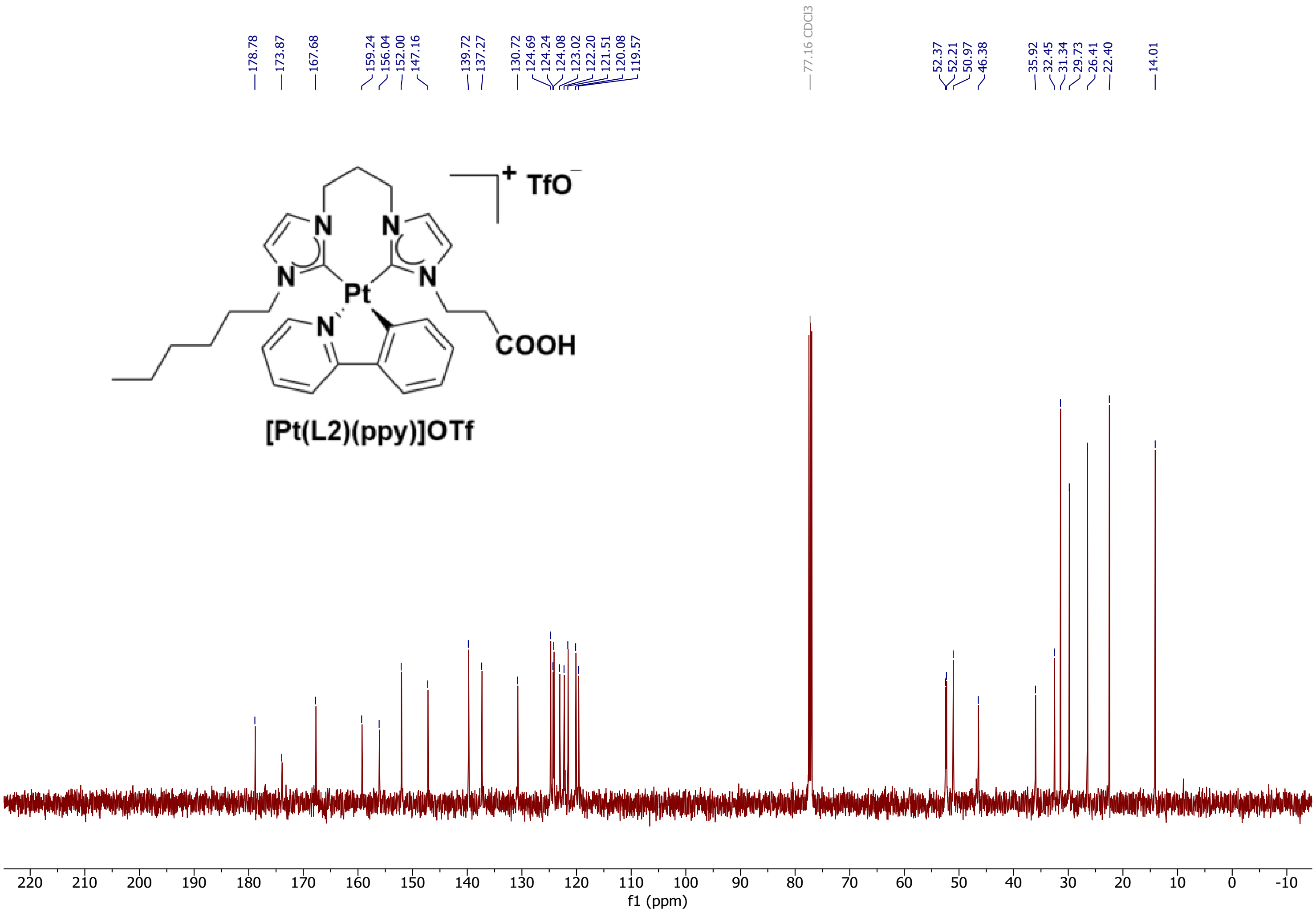

Figure S13. ^{13}C-NMR spectrum of P-2 precursor [Pt(L2)(ppy)]OTf

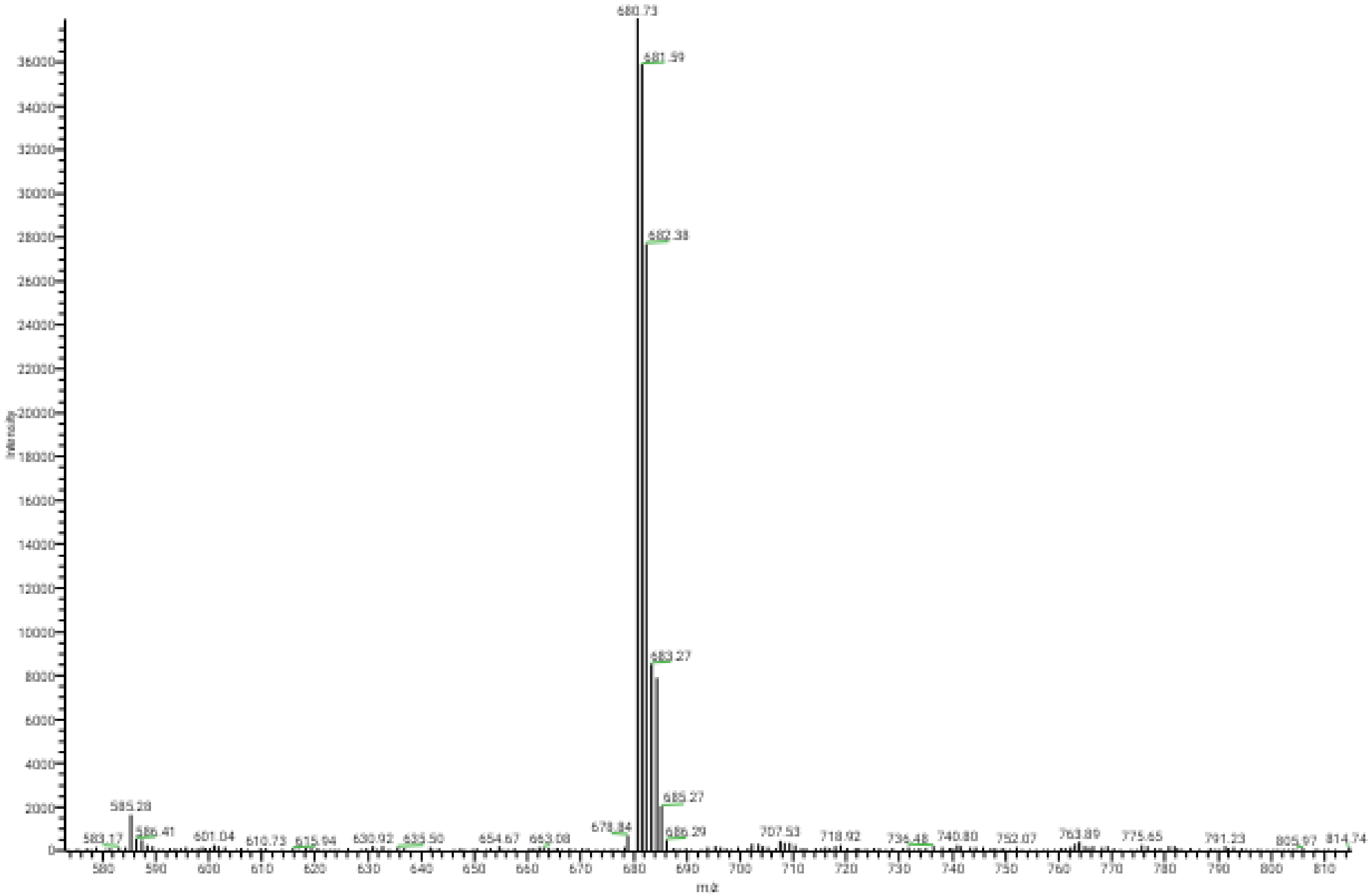

Figure S14. ESI-MS spectrum of P-2 precursor [Pt(L2)(ppy)]OTf

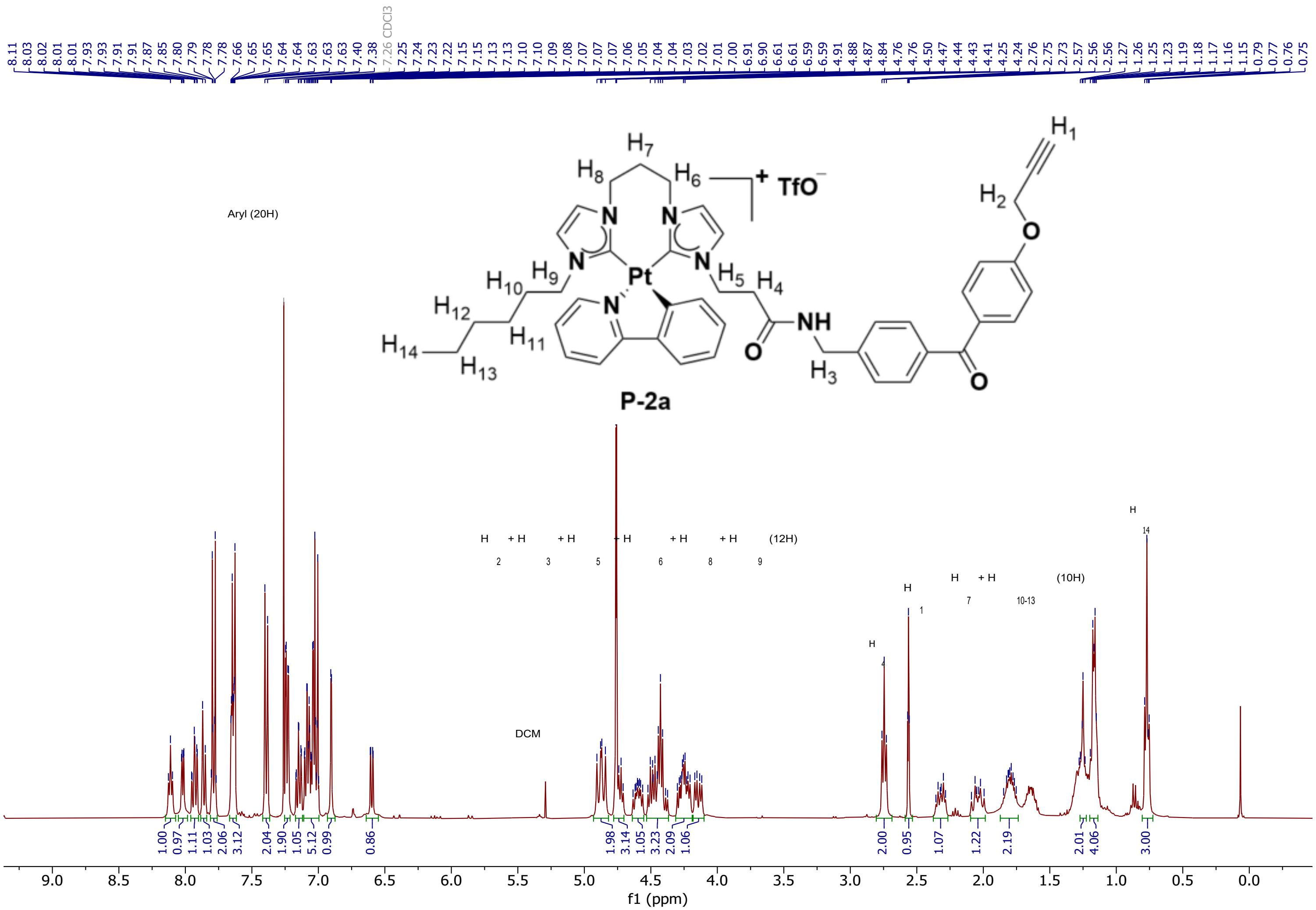

Figure S15. ^{1}H-NMR spectrum of P-2a

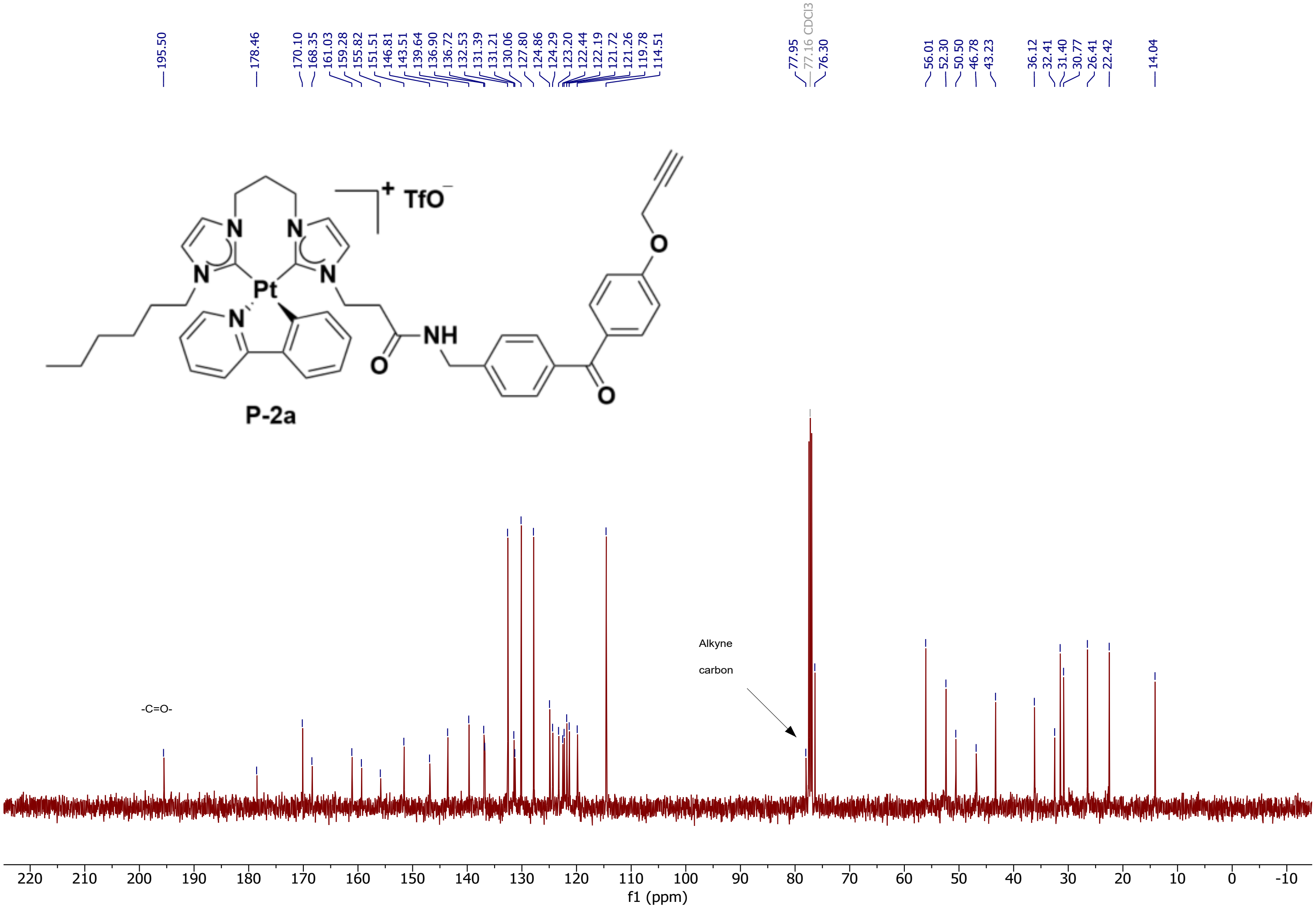

Figure S16. ^{13}C-NMR spectrum of P-2a

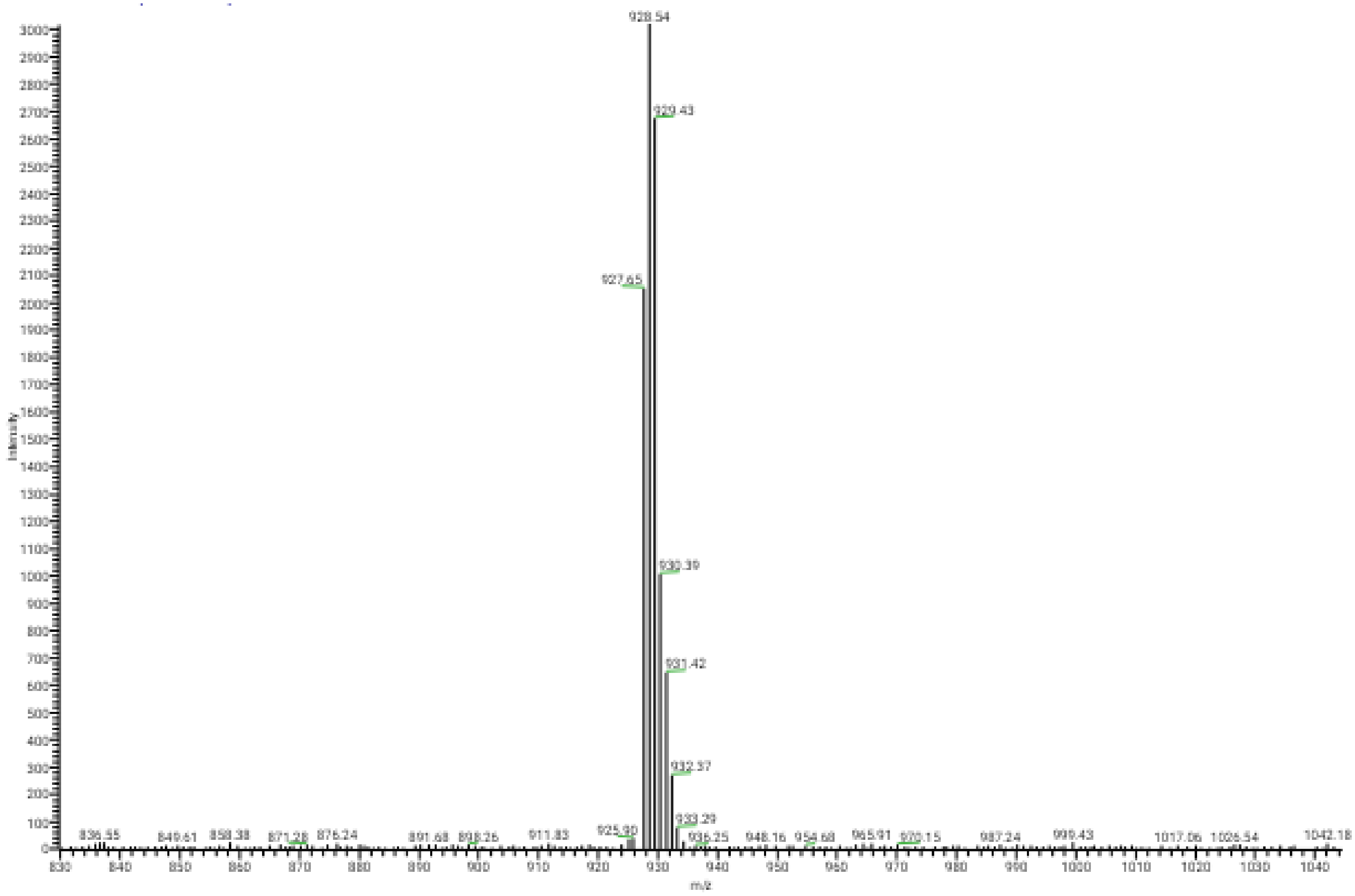

Figure S17. ESI-MS spectrum of P-2a

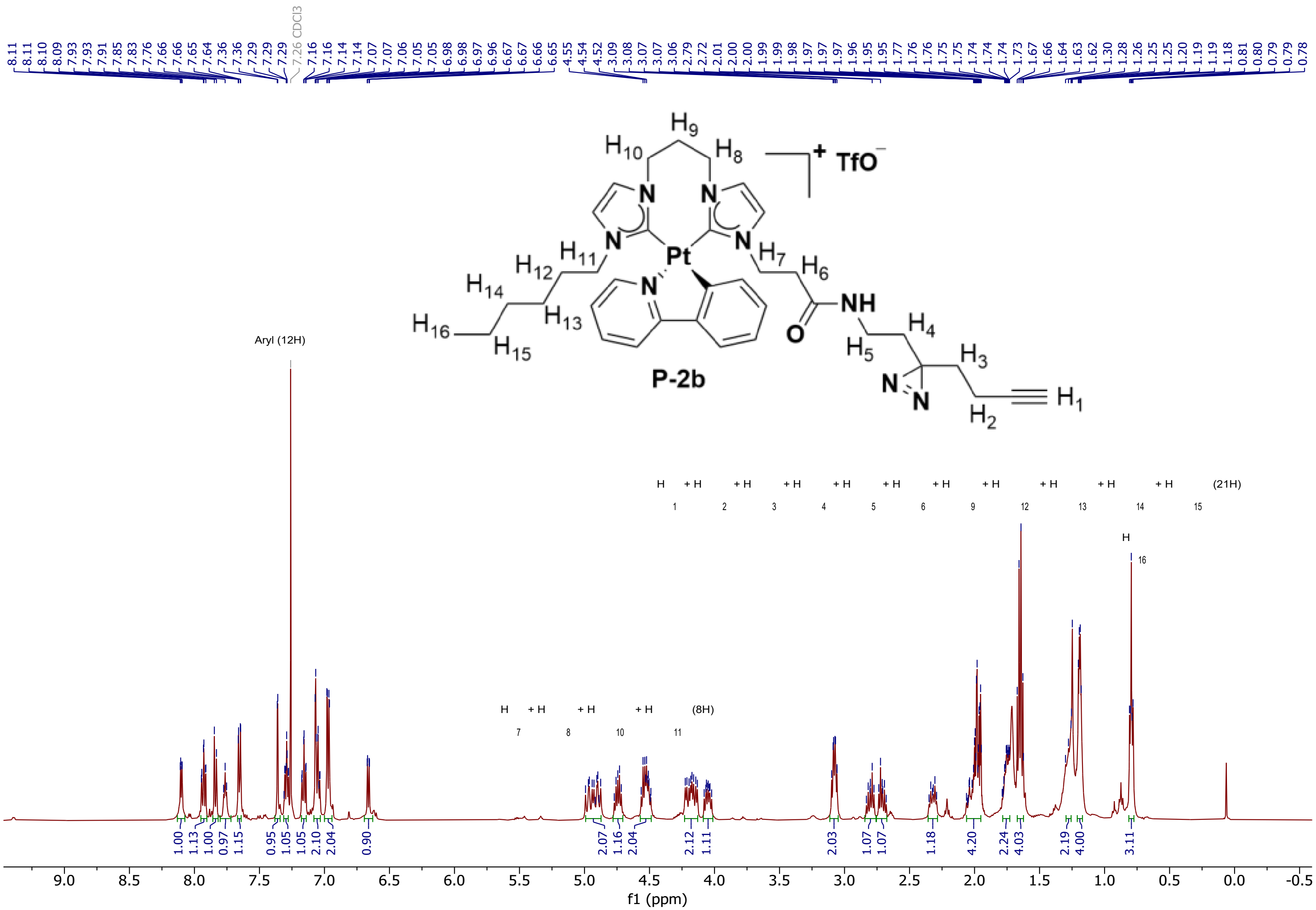

Figure S18. ^{1}H-NMR spectrum of P-2b

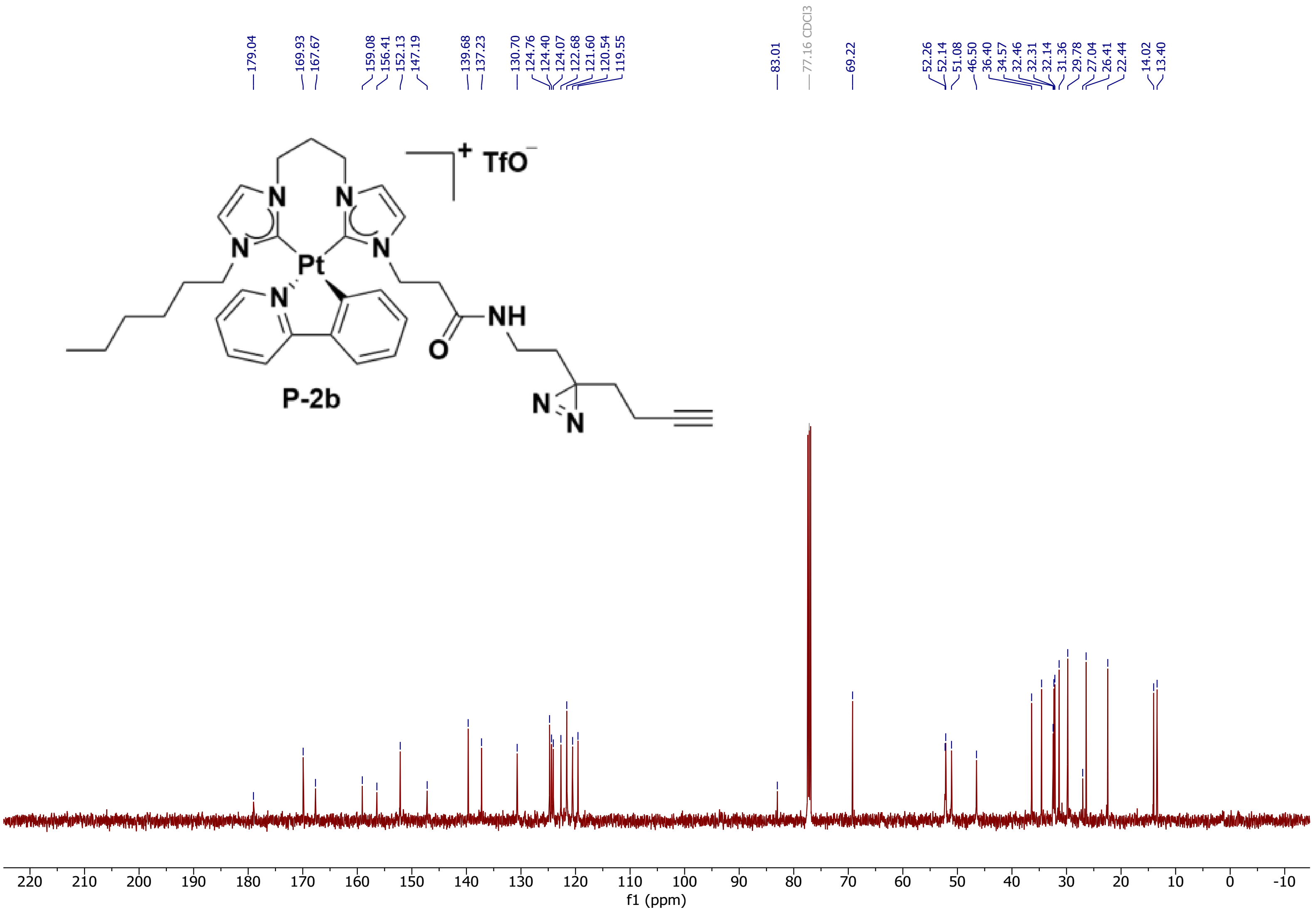

Figure S19. ^{13}C-NMR spectrum of P-2b

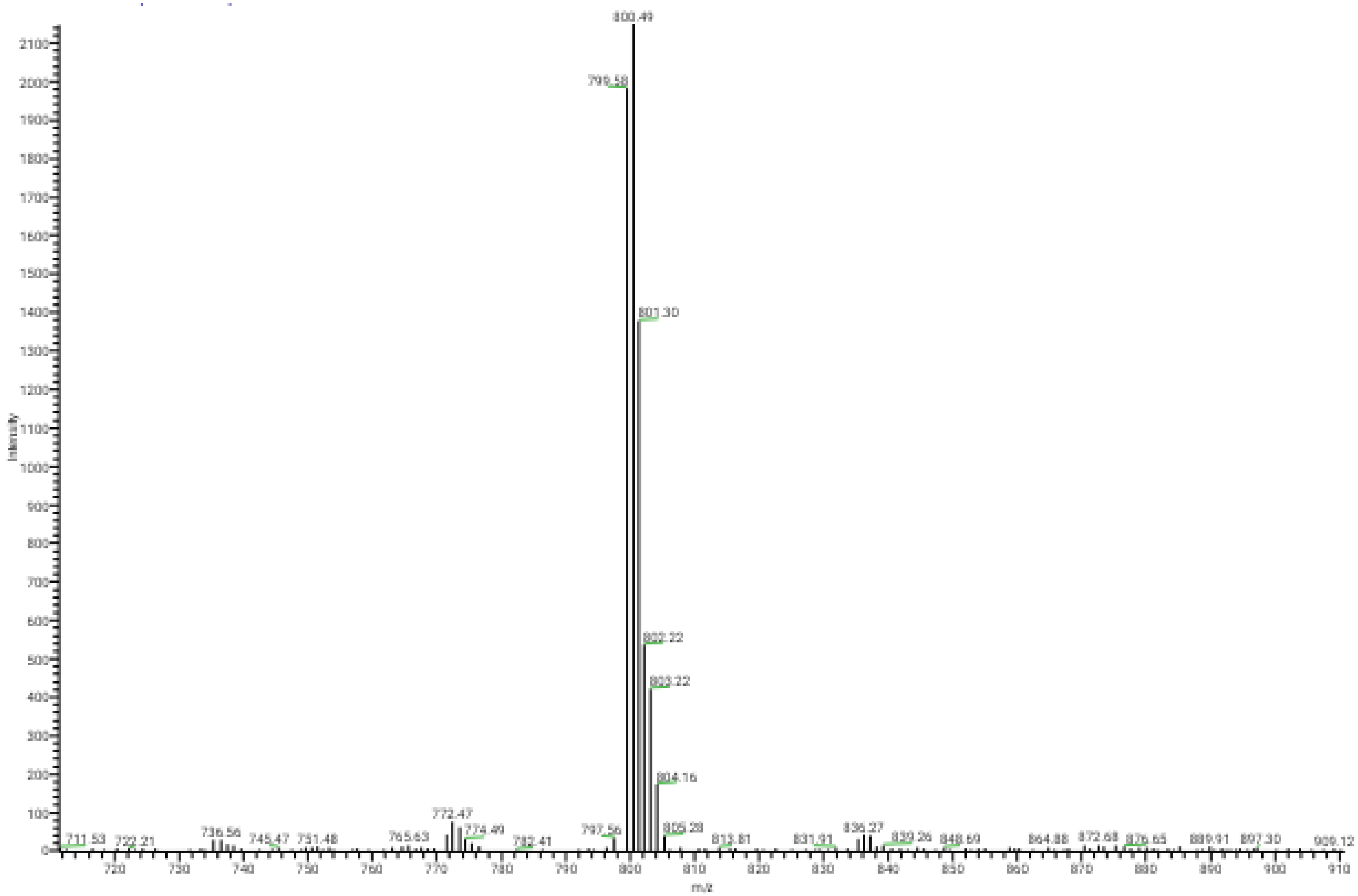

Figure S20. ESI-MS spectrum of P-2b

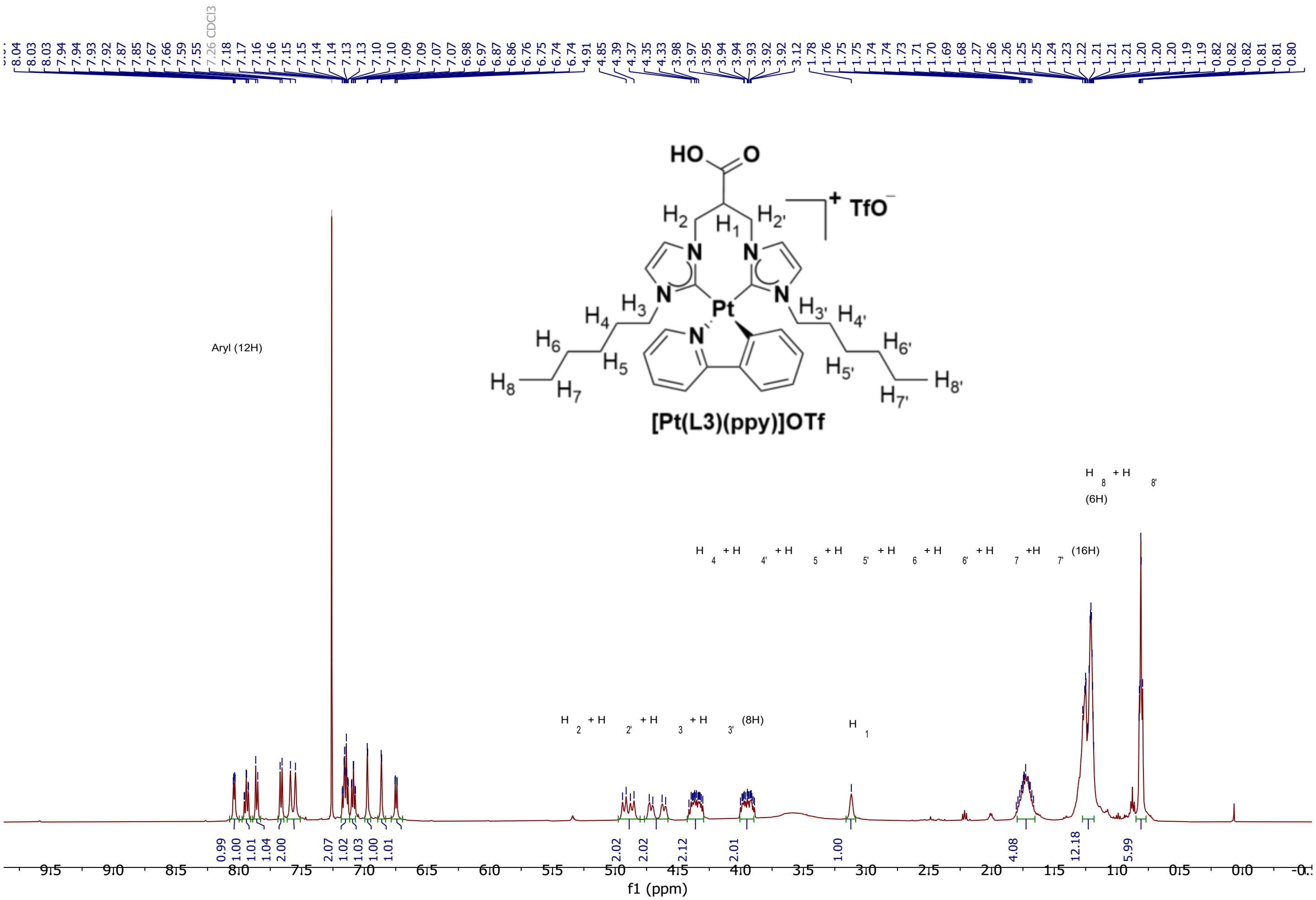

Figure S21. ¹H-NMR spectrum of P-3 precursor [Pt(L3)(ppy)]OTf

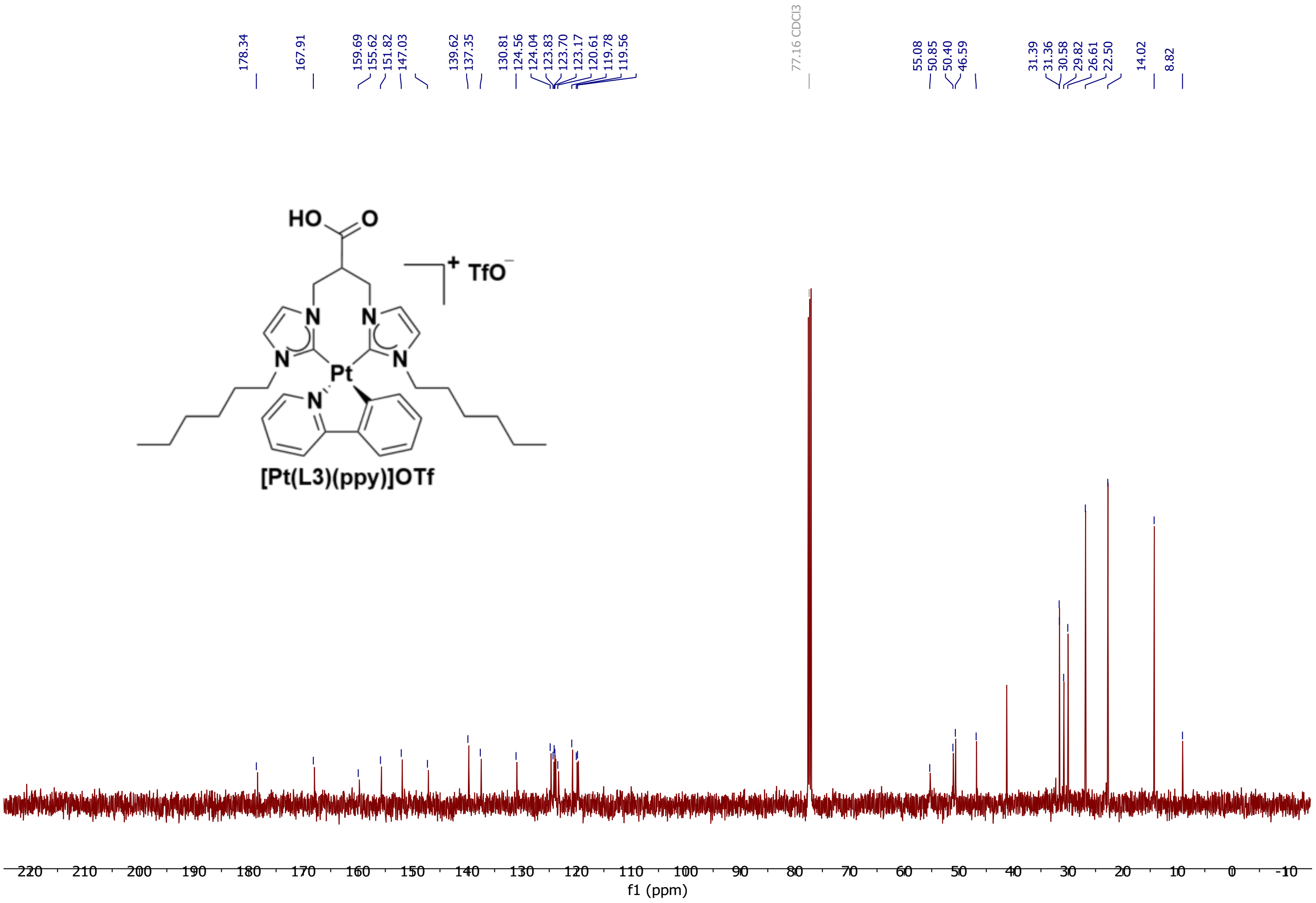

Figure S22. ^{13}C-NMR spectrum of P-3 precursor [Pt(L3)(ppy)]OTf

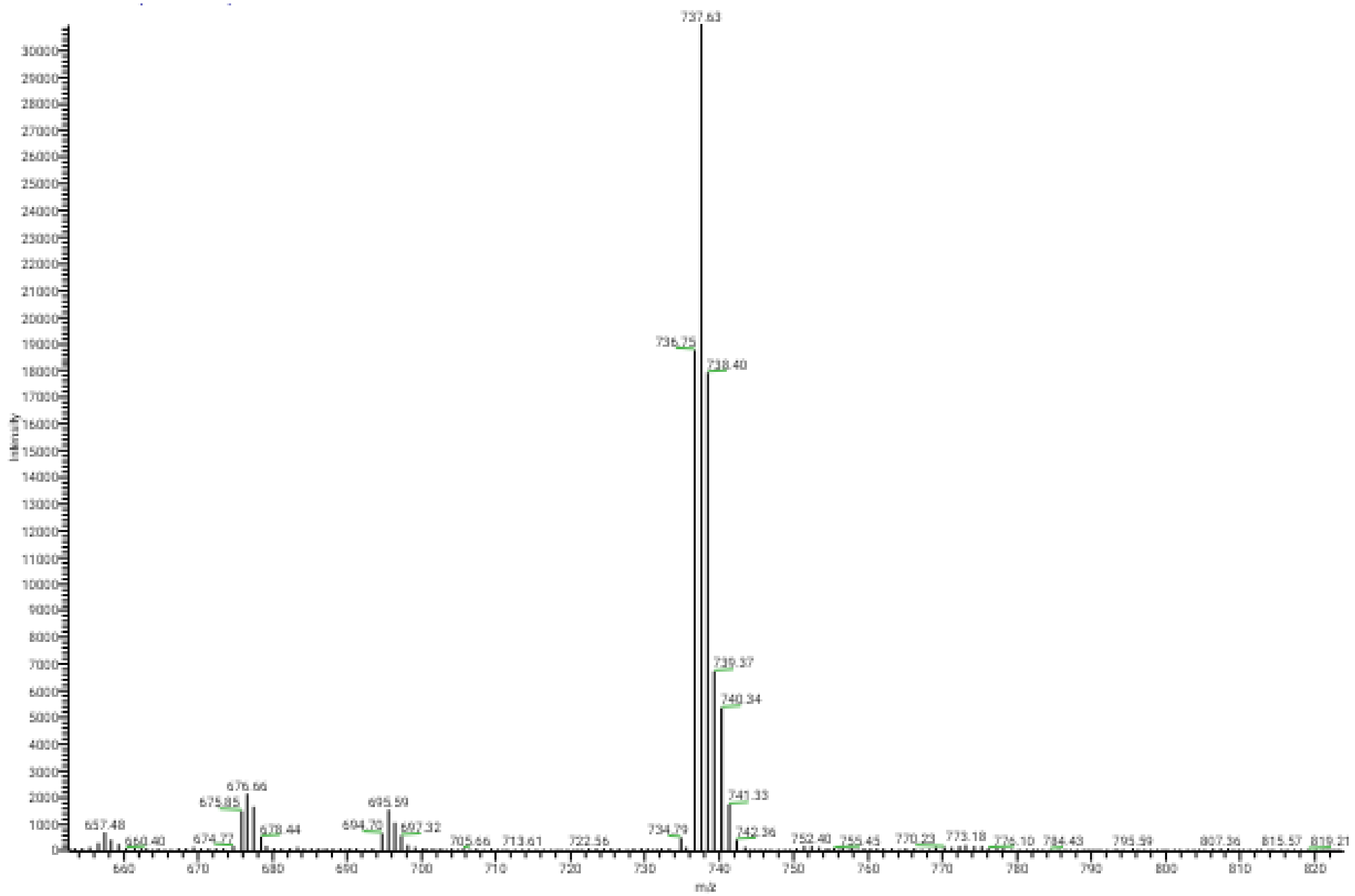

Figure S23. ESI-MS spectrum of P-3 precursor [Pt(L3)(ppy)]OTf

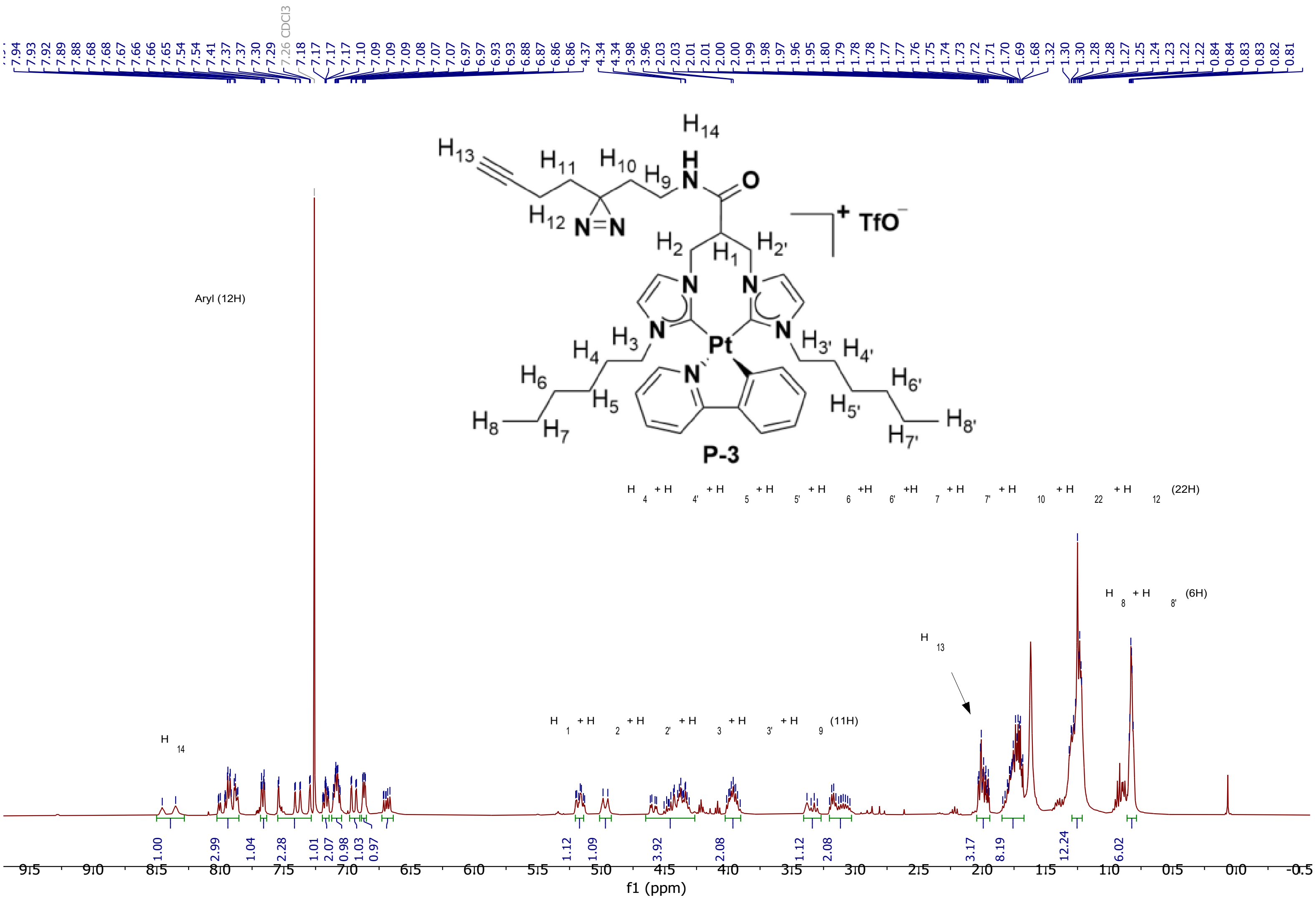

Figure S24. ^{1}H-NMR spectrum of P-3

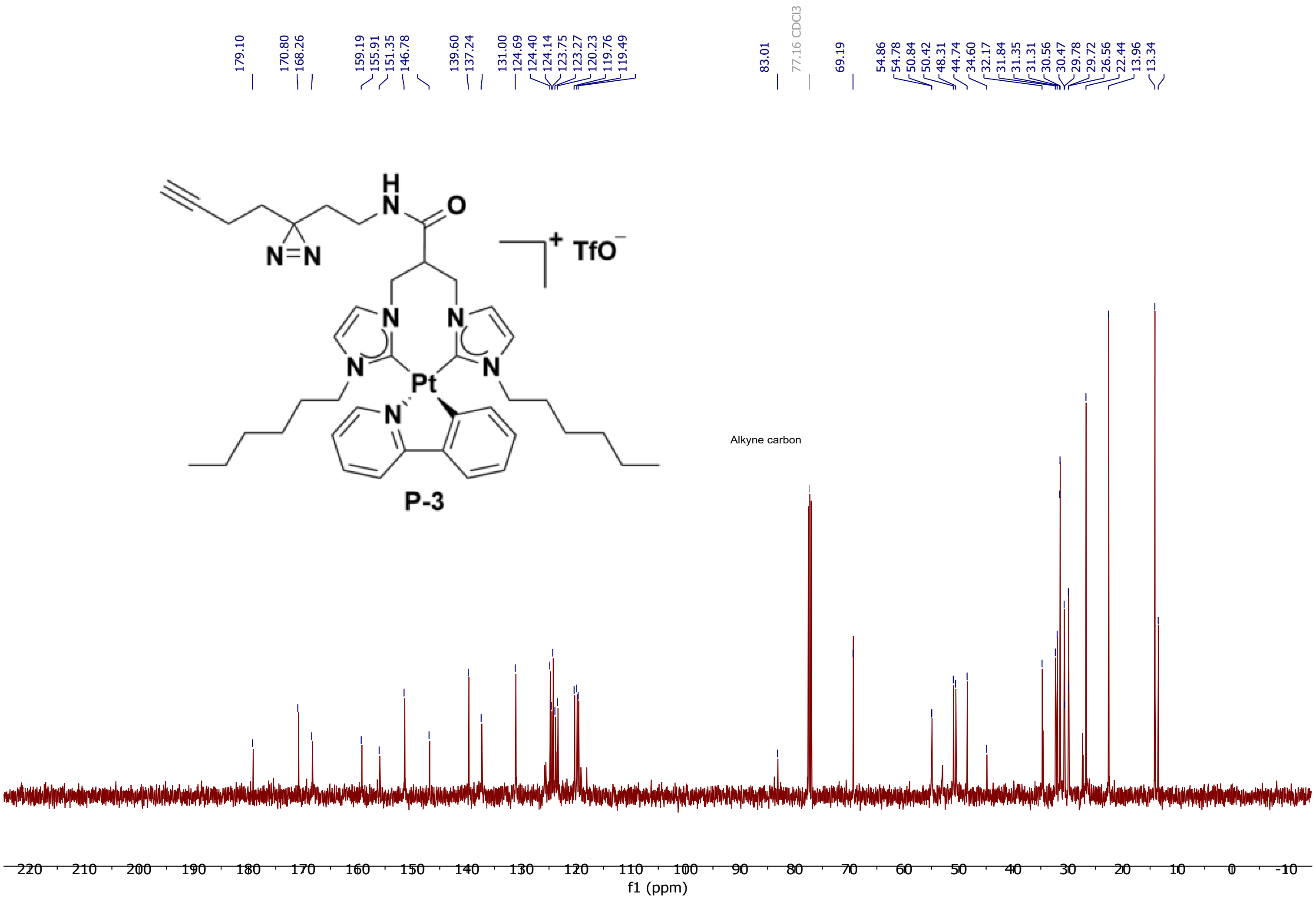

Figure S25. ^{13}C-NMR spectrum of P-3

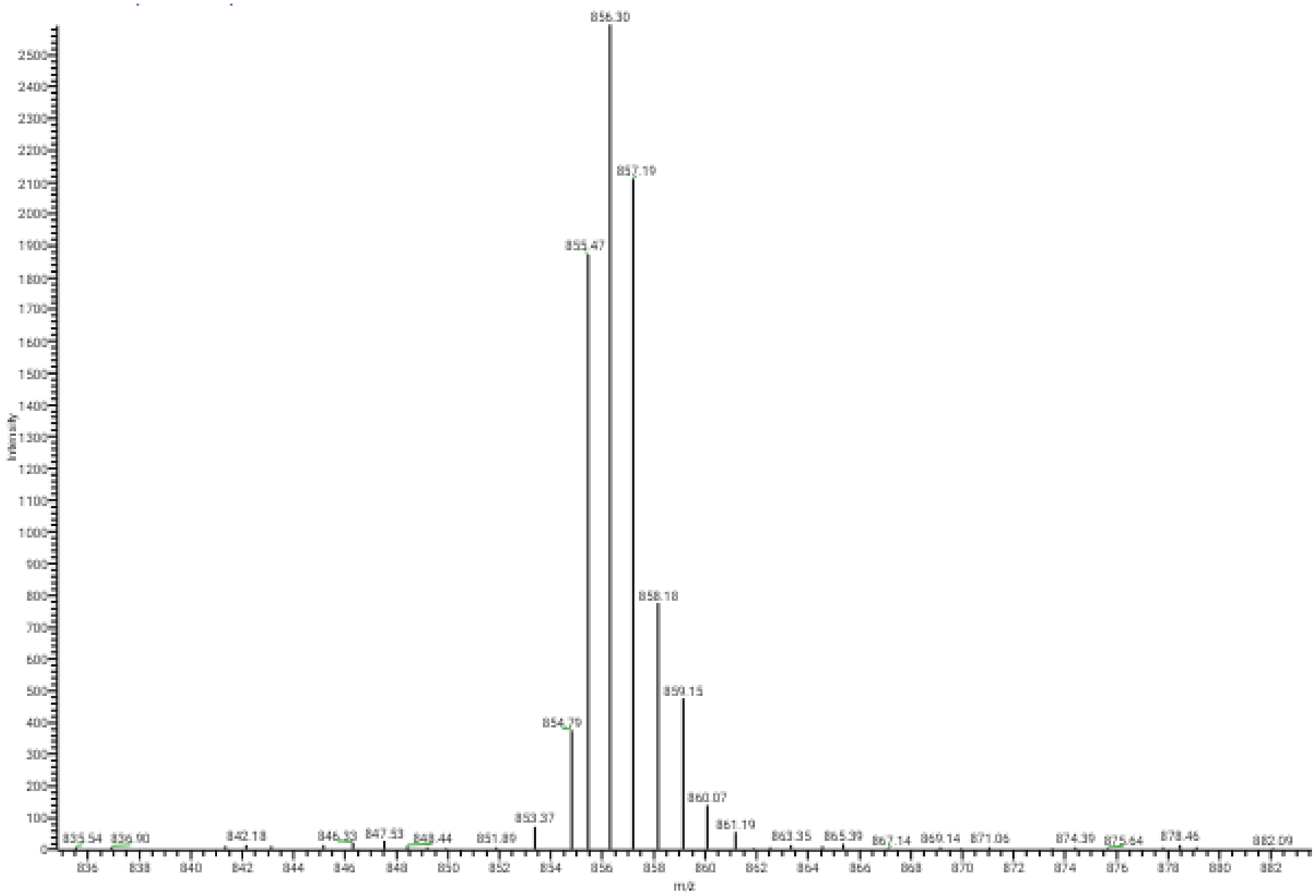

Figure S26. ESI-MS spectrum of P-3

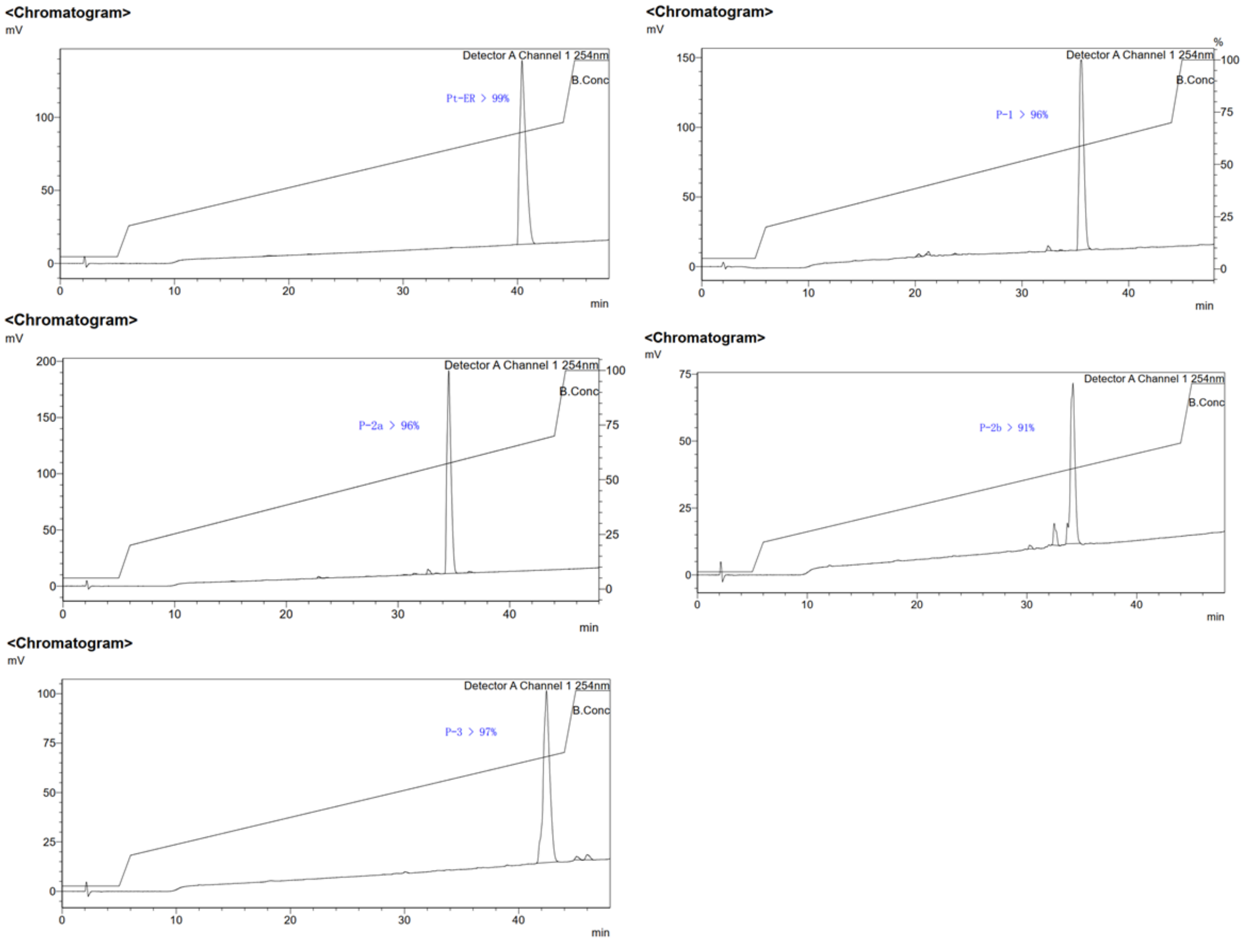

Figure S27. Purity of AfBPs on reverse phase-HPLC.

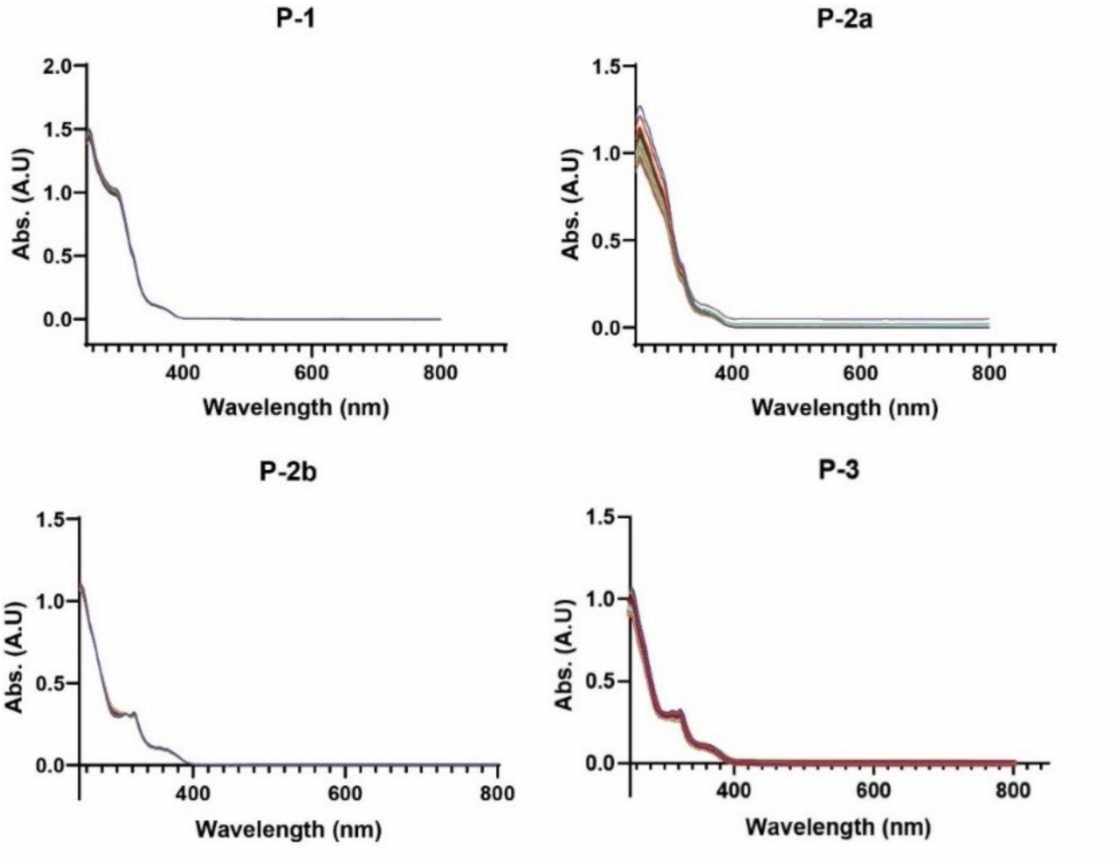

Figure S28. 24-h aqueous stability of AfBPs determined by UV-vis spectrometer. Probes from DMSO stock solutions were dissolved in DI water (containing 10 % ACN, v/v).

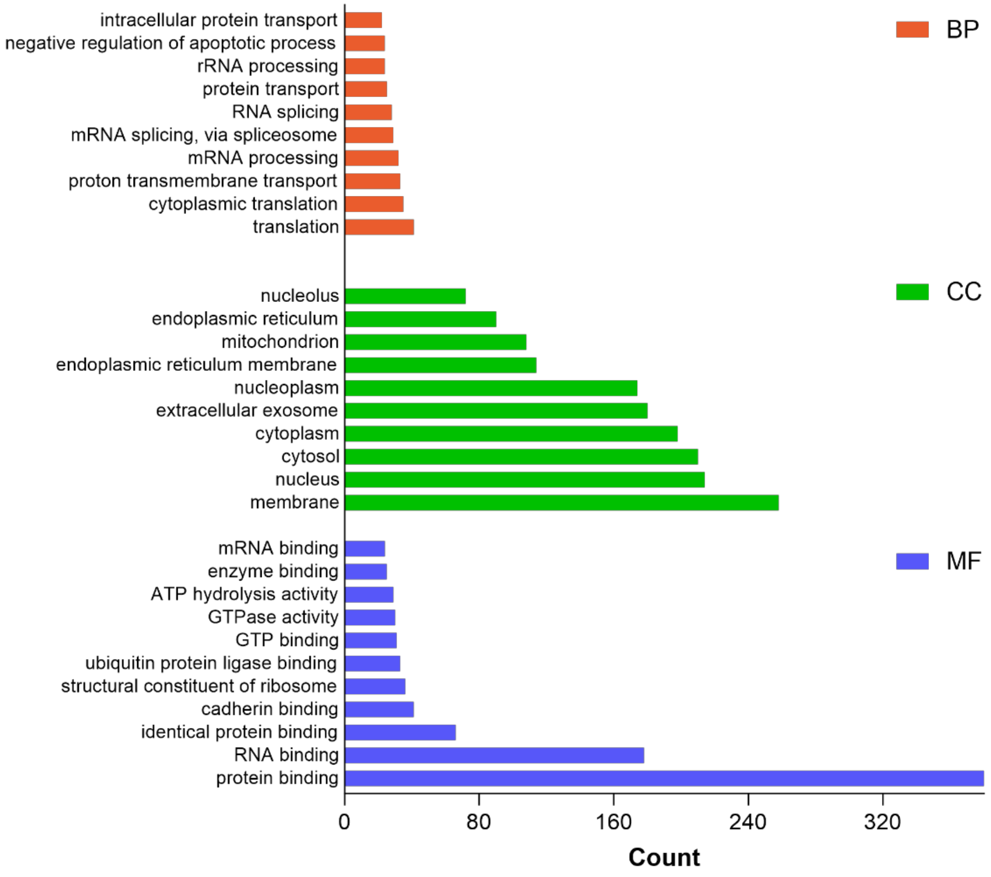

Figure S29. Gene Ontology (GO) enrichment analysis. GO functional enrichment analysis of proteins significantly enriched by all three probes (> 5 fold, $p < 0.0000001$). Top 10 significantly enriched GO items were listed. BP: biological process. CC: cellular component. MF: molecular function. Analysis was performed on National Institutes of Health, DAVID bioinformatics platform.

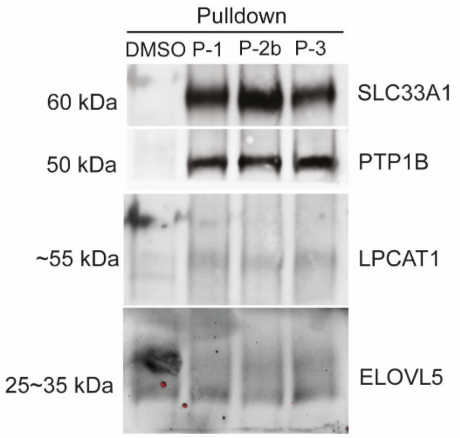

Protein ID	Name	Function
O00400	Solute carrier family 33 member 1 (SLC33A1)	Imports acetyl-CoA from cytosol into the ER lumen
Q8NF37	Lysophosphatidylcholine acyltransferase 1 (LPCAT1)	Exhibits acetyltransferase activity; Modulates lipid metabolism
Q9NYP7	Very long chain fatty acid elongase 5 (ELOVL5)	Catalyzes elongation of long-chain fatty acids; Modulates lipid metabolism
P18031	Protein tyrosine phosphatase 1B (PTP1B)	Dephosphorylates p-PERK; Regulate unfolded protein response in ER

Figure S30. Validation of labeling of potential target proteins, LPCAT and ELOVL5, by immunoblotting and introduction to 4 potential ER protein targets. HCT116 cells were treated with 15 µM probes or DMSO, respectively, followed by UV irradiation for 20 min, click reaction and avidin-based pulldown assay. After multiple washing steps, an equal amount of neutravidin beads were used for immunoblotting assay.

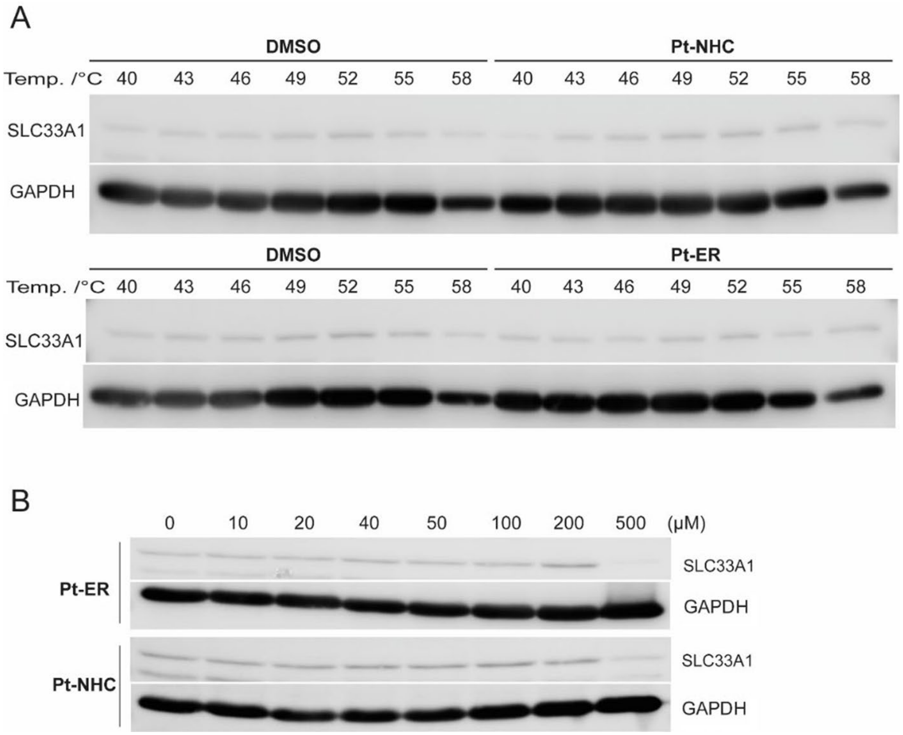

Figure S31. The determination of SLC33A1 thermal stability in the presence of Pt-ER and Pt-NHC. A) Equal amount of HCT116 cell lysates were incubated with 20 µM compounds or DMSO for 1 h, and then heated at indicated temperatures for 3 min. **B)** Isothermal dose-response CETSA (ITDR-CETSA) of SLC33A1 at 49 °C. Equal amount of HCT116 cell lysates were incubated with compounds at indicated concentrations for 1 h. For both experiments, equal amount of soluble fraction was collected for immunoblotting.

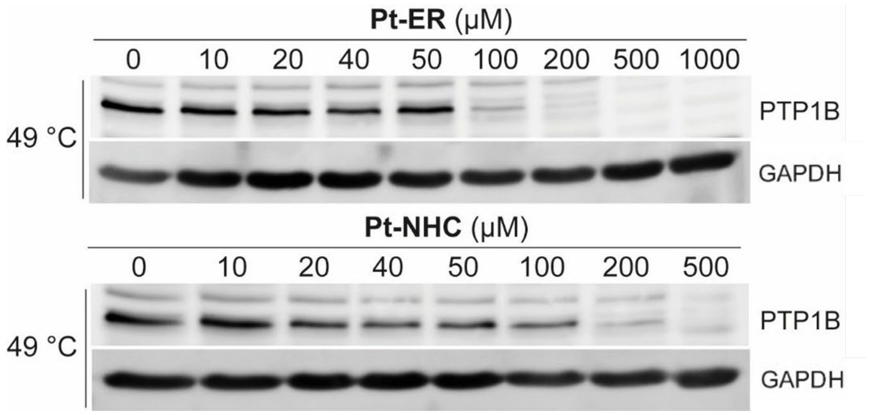

Figure S32. Isothermal dose-response CETSA (ITDR-CETSA) of PTP1B at 49 °C. Equal amount of HCT116 cell lysates were incubated with compounds at indicated concentrations for 1 h. For both experiments, equal amount of soluble fraction was collected for immunoblotting.

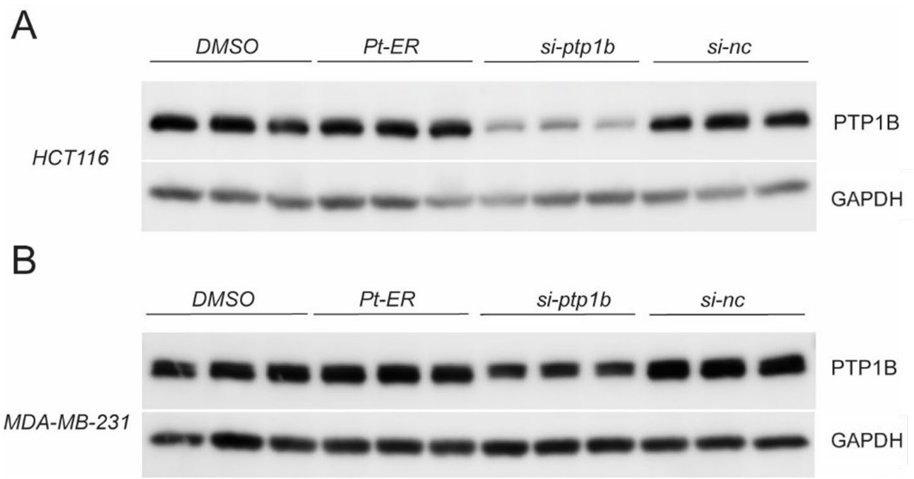

Figure S33. Validation of PTP1B expression levels in cancer cell lines. Western blot analysis of PTP1B protein levels in A) HCT116 and B) MDA-MB-231 cells. For each cell line, the effects of small molecule treatment (DMSO as control, Pt-ER at 10 μM for 4 h) and genetic knockdown (control siRNA si-nc or si-ptp1b for 72 h) are shown. GAPDH was used as a loading control.

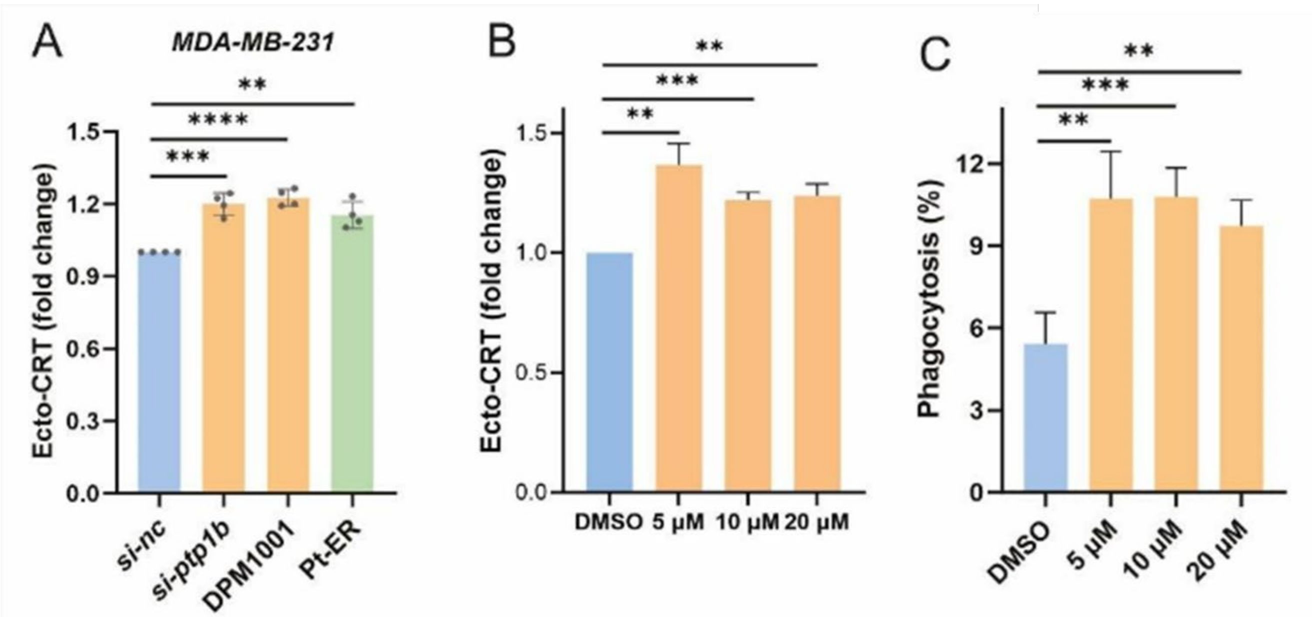

Figure S34. The effects of PTP1B suppression on the immunogenicity of tumor cell death. A) Ecto-CRT level on MDA-MB-231 cells treated with indicated siRNA and indicated compounds (4 h) by flow cytometry. si-nc: nonspecific control siRNA; si-ptp1b: PTP1B-targeting siRNA; TUN: tunicamycin, 10 μg/mL; DPM1001, 20 μM; Pt-ER, 10 μM. Data shown as fold change in MFI normalized to control (si-nc + DMSO). B) Ecto-CRT level on HCT116 cells in response to DPM1001 at different concentrations, 4 h. Data shown as fold change in MFI normalized to DMSO control. C) The percentage of phagocytosis of HCT116 cells induced by DPM1001 at different concentrations for 4 h. Error bars: S.D., n ³ 3. Two tailed, unpaired Student's t test, ns: not significant, *$p < 0.05$, **$p < 0.01$, ***$p < 0.001$, ****$p < 0.0001$.

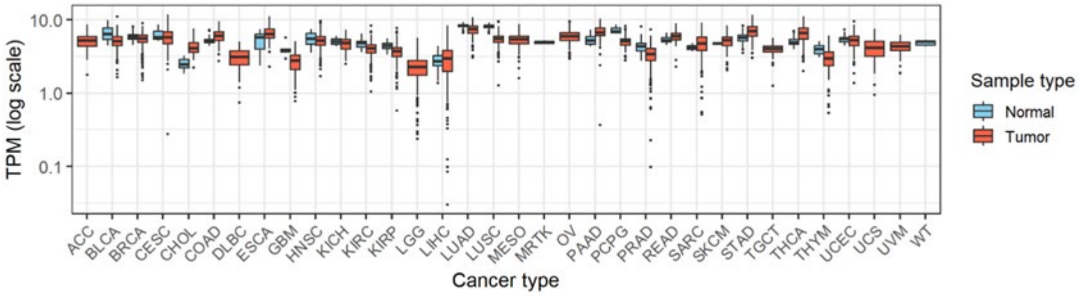

Figure S35. Expression of *PTPN1* across tumor and normal tissues in human cancers. Boxplots show log-transformed TPM values from TCGA, TARGET, and GTEx datasets for 34 cancer types. Tumor-versus-normal comparisons were available for 23 types, with significant differences ($p < 0.05$) observed in 16 of them, as summarized in Figure 6A. Cancer types are ordered alphabetically according to TCGA abbreviations.

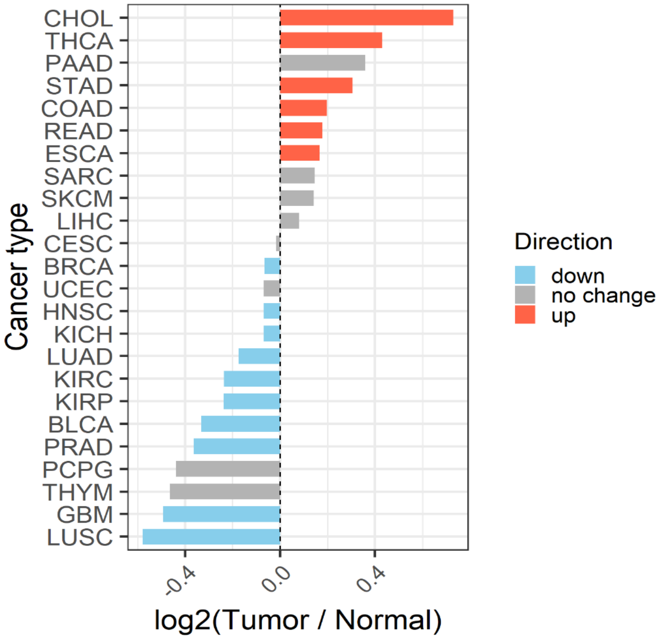

Figure S36. Differential expression of *PTPN1* between tumor and normal tissues across cancer types. Bar plot showing $\log_2$ fold changes (Tumor / Normal) in *PTPN1* transcript levels derived from TCGA, TARGET, and GTEx datasets. Cancer types are ordered by fold-change magnitude. Red bars indicate significant upregulation, blue bars indicate significant downregulation, and gray bars denote no significant difference ($p \geq 0.05$). Significant upregulation was observed in six cancer types and downregulation in ten, consistent with the results shown in Figure 6A.

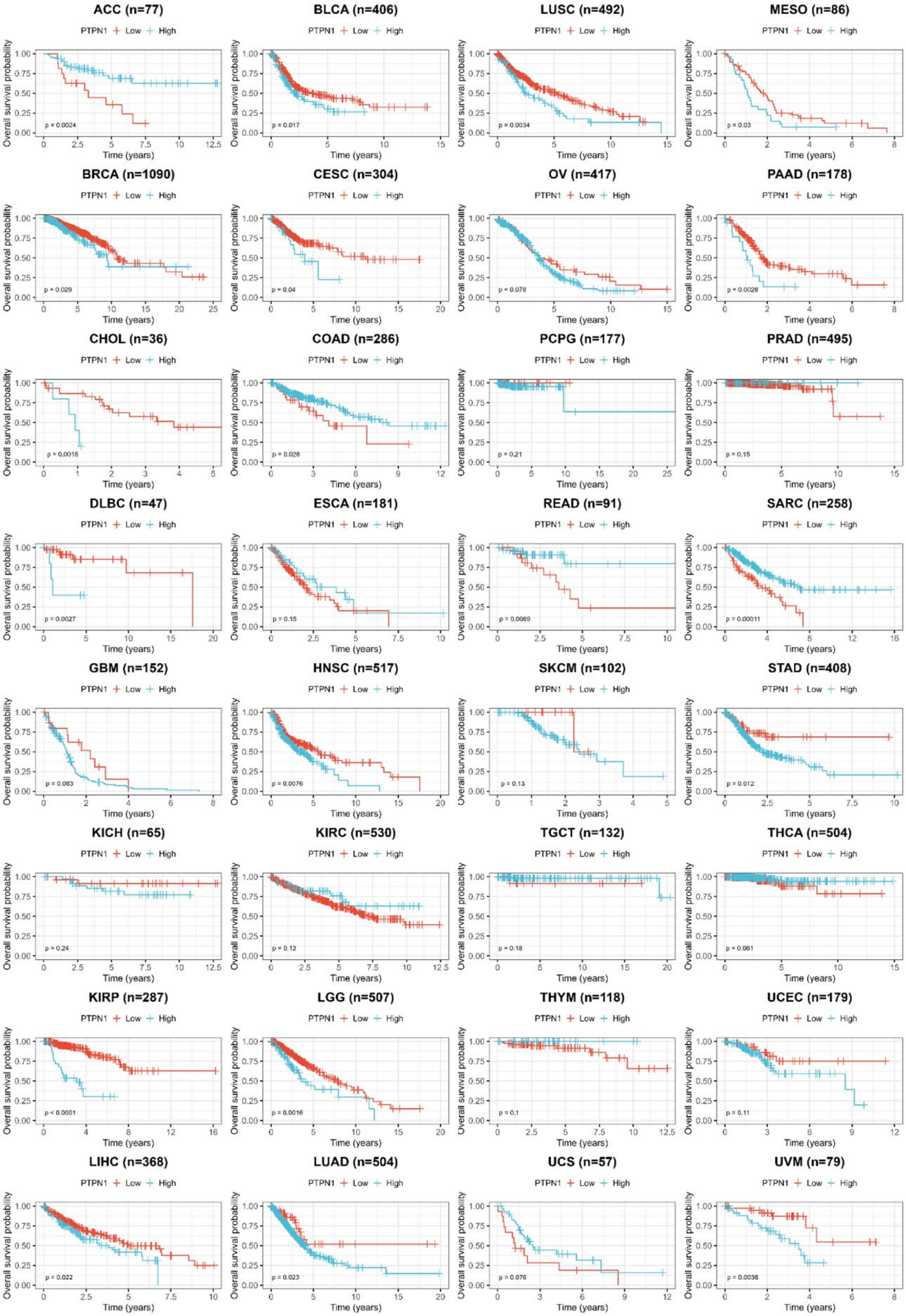
ACC (n=77)
p = 0.0024
BLCA (n=406)
p = 0.017
LUSC (n=492)
p = 0.0034
MESO (n=86)
p = 0.03
BRCA (n=1090)
p = 0.029
CESC (n=304)
p = 0.04
OV (n=417)
p = 0.078
PAAD (n=178)
p = 0.0028
CHOL (n=36)
p = 0.0016
COAD (n=286)
p = 0.028
PCPG (n=177)
p = 0.21
PRAD (n=495)
p = 0.15
DLBC (n=47)
p = 0.0027
ESCA (n=181)
p = 0.15
READ (n=91)
p = 0.0069
SARC (n=258)
p = 0.00011
GBM (n=152)
p = 0.083
HNSC (n=517)
p = 0.0076
SKCM (n=102)
p = 0.13
STAD (n=408)
p = 0.012
KICH (n=65)
p = 0.24
KIRC (n=530)
p = 0.12
TGCT (n=132)
p = 0.18
THCA (n=504)
p = 0.061
KIRP (n=287)
p < 0.0001
LGG (n=507)
p = 0.0016
THYM (n=118)
p = 0.1
UCEC (n=179)
p = 0.11
LIHC (n=368)
p = 0.022
LUAD (n=504)
p = 0.023
UCS (n=57)
p = 0.076
UVM (n=79)
p = 0.0036
PTPN1 + Low + High
Overall survival probability
Time (years)

Figure S37. Pan-cancer survival analysis of *PTPN1* expression across TCGA cohorts. Kaplan–Meier curves showing overall survival probabilities for patients with high (red) or low (blue) *PTPN1* expression across 32 cancer types (n = 10,496). Patient groups were stratified by optimal cutpoints based on the maximally selected rank statistic. *p*-values were determined using the log-rank test. Significant associations ($p < 0.05$) were observed in 22 cancer types.

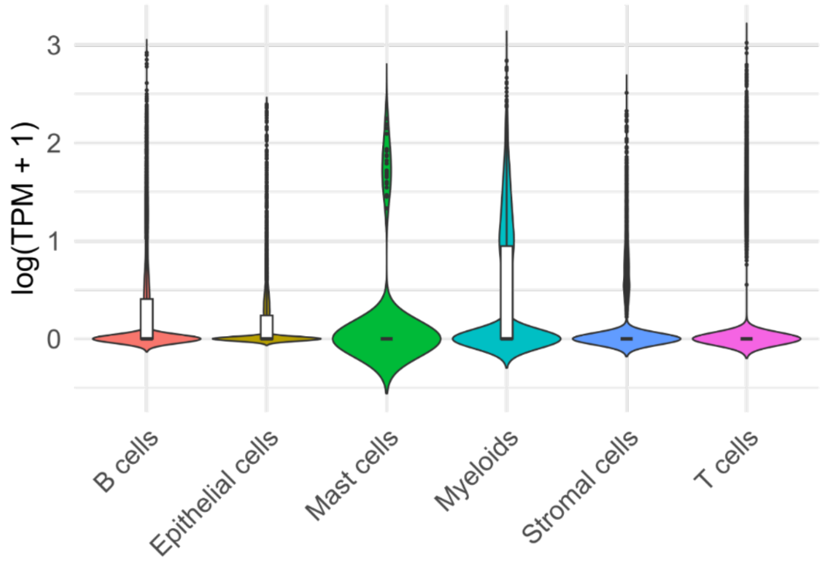

Figure S38. Single-cell expression of *PTPN1* across cell types in colorectal cancer. Violin plots showing log(TPM + 1) expression values of *PTPN1* in annotated cell populations from the GSE132465 single-cell RNA-seq dataset (63,689 cells from 23 tumors and 10 normal mucosa samples). Distinct, cell type–specific expression patterns were observed, with the highest expression detected in myeloid and B cells, followed by epithelial, stromal, mast, and T cells.

References

1. Tham, M. J. R.; Babak, M. V.; Ang, W. H., PlatinER: A Highly Potent Anticancer Platinum(II) Complex that Induces Endoplasmic Reticulum Stress Driven Immunogenic Cell Death. *Angew. Chem. Int. Ed.* **2020,** *59* (43), 19070-19078.
2. Pozzi, C.; Cuomo, A.; Spadoni, I.; Magni, E.; Silvola, A.; Conte, A.; Sigismund, S.; Ravenda, P. S.; Bonaldi, T.; Zampino, M. G.; Cancelliere, C.; Di Fiore, P. P.; Bardelli, A.; Penna, G.; Rescigno, M., The EGFR-specific antibody cetuximab combined with chemotherapy triggers immunogenic cell death. *Nat. Med.* **2016,** *22* (6), 624-31.
3. Xu, C.; Wu, H.; Liu, Y.; Li, F.; Manne, R. K.; Lin, H. K., Protocol for detecting macrophage-mediated cancer cell phagocytosis in vitro and in vivo. *STAR Protoc.* **2023,** *4* (1), 101940.
4. Wong, D. Y.; Ong, W. W.; Ang, W. H., Induction of immunogenic cell death by chemotherapeutic platinum complexes. *Angew. Chem. Int. Ed.* **2015,** *54* (22), 6483-7.
5. Li, X. M.; Huang, S.; Li, X. D., Photo-ANA enables profiling of host-bacteria protein interactions during infection. *Nat. Chem. Biol.* **2023,** *19* (5), 614-623.
6. Reisman, B. J.; Guo, H.; Ramsey, H. E.; Wright, M. T.; Reinfeld, B. I.; Ferrell, P. B.; Sulikowski, G. A.; Rathmell, W. K.; Savona, M. R.; Plate, L.; Rubinstein, J. L.; Bachmann, B. O., Apoptolidin family glycomacrolides target leukemia through inhibition of ATP synthase. *Nat. Chem. Biol.* **2022,** *18* (4), 360-367.
7. Kawatkar, A.; Clark, R. A.; Hopcroft, L.; Roaquin, D. A.; Tomlinson, R.; Zuhl, A. M.; Lamont, G. M.; Kettle, J. G.; Critchlow, S. E.; Castaldi, M. P.; Goldberg, F. W.; Zhang, A. X., Chemical Biology Approaches Confirm MCT4 as the Therapeutic Target of a Cellular Optimized Hit. *ACS Chem. Biol.* **2023,** *18* (2), 296-303.
8. Cheng, Y. S.; Zhang, T.; Ma, X.; Pratuangtham, S.; Zhang, G. C.; Ondrus, A. A.; Mafi, A.; Lomenick, B.; Jones, J. J.; Ondrus, A. E., A proteome-wide map of 20(S)-hydroxycholesterol interactors in cell membranes. *Nat. Chem. Biol.* **2021,** *17* (12), 1271-1280.
9. Dayalan Naidu, S.; Dikovskaya, D.; Moore, T. W.; Dinkova-Kostova, A. T., Detection of thermal shift in cellular Keap1 by protein-protein interaction inhibitors using immunoblot- and fluorescence microplate-based assays. *STAR Protoc.* **2022,** *3* (2), 101265.
10. Dziekan, J. M.; Wirjanata, G.; Dai, L.; Go, K. D.; Yu, H.; Lim, Y. T.; Chen, L.; Wang, L. C.; Puspita, B.; Prabhu, N.; Sobota, R. M.; Nordlund, P.; Bozdech, Z., Cellular thermal shift assay for the identification of drug-target interactions in the Plasmodium falciparum proteome. *Nat. Protoc.* **2020,** *15* (6), 1881-1921.
11. Nagasawa, I.; Muroi, M.; Kawatani, M.; Ohishi, T.; Ohba, S. I.; Kawada, M.; Osada, H., Identification of a Small Compound Targeting PKM2-Regulated Signaling Using 2D Gel Electrophoresis-Based Proteome-wide CETSA. *Cell Chem. Biol.* **2020,** *27* (2), 186-196 e4.
12. Choy, M. S.; Li, Y.; Machado, L.; Kunze, M. B. A.; Connors, C. R.; Wei, X.; Lindorff-Larsen, K.; Page, R.; Peti, W., Conformational Rigidity and Protein Dynamics at Distinct Timescales Regulate PTP1B Activity and Allostery. *Mol. Cell* **2017,** *65* (4), 644-658 e5.
13. Zou, T.; Lok, C. N.; Fung, Y. M.; Che, C. M., Luminescent organoplatinum(II) complexes containing bis(N-heterocyclic carbene) ligands selectively target the endoplasmic reticulum and induce potent photo-toxicity. *Chem Commun (Camb)* **2013,** *49* (47), 5423-5.
